# Induced Pluripotent Stem Cells Reprogrammed with Three Inhibitors Show Accelerated Differentiation Potentials with High Levels of 2-Cell Stage Marker Expression

**DOI:** 10.1016/j.stemcr.2018.12.018

**Published:** 2019-01-31

**Authors:** Koji Nishihara, Takahiro Shiga, Eri Nakamura, Tomohiko Akiyama, Takashi Sasaki, Sadafumi Suzuki, Minoru S.H. Ko, Norihiro Tada, Hideyuki Okano, Wado Akamatsu

**Affiliations:** 1 Department of Physiology, Keio University School of Medicine, 35 Shinanomachi, Shinjuku-ku, Tokyo 160-8582, Japan; 2 Center for Genomic and Regenerative Medicine, Juntendo University School of Medicine, 2-1-1 Hongo, Bunkyo-ku, Tokyo 113-8421, Japan; 3 Research Institute for Diseases of Old Age, Juntendo University School of Medicine, 2-1-1 Hongo, Bunkyo-ku, Tokyo 113-8421, Japan; 4 Department of Systems Medicine, Sakaguchi Laboratory, Keio University School of Medicine, 35 Shinanomachi, Shinjuku-ku, Tokyo 160-8582, Japan; 5 Center for Supercentenarian Medical Research, Keio University School of Medicine, 35 Shinanomachi, Shinjuku-ku, Tokyo 160-8582, Japan

**Keywords:** induced pluripotent stem cells (iPSCs), culture conditions, 3i, differentiation potentials, Zscan4, 2-cell genes

## Abstract

Although pluripotent stem cells can generate various types of differentiated cells, it is unclear why lineage-committed stem/progenitor cells derived from pluripotent stem cells are decelerated and why the differentiation-resistant propensity of embryonic stem cell (ESC)/induced pluripotent stem cell (iPSC)-derived cells is predominant compared with the *in vivo* equivalents derived from embryonic/adult tissues. In this study, we demonstrated that iPSCs reprogrammed and maintained with three chemical inhibitors of the fibroblast growth factor 4-mitogen-activated protein kinase cascade and GSK3β (3i) could be differentiated into all three germ layers more efficiently than the iPSCs reprogrammed without the 3i chemicals, even though they were maintained with 3i chemicals once they were reprogrammed. Although the iPSCs reprogrammed with 3i had increased numbers of Zscan4-positive cells, the Zscan4-positive cells among iPSCs that were reprogrammed without 3i did not have an accelerated differentiation ability. These observations suggest that 3i exposure during the reprogramming period determines the accelerated differentiation/maturation potentials of iPSCs that are stably maintained at the distinct state.

## Highlights

• iPSCs reprogrammed and maintained with 3i have accelerated differentiation potential
• iPSC colonies reprogrammed with 3i contain increased number of Zscan4 (+) cells

## Graphical Abstract

**Figure:**
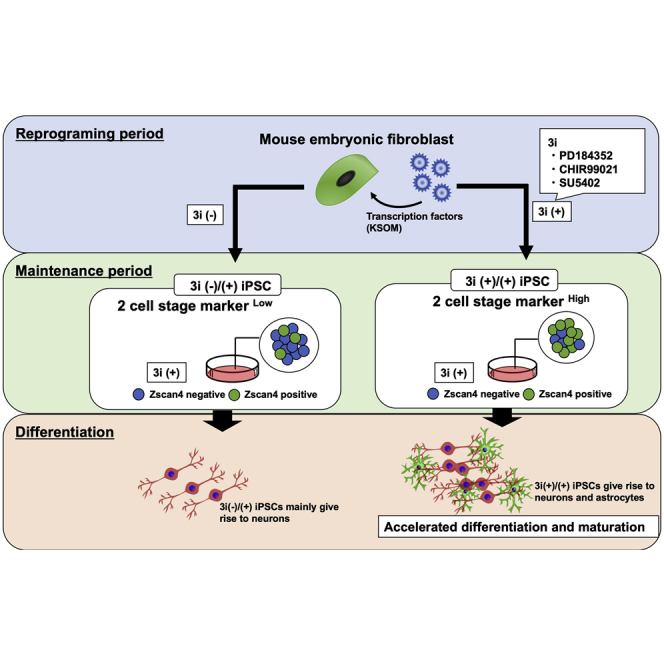

## Teaser

Mouse iPSCs reprogrammed and maintained with three chemical inhibitors of the FGF4-MAPK cascade and GSK3β (3i; PD184352, CHIR99021, and SU5402) could be differentiated into all three germ layers efficiently and contain increased numbers of Zscan4, a 2-cell stage marker, positive cells.

## Introduction

Pluripotent stem cells (PSCs) can theoretically differentiate into derivatives of all three germ layers. Both induced pluripotent stem cells (iPSCs) and embryonic stem cells (ESCs) give rise to lineage-committed somatic stem/progenitor cells and are eventually differentiated into terminally differentiated progenies. Cell replacement therapy, drug screening, and disease modeling are facilitated by the pluripotency and self-renewal ability of these PSCs, which can induce various disease-relevant cell types (Shi et al., 2017, Takahashi and Yamanaka, 2006). However, it is still unclear whether lineage-committed stem/progenitor cells derived from embryonic/adult tissues and PSCs have identical differentiation abilities. A clear difference between tissue- and PSC-derived cells is observed during differentiation into the differentiated progenies. This observation is especially true for human PSCs, where a period of approximately 2–5 months is required for *in vitro* differentiation into hepatocytes (Ma et al., 2013), oligodendrocytes (Numasawa-Kuroiwa et al., 2014), or retinal pigment epithelia (Jin et al., 2011). These observations strongly suggest that the differentiation/maturation of PSC-derived cells is significantly slower than that of equivalents in *in vitro* primary cultures. Regarding neural differentiation *in vitro*, whereas embryonic day 6.5 (E6.5) mouse epiblast-derived neurospheres acquire a responsiveness to fibroblast growth factor (FGF)/epidermal growth factor (EGF) that is characteristic of mature (committed) definitive neural stem cells (NSCs) after a few passages, ESC-derived neurospheres are unable to acquire FGF dependency, even after recurrent passages without exogenous active Notch transduction (Hitoshi et al., 2004). This slow and inefficient differentiation/maturation of PSC-derived cells leads not only to difficulties in the preparation of the desired cells but also to aneuploidy or tumor formation due to the long *in vitro* cultivation period (Conti and Cattaneo, 2010). However, for the cell-based therapy of several diseases with progressive and changeable features (e.g., spinal cord injury [Nagoshi and Okano, 2017], ischemic stroke [Tornero et al., 2013], or acute myocardial infarction [Nelson et al., 2009]), rapid preparations of donor cells are necessary due to limited therapeutic windows of time. Therefore, it may be difficult to prepare iPSC-derived cells for autologous and allogeneic transplantations, and cells may need to be selected despite the risk of immunorejection and infection for these diseases. To contribute to the future regenerative medicine, we aimed to solve this problem by establishing iPSCs with fast and efficient differentiation or maturation potentials compared with the iPSCs that are established by current protocols.

Recent studies have demonstrated that some chemical cocktails containing FGF4- mitogen-activated protein kinase (MAPK) cascade/GSK3β inhibitors (so-called 2i and 3i) contribute to the authentic and homogeneous naive pluripotency of iPSCs (Choi et al., 2017, Marks et al., 2012, Ying et al., 2008) and promote reprogramming efficiency (Silva et al., 2008, Valamehr et al., 2014). Although a few studies have claimed that conversion into a ground (or ground-like) state improves the differentiation potentials of iPSCs (Duggal et al., 2015, Honda et al., 2013), the effect of these chemicals on the differentiation potency of iPSCs remains controversial (Chan et al., 2013, Gafni et al., 2013, Takashima et al., 2014, Theunissen et al., 2014, Valamehr et al., 2014). Given that the mechanism for acquiring pluripotency is drastic epigenetic reprogramming and that the epigenetic memory of the original somatic cells in iPSCs influences their differentiation potential, we hypothesized that the addition of these chemicals during a reprogramming period influenced the *in vitro* differentiation/maturation potential of iPSCs. To test this hypothesis, we generated two groups of murine iPSCs using these chemicals during two different periods (only a maintenance period or both a reprogramming and maintenance period) and found that their differentiation potentials are significantly different.

## Results

### Generation of Murine iPSCs with Pluripotency-Enhancing Chemicals

First, we speculated that the reprogramming period, not the maintenance period, in clonally generated iPSC lines could influence the differentiation/maturation potential. To test whether using chemicals that support cellular reprogramming and/or pluripotency during the reprogramming period could regulate the differentiation potentials of iPSCs, we used these chemicals during cellular reprogramming into iPSCs with different time courses. We used three chemicals that inhibit FGF receptor tyrosine kinase (SU5402), ERK1/2 (PD184352 or PD0325901), and GSK3β (CHIR99021) as representative chemical molecules that support pluripotency (Ying et al., 2008).

**Figure 1:**
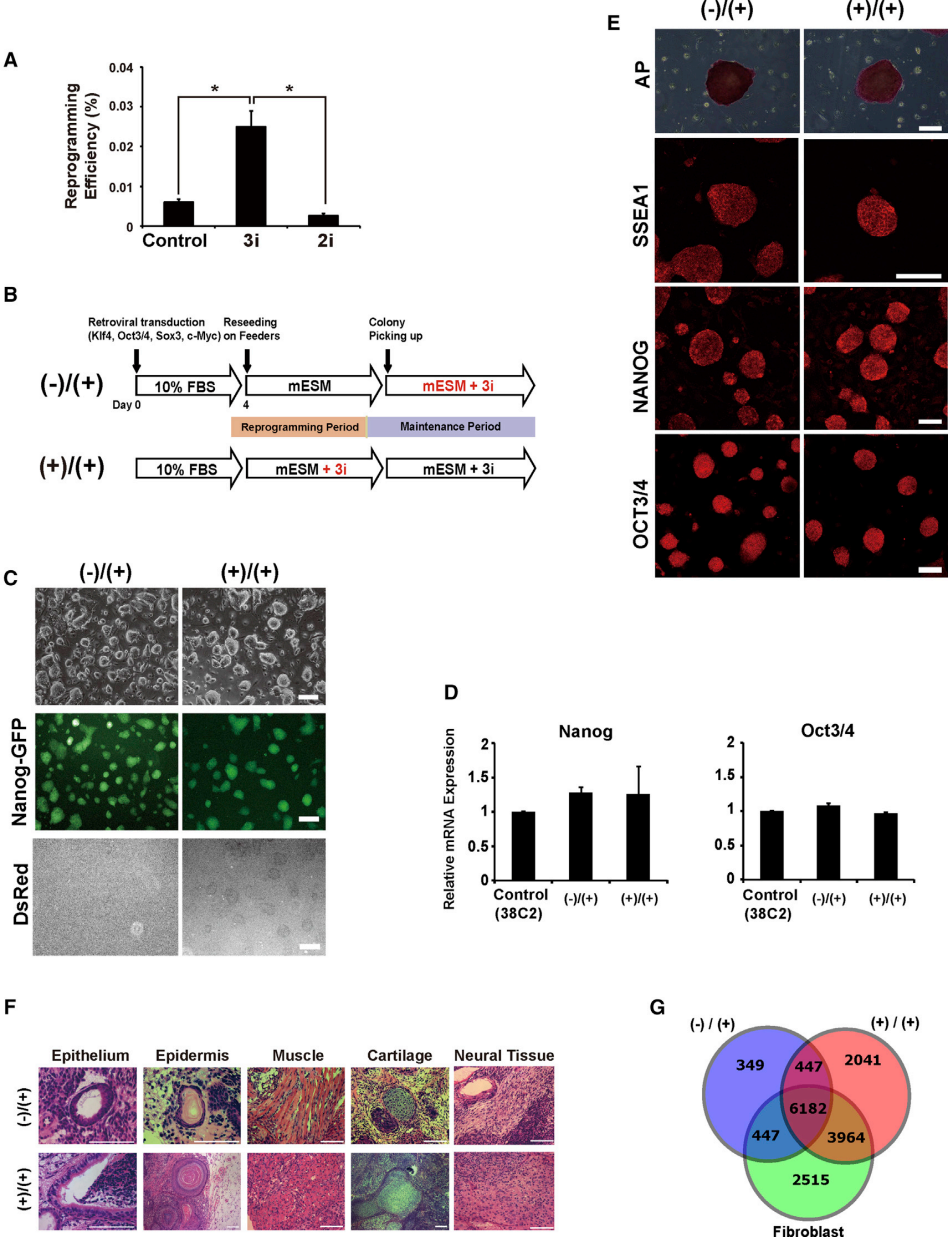
Generation of Two Groups of Murine iPSCs Using Small Molecules (A) The reprogramming efficiency of 3i- or 2i-treated fibroblasts compared with untreated cells is shown. iPSC colonies were identified based on ESC-like morphology and expression of *Nanog-GFP* detected under a fluorescence microscope (n = 3; ^∗^p < 0.05). (B) The schematic representations of two reprogramming protocols used in this study. In our protocol, *Nanog-GFP* transfected fibroblasts were cultured in 10% FBS medium, re-seeded on feeders, and switched to murine ESC medium. While the 3i chemicals were used only after colonies were picked (maintenance period) in the (−)/(+) condition, the 3i chemicals were used during both the reprogramming phase (including the period from day 2 to day 12) and the maintenance phase in the (+)/(+) condition. The 3i chemicals were used at the following concentrations: 3 μM CHIR99021, 0.8 μM PD184352, and 0.8 μM SU5402. (C) Representative images of iPSC colonies generated using the (−)/(+) or (+)/(+) protocol. Both colony types exhibited a typical ESC morphology and expressed *Nanog-GFP* homogeneously. The silencing efficiency of dsRed, which was transduced together with OSKM as a transgene control while generating the iPSCs, was verified. Scale bar, 100 μm. (D) qPCR for expression of pluripotency markers (*Nanog* and *Oct3/4*) in iPSCs. The averages and SD of three different clones of the (−)/(+) or (+)/(+) iPSC lines are represented. 38C2 is a control iPSC clone that was derived from the same *Nanog-*GFP mouse line (n = 3; ^∗^p < 0.05). (E) Representative images of AP staining and immunofluorescence staining of pluripotent markers (SSEA1, Nanog, and OCT3/4) in both the (−)/(+) and (+)/(+) iPSC lines. Scale bars, 200 μm (AP staining) and 100 μm (immunofluorescence staining). (F) H&E staining of teratomas derived from (−)/(+) or (+)/(+) iPSCs. Cells were transplanted into the testes of severe combined immunodeficiency mice. After 3 weeks, tumors were sectioned. Gut-like epithelial tissues (left), epidermal tissues, striated muscles, cartilage, and neural tissues (right) are shown. Scale bars, 200 μm. (G) The results of comprehensive DNA methylation analysis with MBD-seq. Venn diagram of unique and shared genes with methylated regions among (−)/(+) iPSCs (blue), (+)/(+) iPSCs (red), and fibroblasts (green) (n = 3, FDR < 0.05). Error bars represent mean ± SEM from three independent experiments (n = 3).

First, we tested whether 2i (PD0325901 and CHIR99021) or 3i (PD184352, CHIR99021, and SU5402) had any effects on reprogramming efficiency and on maintenance of pluripotency. We reprogrammed mouse embryonic fibroblasts (MEFs) derived from *Nanog-*GFP-IRES-Puro transgenic mice (Okita et al., 2007) using retroviruses harboring four transcription factors: *Oct3/4*, *Sox2*, *Klf4*, and *c-Myc* (KSOM). dsRed transgenes were also infected simultaneously as an indicator of transgene silencing. We began to add 2i/3i on day 4 after infection because previous reports demonstrated that KSOM-transduced MEFs underwent a mesenchymal-to-epithelial transition around day 5 after infection in the initiation phase, followed by the expression of SSEA1 and NANOG in the maturation phase (Li et al., 2010, Polo et al., 2010). We quantified the number of generated GFP^+^ dsRed^−^ ESC-like colonies during reprogramming with or without 2i/3i and revealed that 3i increased the number of GFP^+^ dsRed^−^ ESCs, in the form of colonies, when examined at 3 weeks post-infection, while 2i had no significant effect on colony formation efficiency (Figure 1A). These data suggested that the addition of 3i during the reprogramming period enhanced the reprogramming efficiency and increased the number of colonies compared with the conventional condition without 3i. We hypothesized that the higher number of colonies that appeared with the sequential addition of 3i during the reprogramming and maintenance period would not appear in the conventional condition without 3i. Thus, we used the 3i chemicals as the model for the reprogramming molecules in this study and investigated the relationship between the reprogramming conditions and differentiation potential of iPSCs.

To exclude the possibility that the differences between the 3i (−) and 3i (+) iPSCs resulting from 3i usage during the reprogramming phase might be masked by 3i usage during the maintenance phase, both the 3i (−) and 3i (+) iPSCs were maintained with 3i after the reprogramming phase and are denoted as (−)/(+) and (+)/(+) iPSCs, respectively (Figure 1B). We isolated 3 clones of (−)/(+) iPSCs and 11 clones of (+)/(+) iPSCs, and all clones from both conditions exhibited similar morphologies. All clones expressed the *Nanog-*GFP fluorescence reporter but did not express dsRed due to complete silencing of the transgenes (Figure 1C). Three iPSC clones from each group, (−)/(+) and (+)/(+), were analyzed in this study. qRT-PCR analysis showed that the mRNA expression levels of pluripotent cell marker genes *Nanog* and *Oct3/4* were similar to those of control murine iPSCs (38C2), which were derived from *Nanog-*GFP transgenic animals (Okita et al., 2007) (Figure 1D). RT-PCR analysis revealed that the four transgenes were successfully silenced (Figure S1). Both the (−)/(+) and (+)/(+) colonies had a typical ESC morphology and were stained with alkaline phosphatase (AP). The expression of pluripotency markers, including NANOG and SSEA1 in both the (−)/(+) and (+)/(+) colonies was confirmed by immunocytochemical staining (Figure 1E). Teratoma formation analysis demonstrated that all generated iPSCs had similar developmental potentials to differentiate into all three germ layers (Figures 1F and S1C). We also confirmed characteristics of (−)/(+) and (+)/(+) cells as PSCs by blastocyst injection (Figure S1D). Although the efficiency of chimeras seemed relatively low, perhaps due to fetal bovine serum (FBS) usage in iPSCs, high passage numbers (more than 40) and non-biased iPSC clonal selection, we were able to obtain several chimeric animals from (−)/(+) and (+)/(+) cells. CGH array analysis demonstrated that genetic alteration frequencies were similar between (−)/(+) and (+)/(+) iPSCs (Figure S1B). To examine the global DNA methylation status of (−)/(+), (+)/(+) iPSCs and their original fibroblasts, we performed genome-wide analysis using methyl-CpG-binding domain protein 2 (MBD2)-mediated methylated DNA enrichment followed by deep sequencing (MBD-seq) (Shimamoto et al., 2014). The peaks of mapped tags were defined as methylated regions, and we compared whole-genome methylation levels. The amount of methylated DNA-enriched in (+)/(+) iPSCs was higher than that in (−)/(+) iPSCs and original fibroblasts (Table S2). These results indicated that both the (−)/(+) and (+)/(+) iPSCs were indistinguishable by pluripotency and genomic structures but have distinctly different global methylation statuses.

### Accelerated Differentiation of iPSCs Established with 3i during the Reprogramming Period

**Figure 2:**
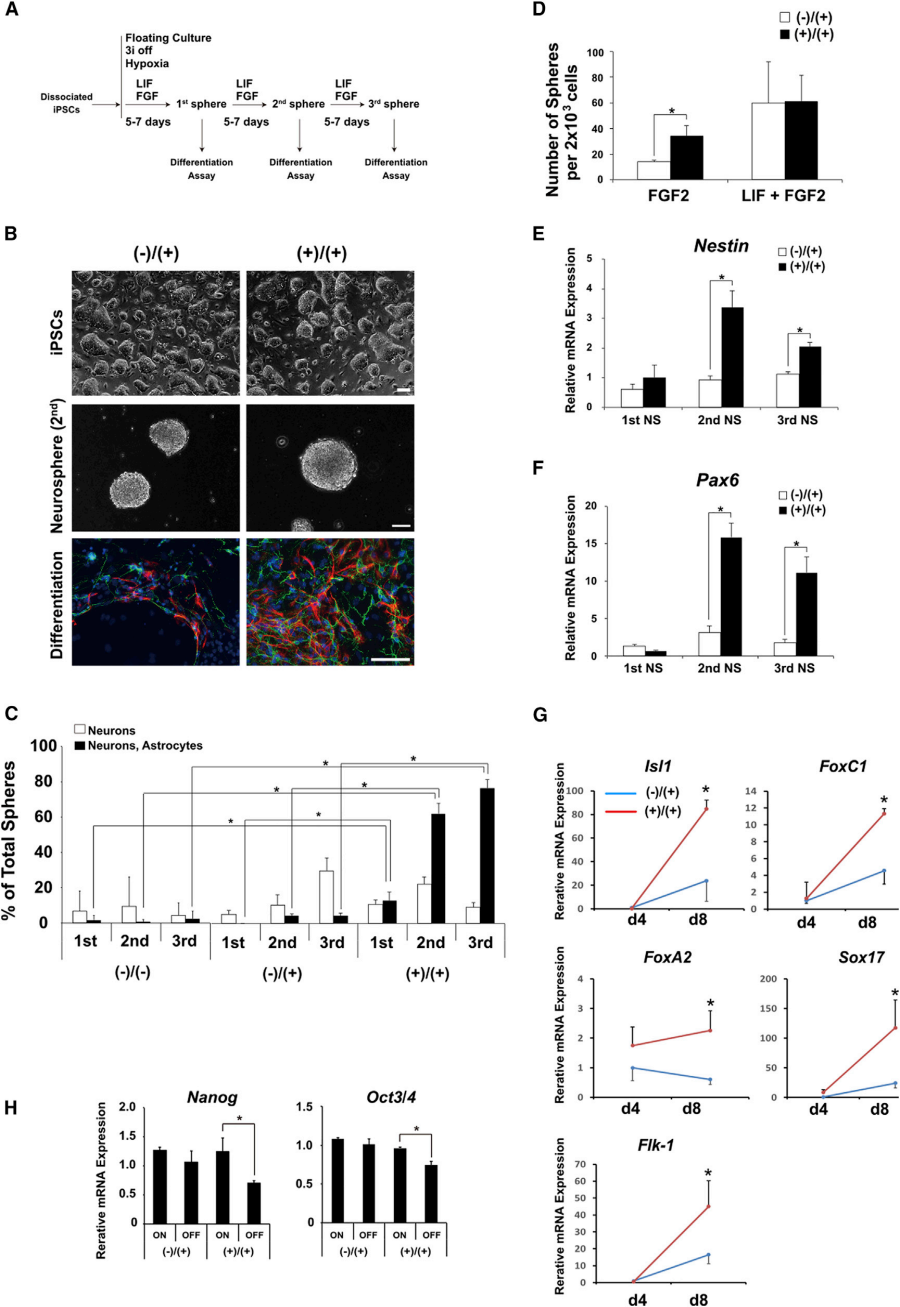
Differentiation Potentials of iPSCs Generated with or without the 3i Chemicals (A) Schematic representation of the strategy for differentiation of iPSCs to the neural lineage. iPSCs were dissociated and cultured using a suspension method to form neurospheres that were treated with LIF and basic FGF2. The resulting neurospheres were transferred onto a poly-L-ornithine/fibronectin-coated chambered slide glass to differentiate into neurons and astrocytes. (B) Representative images of (−)/(+) and (+)/(+) iPSCs, iPSC-derived neurospheres, and immunocytochemical stains of neural cell marker proteins in the differentiated neuronal and glial cells derived from neurospheres are shown. Scale bars, 100 μm. (C) Differentiation efficiency of the first, second, and third neurospheres derived from (−)/(−), (−)/(+), and (+)/(+) iPSCs. The frequency of colonies consisting of neurons (βΙΙΙ-tubulin) and/or astrocytes (GFAP) was evaluated by immunocytochemistry and is presented as the percentage of positive colonies (n = 3; ^∗^p < 0.05). (D) Second neurospheres derived from (−)/(+) or (+)/(+) iPSCs were dissociated (to a final cell density of 10 cells/μL) and grown to form neurospheres in the presence of LIF + FGF2 or FGF2 alone. The number of subcloned spheres per 2 × 10^3^ cells is shown (n = 3; ^∗^p < 0.05). (E and F) mRNA expression of markers for neural progenitors, *Nestin* (E) and *Pax6* (F) in the first, second, and third neurospheres was analyzed by qRT-PCR. The data are presented as the expression relative to that in (−)/(+) iPSCs (n = 3; ^∗^p < 0.05). (G) Gene expression of early mesodermal (*Isl1* and *FoxC1*) and endodermal (*Sox17*, *FoxA2*, and *Flk-1*) markers in EBs derived from (−)/(+) and (+)/(+) iPSCs was analyzed by qRT-PCR. The data are presented as the expression relative to that in (−)/(+) iPSC-derived EBs on day 4 (n = 5; ^∗^p < 0.05). (H) Relative mRNA expression of pluripotency markers (*Nanog* and *Oct3/4*) in (−)/(+) and (+)/(+) iPSCs before or after withdrawal of 3i chemicals. The averages and SD of three different clones of (−)/(+) or (+)/(+) iPSC clones are represented (n = 3, ^∗^p < 0.05). Error bars represent mean ± SEM from three or five independent experiments (n = 3 or 5).

Next, we analyzed the maturation and differentiation capacities of NSCs derived from both the (−)/(+) and (+)/(+) iPSC clones. By using the neurosphere formation assay, we evaluated the response to growth factors (FGF2 and leukemia inhibitory factor [LIF]) and the distribution of the differentiated progenies (neurons and astrocytes). As previously reported (Akamatsu et al., 2009, Hitoshi et al., 2004, Hitoshi et al., 2002, Tropepe et al., 2001), pluripotent cells first differentiate into LIF-dependent primitive NSCs (pNSCs) and then gradually lose LIF dependency to develop into FGF-dependent definitive NSCs (Tropepe et al., 2001). The schematic schedule for neural differentiation is shown in Figure 2A. After separating from the feeder cells, iPSCs were dissociated into a low-density single-cell suspension. Dissociated iPSCs were converted to pNSCs in the presence of LIF. Then, pNSCs formed primary neurospheres after cultivation for 5–7 days in serum-free medium containing LIF and FGF2. To form secondary and tertiary neurospheres, floating spheres were dissociated into single cells and cultured in the serum-free medium that contained LIF and FGF2. Secondary and tertiary neurospheres appeared 5–7 days after passaging.

Both the (−)/(+) and (+)/(+) iPSC clones formed floating spheres that were morphologically similar to each other (Figure 2B, upper and middle panels). To compare the differentiation capacity of the NSCs derived from the (−)/(+) and (+)/(+) iPSCs, the primary, secondary and tertiary neurospheres were dissociated and subjected to 7 days of adherent culture without growth factors to undergo neural differentiation (Figure 2B, lower panels). The frequencies by which neurospheres gave rise to neurons and/or astrocytes were quantified by immunocytochemistry using anti-βIII-tubulin and GFAP antibodies, respectively (Figures 2C and S2A). As another control, we analyzed NSCs derived from (−)/(−) iPSC clones that were considered as conventional iPSC clones that were generated and maintained without 3i chemicals. The primary neurospheres derived from (−)/(+) iPSC clones mainly gave rise to neurons, and few primary neurospheres that generated astrocytes were found. Although astrocytes were found in the differentiated cells from the secondary and tertiary neurospheres, frequency was very low (Figures 2C and S2A). We also examined the differentiation properties of tertiary neurospheres derived from iPSCs reprogrammed with 1i (SU5402) and 2i (PD184352 and CHIR99021). Neither 1i nor 2i iPSCs exhibited similar accelerated differentiation into neurons and astrocytes as 3i (+)/(+) iPSCs, suggesting that 3i reprogramming has the largest effect on acceleration of differentiation of iPSCs (Figures S2B and S2E).

These results were similar to those previously described for ESCs (Naka et al., 2008, Okada et al., 2008), suggesting that NSCs in the primary neurospheres derived from iPSCs have early neurogenic characteristics as do those from ESCs.

In contrast, neurospheres derived from (+)/(+) iPSC clones gave rise to a significantly increased number of astrocytes at all passages compared with those from the (−)/(+) and (−)/(−) iPSCs. Interestingly, the number of gliogenic neurospheres from the (+)/(+) iPSCs were rapidly increased during the neurosphere passages, suggesting that early neurogenic NSCs in the (+)/(+) neurospheres could develop into gliogenic mature NSCs more rapidly and efficiently than the NSCs in the (−)/(+) neurospheres. Next, we evaluated the response to growth factors in NSCs derived from the (−)/(+) and (+)/(+) iPSCs. The secondary spheres derived from the (−)/(+) and (+)/(+) iPSCs were dissociated to form tertiary spheres in the presence of LIF plus FGF2 or FGF2 alone. The neurospheres that appeared in the presence of LIF and FGF2 were considered to reflect the total number of NSCs in the dissociated cells, while neurospheres that appeared in the presence of FGF2 alone reflected the number of FGF2-dependent mature definitive NSCs (Akamatsu et al., 2009). While both the (−)/(+) and (+)/(+) NSCs formed a similar number of tertiary neurospheres in the presence of both LIF and FGF2, the (+)/(+) NSCs formed a significantly increased number of tertiary neurospheres compared with the (−)/(+) NSCs in the absence of LIF (Figure 2D). These data suggested that the (+)/(+) iPSCs developed into definitive NSCs more rapidly than the (−)/(+) iPSCs in terms of responsiveness to growth factors.

To evaluate the development of (−)/(+) and (+)/(+) NSCs per gene expression, we used qRT-PCR to measure the relative mRNA expression levels of neural progenitor markers (*Nestin* and *Pax6*) in the primary, secondary, and tertiary neurospheres. No significant difference was seen in the expression of *Nestin* and *Pax6* between primary neurospheres derived from the (−)/(+) and (+)/(+) iPSCs, while significant differences were observed in the secondary and tertiary neurospheres (Figures 2E and 2F). These results suggested that cells in the neurospheres derived from the (+)/(+) iPSCs differentiated more rapidly and efficiently than those from the (−)/(+) iPSCs.

Previous reports demonstrated that each ESC or iPSC clone had a differentiation propensity to differentiate into a certain cell lineage (Kim et al., 2010a, Osafune et al., 2008). To exclude the possibility that the differentiation propensity of the (+)/(+) iPSCs was simply biased toward the neural lineage, we evaluated the differentiation potentials of the (−)/(+) and (+)/(+) iPSCs toward the mesodermal and endodermal lineage. The (−)/(+) or (+)/(+) iPSCs were separately dissociated in suspension culture without 3i and LIF to form embryoid bodies (EBs) (Figure S3). Then, mesodermal and endodermal gene expression on day 4 and day 8 EBs was measured by qRT-PCR. In the day 8 EBs derived from the (+)/(+) iPSCs, the expression levels of early mesodermal (*Isl1* and *FoxC1*) and endodermal (*FoxA2*, *Sox17*, and *Flk-1*) markers were higher compared with the levels in the (−)/(+) iPSCs (Figure 2G). These results suggested that the differentiation potential of the (+)/(+) iPSCs was not biased toward the neural lineage and that the (+)/(+) iPSCs could also efficiently differentiate into other cell lineages (mesoderm and endoderm) compared with the (−)/(+) iPSCs. Given these observations, we hypothesized that the (+)/(+) iPSCs tended to differentiate from the pluripotent stage to terminally differentiated cells and could not maintain themselves as pluripotent without 3i. Therefore, we removed 3i from the medium to determine whether (+)/(+) iPSCs remained pluripotent without 3i. Interestingly, the (+)/(+) iPSCs lost expression of *Nanog* and *Oct3/4* after the 3i withdrawal, while expression of these genes was significantly maintained in the (−)/(+) iPSCs without 3i (Figures 2H and S4). These results suggested that the pluripotency of the (+)/(+) iPSCs could not be maintained in the absence of 3i.

Although the (−)/(+) and (+)/(+) iPSCs exhibited similar characteristics during the pluripotent stage in the presence of 3i (and LIF), these differentiation potentials (e.g., removal of 3i and glial differentiation) were apparently different even though they were derived from the same fibroblasts.

### Increased Expression of 2-Cell Stage-Specific Genes in iPSCs Established with 3i during the Reprogramming Period

**Figure 3:**
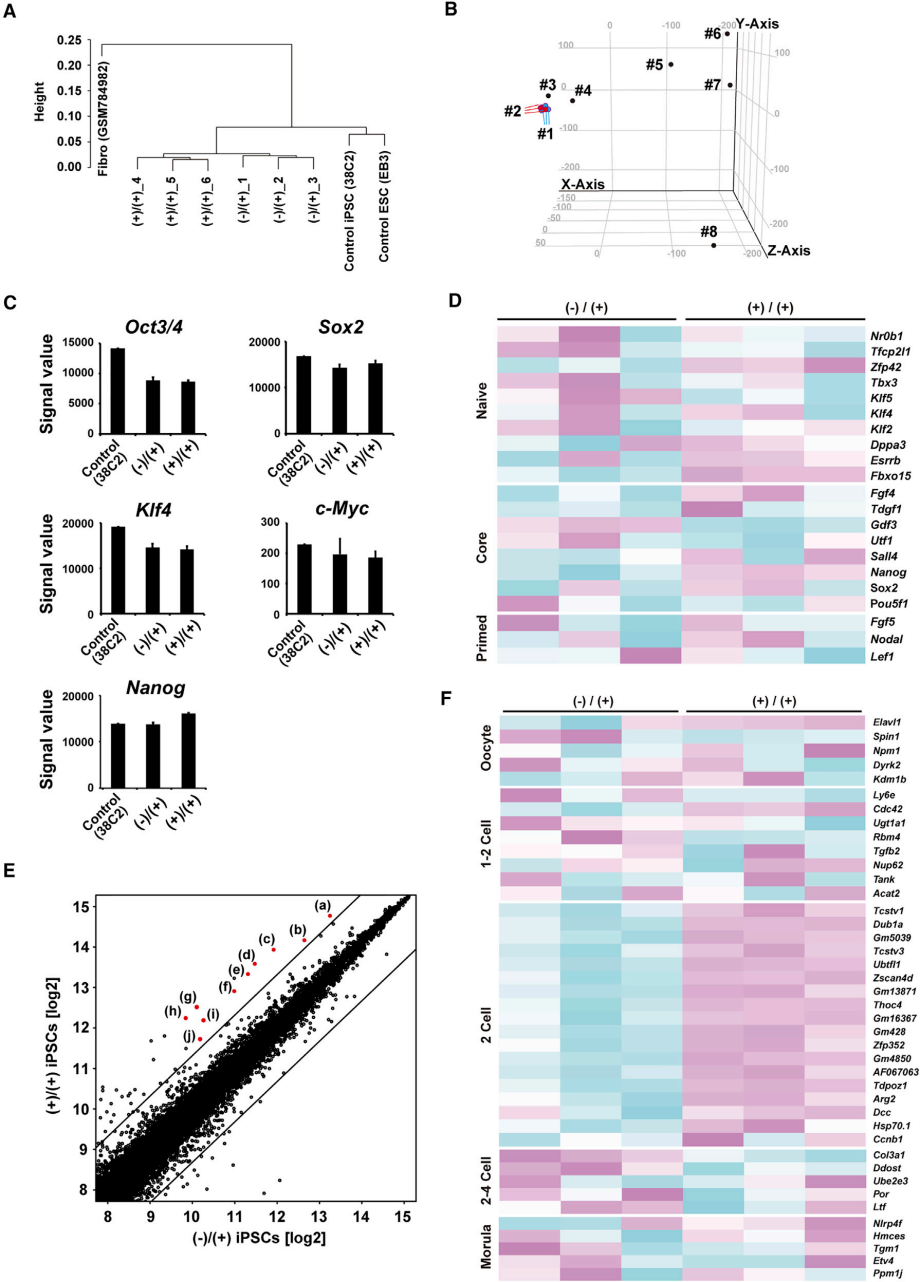
The Differences in Global Gene Expression between (−)/(+) and (+)/(+) iPSCs (A) Hierarchical cluster analysis based on global gene expression of indicated samples. Microarray data of this study and our previous study (GEO: GSE31725) were collectively normalized using the MAS 5.0 algorithm in every analysis. (B) Principal-component analysis of gene expression data. Samples are as follows: nos. 1 and 3, clones of the (−)/(+) iPSCs; nos. 2 and 3, clones of the (+)/(+) iPSCs; no. 3, the clone of the ESCs; no. 4, the clone of conventional iPSC derived from a *Nanog-GFP* mouse; no. 5, the clone of the ESC-derived neurospheres; no. 6, the clone of the iPSC-derived neurospheres; no. 7, the clone of the E14 embryo ganglionic eminence-derived neurospheres; no. 8, the clone of the fibroblasts. Gene expression data for nos. 3–8 clones obtained in our previous study (GEO: GSE31725) were used for comparison. (C) Expression of pluripotent markers. Signal intensities were determined by microarray analysis (n = 3). (D) Heatmap showing the expression levels for genes that are associated with naive, core, and primed pluripotency. (E) Scatterplots comparing global gene expression patterns between (−)/(+) and (+)/(+) iPSCs. Highly expressed 2-cell embryo- or *Zscan4*-related genes are shown as red dots. (a) *Zscan4*, (b) *Eif1a/Eif1a-like*, (c) *Gm13040*, (d) AF067061, (e) *Dub1*, (f) *Gm8300*, (g) *LOC639910 Gm13871*, (h) *Tcstv1*, (i) *Gm428*, (j) *Gm18371*, and (k) *Gm428*. (F) Heatmap showing the expression levels of genes that are specifically expressed in early embryonic stages. Error bars represent mean ± SD from three independent experiments (n = 3).

To explore the difference in global gene expression between the (−)/(+) and (+)/(+) iPSCs that contributed to these distinct differentiation potentials, we performed a microarray analysis using undifferentiated (−)/(+) and (+)/(+) iPSCs along with fibroblasts, ESCs (EB3), and iPSCs (38C2) maintained without 3i as control samples. Hierarchical clustering analysis showed that three independent clones of the (−)/(+) and (+)/(+) iPSCs were clustered into two distinct groups, but they highly resembled each other compared with the fibroblasts or conventional ESCs/iPSCs (Figure 3A). To exclude the possibility that undifferentiated (+)/(+) iPSCs with fast and efficient neural differentiation potentials could be clustered closer to NSCs than (−)/(+) iPSCs in the global gene expression pattern, we performed principal-component analysis (PCA) by using the above samples alongside neurospheres derived from the E14.5 murine embryonic striatum, neurospheres derived from conventional ESCs, and neurospheres derived from conventional iPSCs. PCA showed a similar tendency to that observed in the hierarchical clustering analysis and revealed that the (+)/(+) iPSCs were located much closer to the (−)/(+) iPSCs or conventional ESCs/iPSCs than to the neurospheres (Figure 3B). Then, we concluded that the (+)/(+) iPSCs were in an undifferentiated state with a fast and efficient neural differentiation potential and not biased to a neurally differentiated state like the NSCs.

Next, we compared gene expression between the (−)/(+) (clone 1–3) and (+)/(+) (clone 4–6) iPSCs to explore the key factor driving the difference in their differentiation potentials. As expected, the expression levels of the representative core, naive, and primed pluripotent marker genes were similar between the two groups (Figures 3C and 3D). Gene ontology analysis highlighted biological functions for the differentially expressed genes (DEGs) (Table S3), and scatterplots revealed DEGs (>2.5-fold change, t test, p < 0.05, average signal value >500; Figure 3E). Interestingly, 12 highly expressed DEGs (>2.5-fold change, t test, p < 0.05, signal value [log2] >9) contained 10 genes that were specifically related to 2-cell embryos, including *Zscan4*, *Eif1a/Eif1a-like*, *Dub1*, and *Gm428* (Amano et al., 2013, Dan et al., 2013). Only two genes (*BC080696* and AA645497) were not 2-cell-related genes, which are not annotated genes. We analyzed representative genes expressed in every early developmental stage, and 2-cell genes were specifically expressed (Figure 3F). These data suggest that the (+)/(+) iPSCs are similar to 2-cell stage cells in global gene expression compared with the (−)/(+) iPSCs.

### Differentiation Potential of (+)/(+) iPSCs during the Reprogramming Period with the 3i Chemicals

**Figure 4:**
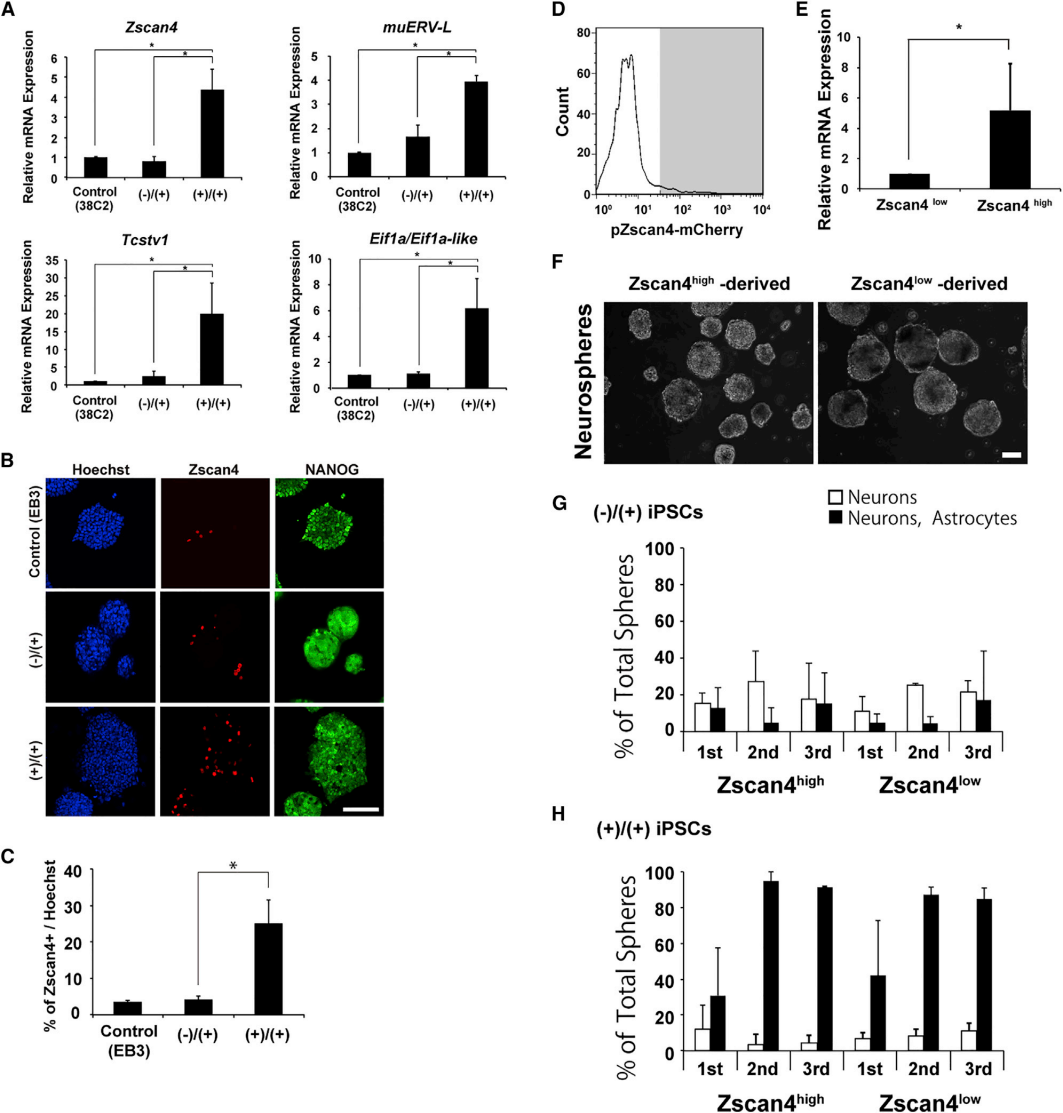
Expression of Genes Associated with the 2-Cell Stage in (−)/(+) and (+)/(+) iPSCs (A) qRT-PCR analysis of 2-cell-related genes in (−)/(+) and (+)/(+) iPSCs (n = 3; ^∗^p < 0.05). 38C2 represents conventional iPSCs. (B) Representative immunocytochemistry images of iPSCs. Nanog expression is indicated by the GFP fluorescence in (−)/(+) and (+)/(+) iPSCs, and the Nanog protein in ESCs is immunostained. Scale bar, 100 μm. (C) Quantitative analysis of Zscan4-positive cells in (−)/(+) and (+)/(+) iPSCs (n = 3; ^∗^p < 0.05). ESCs were used as a control. (D) Zscan4 high or low cells were sorted using a BD FACSAria III cell sorter. (E) Expression of *Zscan4* in sorted cells was verified by qRT-PCR (n = 3; ^∗^p < 0.05). (F) Representative images of Zscan4^high^ iPSC- and Zscan4^low^ iPSC-derived neurospheres. Scale bar, 100 μm. (G and H) Differentiation efficiency of the first, second, and third neurospheres derived from Zscan4^high^ and Zscan4^low^ iPSCs. The results for the (−)/(+) iPSCs (G) and (+)/(+) iPSCs (H) are shown. The frequency of colonies consisting of neurons (βΙΙΙ-tubulin) and/or astrocytes (GFAP) was evaluated by immunocytochemistry and is presented as the percentage of positive colonies (n = 3).

Based on the results of the microarray analysis, which showed that the (+)/(+) iPSCs had higher 2-cell stage gene expression than the (−)/(+) iPSCs, we performed detailed examinations of the expression of 2-cell stage genes in the (+)/(+) iPSCs by qRT-PCR and immunocytochemistry. The expression levels of the 2-cell stage genes (*Zscan4*, *muERV-L*, *Tcstv1*, and *Eif1a/Eif1a-like*) were significantly increased in the (+)/(+) iPSCs compared with the (−)/(+) iPSCs or control iPSCs (Figure 4A). An increased number of Zscan4-positive cells in the (+)/(+) iPSC group was confirmed by immunocytochemistry (Figures 4B, 4C, and S5). These findings suggested that addition of 3i during the reprogramming period, not the maintenance period, enhanced the gene expression pattern of 2-cell stage iPSCs at the mRNA and protein levels.

It has been reported that a small fraction of cells that express 2-cell stage genes exist among undifferentiated pluripotent cells (Falco et al., 2007, Macfarlan et al., 2012), even in conventional culture conditions. We next sought to determine whether these 2-cell stage gene-positive cells in the (−)/(+) PSC group were identical to the rapidly differentiating cells observed in the (+)/(+) iPSC group. When both populations were identical, the 3i chemicals during the reprogramming period simply increased the 2-cell stage gene-positive cells in the (+)/(+) iPSC population.

To characterize the cells with high 2-cell stage gene expression, we isolated these cells using the pZscan4-mCherry reporter, as a previous report showed that Zscan4-positive cells highly expressed other 2-cell genes and showed similar oscillations in the expression of these genes (Amano et al., 2013, Zalzman et al., 2010). A plasmid containing the pZscan4-mCherry reporter and a PGK-Neomycin resistance gene was transiently transfected into (−)/(+) and (+)/(+) iPSCs; these cells were selected with G418 and sorted into Zscan4-mCherry-high (Zscan4^high^) or *Zscan4-*mCherry-low (Zscan4^low^) populations by fluorescence-activated cell sorting (Figure 4D). qRT-PCR analysis revealed that the Zscan4^high^ cells expressed approximately 5-fold more *Zscan4* that the Zscan4^low^ cells (Figure 4E). Then, we generated neurospheres using Zscan4^high^ or Zscan4^low^ cells from the (−)/(+) iPSCs. The frequencies of gliogenic sphere formation from mature NSCs were relatively low and were similar between the Zscan4^high^ and Zscan4^low^ cells, while the Zscan4^low^ cells from the (+)/(+) iPSCs mainly give rise to mature gliogenic neurospheres, as did the Zscan4^high^ cells from the (+)/(+) iPSCs (Figures 4F–4H). These data suggested that Zscan4^high^ cells were present in (−)/(+) iPSC group but that the Zscan4^high^ cells were distinct from those of the (+)/(+) iPSCs. Presumably, this difference was already determined during the reprogramming period with 3i chemicals. An increased number of Zscan4^high^ cells in the (+)/(+) iPSC group suggested that the (+)/(+) iPSCs were stable at a distinct status, where 2-cell stage markers were expressed, compared with the (−)/(+) iPSCs.

## Discussion

In this study, we showed that exposure to 3i chemicals during a reprogramming period altered the differentiation potentials of generated iPSCs and that these potentials could not be changed by 3i exposure once reprogramming was completed. It remains controversial whether these chemicals, including FGF-MAPK cascade/GSK3β inhibitors, which induce ground-state pluripotency in PSCs, can improve the differentiation potentials of PSCs (Chan et al., 2013, Gafni et al., 2013, Takashima et al., 2014, Theunissen et al., 2014, Valamehr et al., 2014). Although many chemical compounds have been reported for facilitating reprogramming or sustaining pluripotency (Huangfu et al., 2008, Li et al., 2010, Mali et al., 2010, Silva et al., 2008, Zhu et al., 2010), it is not fully known whether iPSCs generated with these chemicals had similar differentiation potentials compared with conventional iPSCs in both mice and humans.

Before the 3i chemicals were discovered to maintain PSC pluripotency (Ying et al., 2008), ESCs with chimera-forming ability had been established only in mice (Evans and Kaufman, 1981) because it was very difficult to maintain the pluripotent states of cells from other species. However, using 3i, ESCs have been established from rats (Buehr et al., 2008), indicating that these ESCs could not be maintained without the support of 3i. Even in the murine cells that we have analyzed in this study, the number of iPSC colonies was significantly increased with 3i during the reprogramming period. Therefore, it is likely that cells that cannot be reprogrammed and maintained in pluripotency without 3i chemicals correspond to the increased population in 3i-treated reprogramming. Our data showing that (+)/(+) iPSCs quickly lost their pluripotency without 3i (Figure S4) might support this hypothesis.

We previously reported that fibroblasts can be directly converted into NSCs through partially reprogrammed intermediates by the brief expression of Yamanaka factors. Furthermore, we reported that these induced NSCs (diNSCs) are highly gliogenic, even at an early passage, whereas ESC-derived neurospheres generate mostly neurons and a few astrocytes. These observations suggest that diNSCs can develop into mature NSCs more rapidly along with NSCs derived from mouse brains (Matsui et al., 2012). It has also been reported that hepatocytes derived from partially reprogrammed intermediate cells mature more efficiently than those derived from iPSCs (Zhu et al., 2014). These observations also suggested that bypassing the iPSC state can induce rapidly differentiating cells because clonal isolation of iPSCs may result in loss of these cells due to long-term exposure to the severe selective culture condition.

Here, we have clearly shown that there are distinct differentiation/maturation potentials between (+)/(+) and (−)/(+) iPSCs that can be determined by the culture condition during a reprogramming period, not during a maintenance period. Another study that examined early differentiation using EB formation has shown that the differentiation potential of iPSCs can be changed by SB431542, ascorbic acid, thiazovivin, and PD0325901 during the reprogramming period (Park et al., 2015). Interestingly, our results have also shown that differentiation from (+)/(+) iPSCs is accelerated toward all three germ layers in comparison with (−)/(+) iPSCs. Several previous reports have shown that epigenetic memories influence biased and insufficient differentiation potentials in PSCs (Kim et al., 2010b, Koyanagi-Aoi et al., 2013, Polo et al., 2010). Moreover, FGF4-MAPK/GSK3β inhibitors affect the global methylation profile (Leitch et al., 2013) in PSCs, and it has been suggested that treatment with 3i during a reprogramming period might induce irreversible epigenetic changes in iPSCs.

We found that the expression of 2-cell stage genes including *Zscan4* was significantly different between (+)/(+) and (−)/(+) iPSCs. Although one previous report demonstrated that the addition of 2i to established iPSCs promotes the expression of 2-cell genes (Cerulo et al., 2014), the Zscan4 expression levels in both (−)/(+) iPSCs and control ES/iPSCs were similar in this study. Our data also showed that the amount of Zscan4 in each iPSC did not affect the iPSC differentiation potential; these characteristics had already been determined during the reprogramming period. This finding seems to be compatible with the previous report mentioning that forced expression of Zscan4 during reprogramming improved differentiation potentials of iPSCs as evaluated by the tetraploid complementation assay (Jiang et al., 2013). These 2-cell stage genes are not the markers that predict the rapid differentiation potential of each iPSC. Identification of these preventative markers will be useful for isolating rapidly differentiating iPSCs from conventional iPSC clones. However, it is significant that (+)/(+) iPSCs are stable at a distinct stage with higher expression levels of 2-cell stage genes compared with (−)/(+) iPSCs. However, DNA were hypermethylated in (+)/(+) iPSCs. In the development of the early embryo, the total amount of genomic methylation is gradually decreased toward the blastocyst stage (Wu and Zhang, 2014). These data suggested that the (+)/(+) iPSCs, which are hypermethylated, are at a younger stage compared with (−)/(+) iPSCs. Transient histone acetylation in heterochromatin as well as DNA demethylation have reported to occur during the activation of 2-cell stage genes in ESCs (Akiyama et al., 2015), although it remains unclear whether these epigenetic modifications influence accelerated differentiation potentials of (+)/(+) iPSCs.

In summary, our study demonstrated that the use of 3i chemicals—PD184352, CHIR99021 and SU5402—during a reprogramming period induces accelerated differentiation/maturation potentials in murine iPSCs that are stable at a distinct state with increased expression of 2-cell stage markers and highly methylated profile that is likely to be primitive compared with those without 3i chemicals. Whether naive human iPSCs have a superior differentiation ability to primed hiPSCs remains controversial in the field of regenerative medicine. Our observations may support the hypothesis that PSCs that are stable at a distinct stage have an accelerated and non-biased differentiation potential compared with those at a lower stage.

## Experimental Procedures

The teratoma assay was performed in accordance with the Guidelines for the Care and Use of Laboratory Animals of Keio University (assurance no. 09169). Chimera formation was performed in accordance with the Guidelines for the Care and Use of Laboratory Animals of Juntendo University (assurance no. 300083).

### iPSC Generation

Murine iPSC generation was performed as described previously with slight modifications. In brief, 3.6 × 10^6^ Plat-E packaging cells were seeded per 100-mm dish, and, the next day, these cells were transfected with pMXs retroviral vectors (encoding *Oct 3/4*, *Klf4*, *Sox2*, and *c-Myc*; Addgene) using the Fugene 6 transfection reagent (Roche). At this time, we also transfected dsRed to ascertain silencing of the transgenes after colony formation. Virus-containing medium supplemented with 4 μg/mL Polybrene was added to 8 × 10^5^ MEFs, and MEFs were re-seeded on SNL feeder cells 4 days after infection. Approximately 3 weeks after the transduction, iPSC colonies were picked and expanded. (+)/(+) iPSCs were treated with 3i beginning 2 days after the transduction, while (−)/(+) iPSCs were treated with 3i after colony isolation (approximately 3 weeks after the transduction).

### Quantification of Reprogramming Efficiency

For quantification of the reprogramming efficiency of murine cells, 1 × 10^5^ transduced MEFs were re-seeded into six-well plates (Nunc). Twenty-one days after the transduction, the number of *Nanog*-EGFP^+^ and dsRed^−^ ESC-like colonies was counted.

### *In Vitro* Neural Differentiation of iPSCs

iPSCs were dissociated with TrypLE Select (Life Technologies) and suspended at a density of 1 × 10^5^ cells/mL in a serum-free media hormone mix (MHM) medium supplemented with B27 (Life Technologies), 1,000 U/mL LIF (Nakalai Tesque) and 20 ng/mL FGF2 (PeproTech) to form neurospheres. Neurospheres were passaged repeatedly every 5–7 days by enzymatic dissociation into single cells in the same culture medium. For sphere formation assays, neurospheres were dissociated and plated at 1 × 10^4^ cells/mL in 96-well ultra-low plates, and the number of formed spheres per well was counted after 7 days. For neural differentiation assays, neurospheres were seeded on poly-L-ornithine and fibronectin-coated chambers and cultured for 7 days in MHM medium supplemented with B27 and 1% FBS.

### Global Gene Expression Analysis

Total RNA purification was performed using TRIzol reagent and a RNeasy Mini Kit (QIAGEN). Global gene expression analysis was performed using Affymetrix Gene Chip Mouse Genome 430 2.0 as per the manufacturer's protocol. The microarray signals were quantified using the MAS 5.0 method. Hierarchical clustering and heatmaps were designed using the R package and Bioconductor. Distances (=1 – Spearman's correlation coefficient) were calculated using the R package. PCA and scatterplots were designed using GeneSpring GX. Gene ontology analysis was performed through the use of QIAGEN's Ingenuity Pathway Analysis (QIAGEN Redwood City, www.qiagen.com/ingenuity).

## Author Contributions

K.N., T. Shiga, H.O., and W.A. designed the experiments, analyzed the data, and wrote the manuscript. K.N. and T. Shiga performed most of the experiments and analyzed the data. T.A. and M.S.H.K. designed and made the reporter construct. S.S. performed fluorescence-activated cell sorting analysis. E.N. and N.T performed chimera formation experiments. T. Sasaki analyzed MBD-seq data. All the authors read and approved the final version of the manuscript. H.O. is a founding scientist and a paid member of the Scientific Advisory Board of San Bio and K Pharma. All other authors declare that they have no competing interests.

## Accession Numbers

The accession number for the microarray data in this paper is NCBI GEO: GSE110515. The MBD-seq data reported are available in the DDBJ databases under the accession number DRA007928.

## Supplemental Information

Document S1. Supplemental Experimental Procedures, Figures S1–S5, and Tables S1–S5

Document S2. Article plus Supplemental Information
