# Supplementary material for: Induced Pluripotent Stem Cells Reprogrammed with Three Inhibitors Show Accelerated Differentiation Potentials with High Levels of 2-Cell Stage Marker Expression
Source: Stem Cell Reports. 2019 Jan 31;12(2):305–18. doi: 10.1016/j.stemcr.2018.12.018 (PMC6373546; doi:10.1016/j.stemcr.2018.12.018)
Supplement: Document S1. Supplemental Experimental Procedures, Figures S1–S5, and Tables S1–S5 [file mmc1.pdf]

Stem Cell Reports, Volume *12*

Supplemental Information

Induced Pluripotent Stem Cells Reprogrammed with Three Inhibitors Show Accelerated Differentiation Potentials with High Levels of 2-Cell Stage Marker Expression

Koji Nishihara, Takahiro Shiga, Eri Nakamura, Tomohiko Akiyama, Takashi Sasaki, Sadafumi Suzuki, Minoru S.H. Ko, Norihiro Tada, Hideyuki Okano, and Wado Akamatsu

Supplemental Experimental Procedures

Cell culture

Mouse embryonic fibroblasts (MEFs) were derived from E13.5 embryos from homozygous *Nanog*-EGFP transgenic mice (a kind gift from Dr. Okita (and Dr. Yamanaka) of Kyoto University). MEFs were cultured in Dulbecco's modified Eagle's medium (DMEM) supplemented with 10% fetal bovine serum (FBS), L-glutamine, penicillin, and streptomycin. We used MEFs to generate iPSCs between passages 3 and 5. Established iPSCs were cultured in mouse ES cell medium (DMEM containing 15% FBS, 1,000 U/ml of leukemia inhibitory factor (LIF) (Nakalai Tesque), a nonessential amino acid solution, L-glutamine, β-mercaptoethanol, penicillin and streptomycin) on mitomycin-C-treated SNL feeder cells and treated with or without small molecule inhibitors known as 3i (0.8 μM MEK inhibitor PD184352, 2 μM FGF-R inhibitor SU5402 and 3 μM GSK-3β inhibitor CHIR99021). All experiments were conducted at passages 6-18. A previously established murine iPSC clone (38C2) generated from a *Nanog*-GFP mouse was also used as a control iPSCs.

Immunocytochemistry and Alkaline Phosphatase (AP) staining

For immunocytochemical analysis, cells were fixed with 4% paraformaldehyde (PFA) in phosphate buffered saline (PBS) for 25 min. The cells were analyzed by immunofluorescence staining using antibodies to the following proteins: Nanog (1:100, ReproCELL), SSEA-1 (500:1, Abcam), β III-tubulin (1:1000, Sigma), GFAP (1:4000, DAKO). Cells were washed with PBS three times, and incubated with an Alexa Fluor 488- or 555-conjugated secondary antibody (1:500, invitrogen). Nuclei were stained with 10 μg ml-1 Hoechst 33258 (Sigma). Images were acquired with Apotome (Zeiss) or LSM-710 confocal (Zeiss) microscopes. AP staining was performed using the Leukocyte Alkaline Phosphatase Kit (Sigma) following the manufacturer's instruction.

Teratoma Assay

To assess teratoma formation, iPSCs were injected into the testis of 7-week-old SCID mice (Charles River Laboratories). Four weeks after transplantation, tumors were dissected and fixed with 4% PFA in PBS. Paraffin-embedded tissue was sectioned and stained with hematoxylin and eosin (H&E) staining. Images were obtained using an Axio Imager M1 microscope (Zeiss). Teratoma Assay were performed in accordance with the Guidelines for the Care and Use of Laboratory Animals of Keio University (Assurance no. 09169).

RNA Isolation, Reverse Transcription, and Polymerase Chain Reaction (PCR)

Total RNA was purified using TRIzol Reagent (Invitrogen) and RNeasy Kit (Qiagen) according to the manufacturer's protocol with slight modifications. First-strand cDNA was synthesized with the SuperScript III First-strand Synthesis System (Invitrogen).

Quantitative real-time PCR reactions were performed using SYBR Premix Ex Taq II (Takara), and transcript levels were determined using a ABI PRISM Sequence detection System 7900HT or ViiA7 (Applied BioSystems). The amount of mRNA was normalized to β-actin mRNA. The primer sequences used in this study are listed in Table S3.

Fluorescence-Activated Cell Sorting (FACS)

We performed fluorescence-activated cell sorting (FACS) to sort iPSCs using a BD FACSAriaIII cell sorter. iPSCs and feeder cells were dissociated and seeded onto gelatin-coated dishes for 20 - 25 min to exclude feeder cells, and the supernatant was harvested and suspended in a diluted mixture of mouse ES medium and PBS. EB3s (ESCs with no fluorescence) and EB3s transfected with a CAG-RFP reporter were used as control cells.

MBD-sequencing (MBD-seq)

iPSCs were purified with FACS via size gating and fluorescence (*Nanog*-EGFP) to remove feeder fibroblasts. Genomic DNA was sonicated to produce fragments, and methylated DNA was captured with an EpiXplore Methylated DNA Enrichment Kit (Takara) according to the manufacturer's protocol. Sequencing libraries were prepared by using a TruSeq ChIP Sample Prep Kit (Illumina). DNA fragments in the MBD-seq library were sequenced by Illumina GAIIx (Illumina) in single-end 75-bp mode. The obtained reads were mapped to mouse reference sequence (mm9) by the BWA program (version 0.6.1), and the mapped data were analyzed by the MACS program in Avadis NGS 1.3.1 (Strand Life Sciences) to detect MBD binding regions. The gene body including 3 kb upstream of the translational start site was treated as the gene region.

CGH array

CGH array was performed according to the Agilent CGH array protocol (v7.5). Genomic DNA was extracted from iPSCs and MEFs using a Qiagen DNeasy Blood and Tissue kit. The array was measured using a SurePrint G3 mouse CGH 4x80K array (TAKARA BIO, Japan).

Chimera formation

Oocytes were collected from B6.albino female mice (Charles River Laboratories Japan, Kanagawa, Japan)

superovulated by intraperitoneal injection of PMSG (Peamex, Sankyo Lifetech Inc., Tokyo, Japan) followed by hCG (Sigma-Aldrich, St. Louis, Missouri, USA) at an interval of 48h, and then fertilized in vitro with spermatozoa from B6.albino male mice (Charles River Laboratories). Mouse blastocysts were obtained from oocytes fertilized in vitro by culturing them for 3.5 days after fertilization. Mouse iPSCs were trypsinized and directly injected 10~15 cells into the cavity of blastocysts. Injections were performed by micromanipulators (Leica, Wetzlar, Germany) with a PMM-150 FU piezo-impact drive unit (Prime Tech Inc., Ibaragi, Japan) using a blunt-ended, mercury-containing injection pipette. Injected blastocysts were transferred to the uterus of a 2.5-dpc pseudopregnant ICR recipient females (Charles River Laboratories Japan) that were mated to vasectomized ICR male mice (Charles River Laboratories Japan). Chimera formation were performed in accordance with the Guidelines for the Care and Use of Laboratory Animals of Juntendo University (Assurance no. 300083).

Statistical Analysis

Data shown are represented as means ± SEM. Student’s t test was used for comparisons of two groups. p Values <0.05 were considered significant.

SUPPLEMENTAL REFERENCES

Fujimori K, Matsumoto T, Kisa F, Hattori N, Okano H, Akamatsu W. (2017). Escape from Pluripotency via Inhibition of TGF-β/BMP and Activation of Wnt Signaling Accelerates Differentiation and Aging in hPSC Progeny Cells. Stem Cell Reports *14*, 1675-1691.

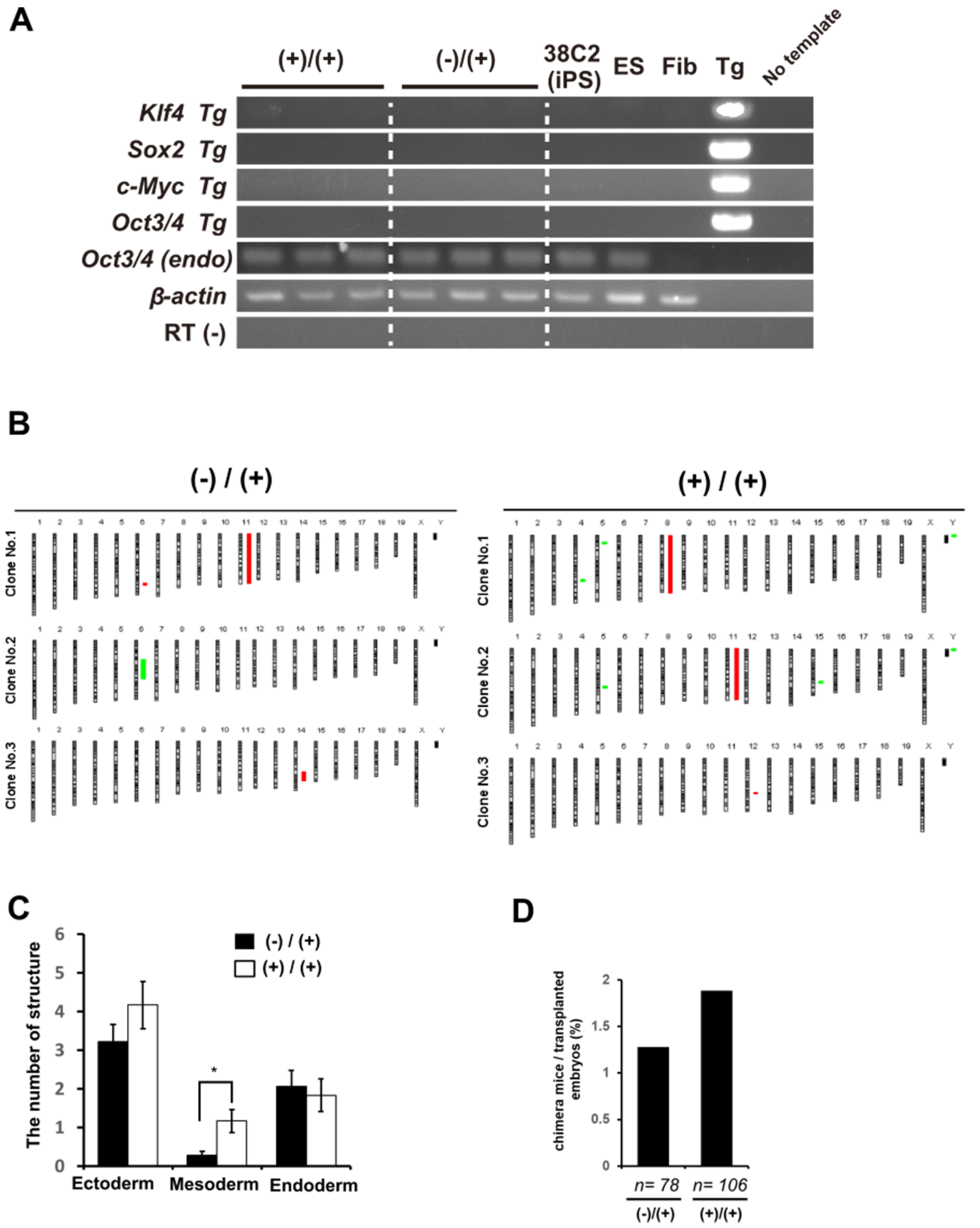

Figure S1. Expression patterns of transgenes in (-)/(+) and (+)/(+) iPSCs (A) Total RNA was isolated from each cell type, and RT-PCR analysis was performed using primers amplifying the transgene transcripts (*Klf4, Sox2, c-Myc, and Oct3/4 Tg*), the coding region of endogenous *Oct3/4* (endo) and *β-actin* (as loading control). Control templates were as follows: 38C2, previously established iPSCs derived from *Nanog*-GFP mouse; ES, EB3 mouse ESCs; Fib, fibroblasts; Tg; pMXs, retroviral vectors of each transgene.(B) Summary of genetic alterations in iPS cell lines based on CGH array. Chromosomal

alteration frequencies are visualized by red lines (amplifications), and green lines represent deletions. (C) Teratomas generated from (-)/(+) and (+)/(+) iPSCs injected into NOD/SCID mice were stained with hematoxylin and eosin. The numbers of differentiated structures from three germ layers (ectoderm: neural epithelium and neural rosettes; mesoderm: cartilage and bone; endoderm: gut-like epithelium with Goblet cells or ciliated structures) were counted from randomly selected 10x visual fields (3 clones of (-)/(+) and (+)/(+) iPSCs, n=6; *, $p < 0.05$).

(D) Graph shows percentage of chimeric mouse at 4 weeks of age in (-)/(+) and (+)/(+) iPSCs . n indicates the number of embryos transplanted.2n blastocyst injections. Numbers of animals obtained per total number of transferred embryos are shown.

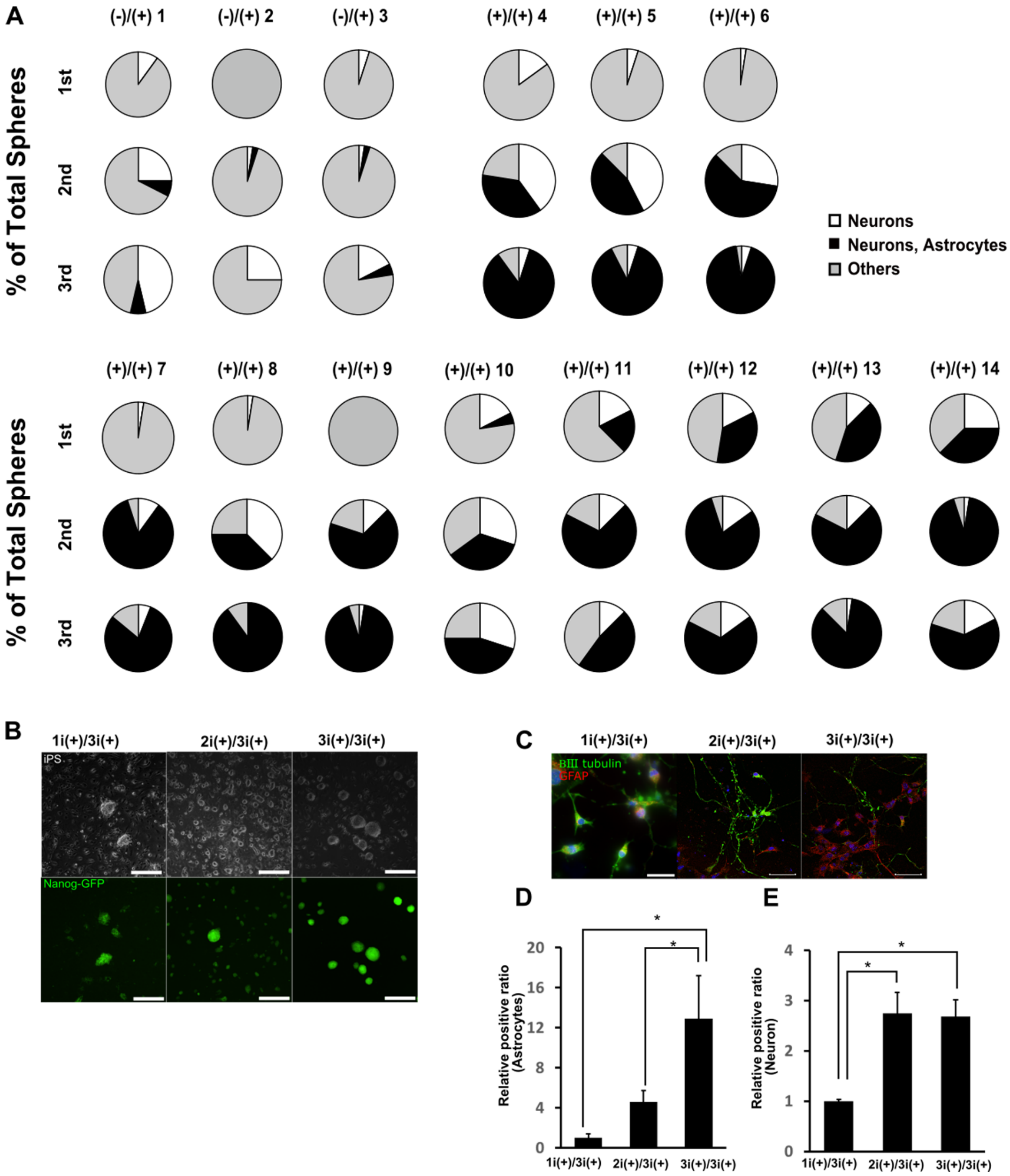

Figure S2. Differentiation of patterns in 3i, 2i and 1i- reprograming iPSCs

(A) Differentiation efficiency of the 1st, 2nd and 3rd neurospheres derived from (-)/(+) (clone 1-3) and (+)/(+) (clone 4-14) iPSCs. The frequency of colonies consisting of neurons (βⅢ-tubulin) and/or astrocytes (GFAP) was evaluated by immunocytochemistry and is presented as the percentage of positive colonies. (B) Morphology and *Nanog*-GFP expression of undifferentiated iPSC colonies reprogrammed with Cyto-Tune-iPS (MBL) in 1i (SU5402), 2i (PD184352 and CHIR99021) and 3i. Scale bar: 200 μm. (C) Differentiated neurons and astrocytes were detected by **βIII-tubulin** (green) and **GFAP** (red)

antibodies. Scale bar: 50μm. (D, E) The differentiation efficiency into astrocytes (D) and neurons (E) from 1i, 2i or 3i reprogrammed iPSCs (1i: n=6, 2i: n=9, 3i: n=8; *, $p < 0.05$). Immunostained cells were imaged and analyzed using an IN Cell Analyzer 2200 high contents cellular analysis system (GE Healthcare) as described previously (Fujimori et al., 2017).

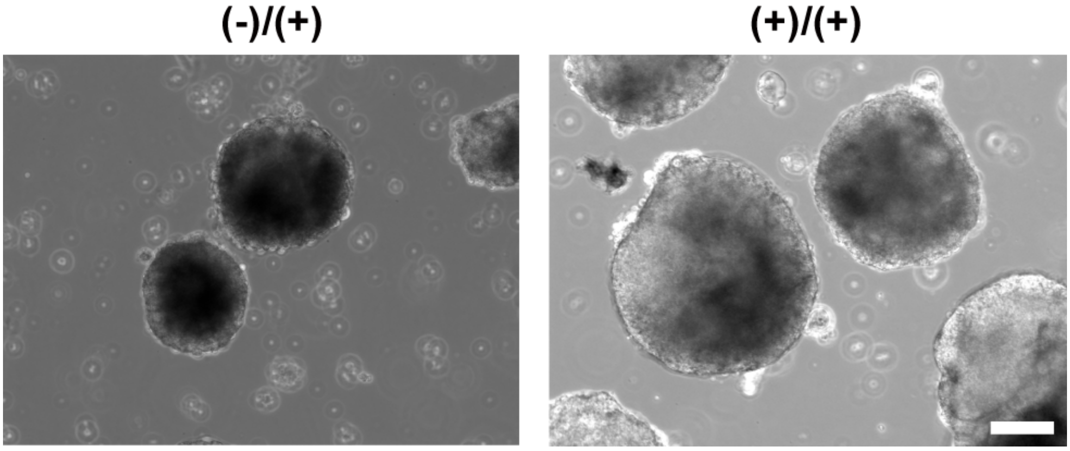

Figure S3. Representative morphology of EBs derived from (-)/(+) and (+)/(+) iPSC cell lines. Scale bar: 100 μm.

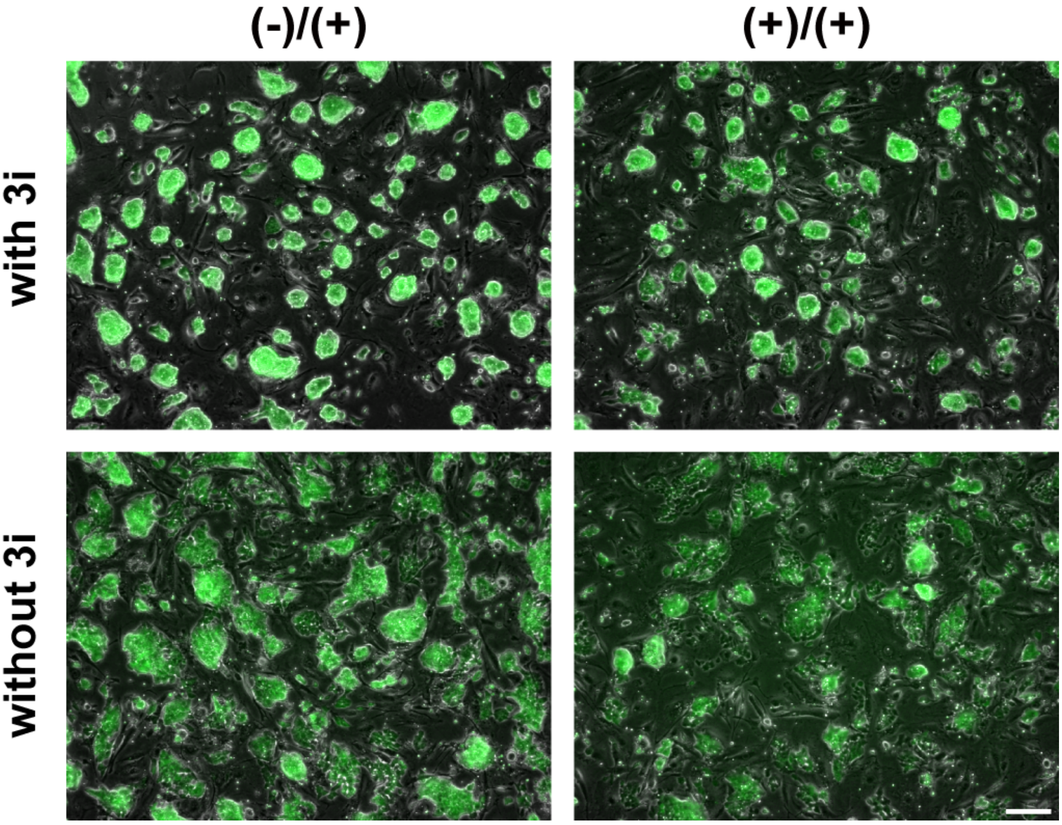

Figure S4. Representative colony morphology of (-)/(+) and (+)/(+) iPSC cell lines before or after withdrawal of 3i chemicals. Scale bar: 200 μm.

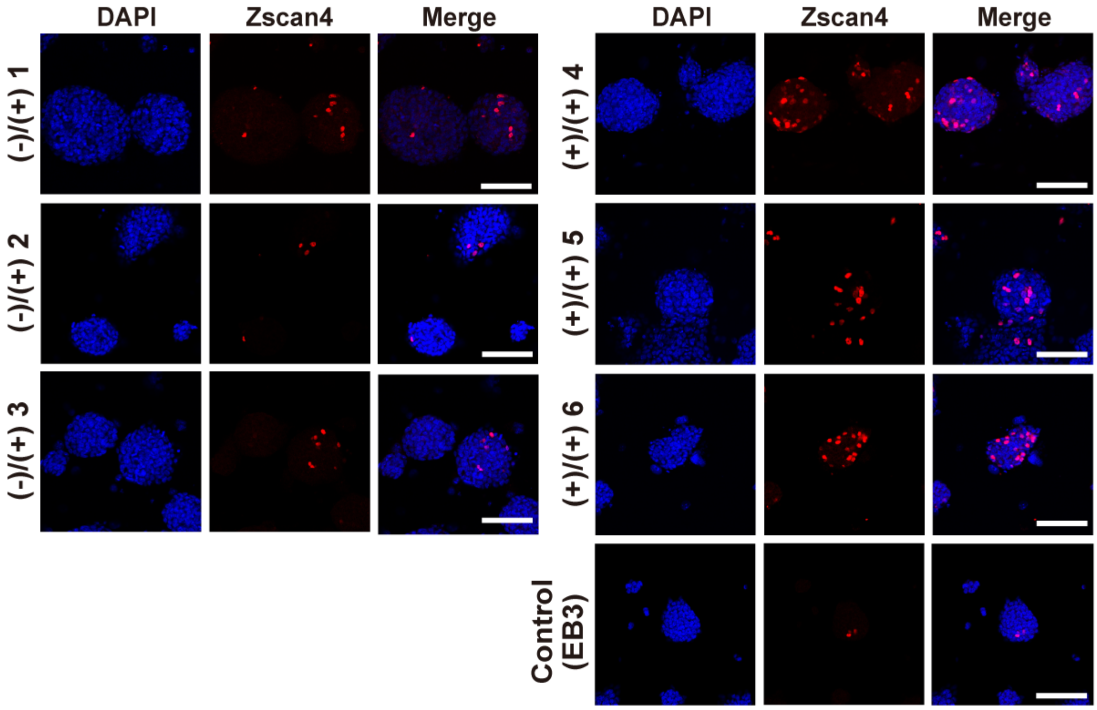

Figure S5. Representative images of immunocytochemistry of Zscan4 in (-)/(+) and (+)/(+) iPSCs (n=3, * ,$p < 0.05$). ESCs were used as controls.

Table S1. Region summary for CGH array

sample No.	Chr	Cytoband	Start	Stop	#Probes	Amplification	Deletion	pval	Gene Names
(-)/(+)_No.1	chr6	qF1 - qF3	120562739	1.25E+08	364	0.620126	0	0	Cecr2, Slc25a18, Atp6v1e1, Bcl2l13, Bid, Mical3, Pex26, Tuba8, Usp18, Slc6a13, Slc6a12, Iqsec3, A2m, Mug1, Cpamd8, Gm10319, Mug-ps1, Klrg1, M6pr, Phc1, Rimklb, Mfap5, Aicda, Apobec1, Gdf3, Dppa3, Nanog, Nanogpd, Slc2a3, Foxj2, C3ar1, Necap1, Clec4a1, Clec4a3, Clec4a4, Clec4b1, Clec4a2, Clec4b2, Clec4n, Clec4d, Clec4e, Vmn2r19, Vmn2r20, Vmn2r21, Vmn2r22, Vmn2r23, Vmn2r24, Vmn2r25, Vmn2r26, Vmn2r27, Cd163, Pex5, Clstn3, C1rl, C1r, C1s, C1rb, Gm5077, Lpcat3, Emg1, Phb2, Ptpn6, Grcc10, Rnu7, Atn1, Eno2, Lrrc23, Spsb2, Tpi1, Usp5, Cdca3, Gnb3, Leprel2, Gpr162, Cd4, Lag3, Ptms, Mlf2, Cops7a, C530028O21Rik, Zfp384, Ing4, Acrbp, Lpar5, Chd4, Nop2, Iffo1, Gapdh, Ncapd2, Mrpl51, Vamp1, Tapbpl, Cd27, 4930417O13Rik, Tuba3a, Ltbr, Scnn1a, Tnfrsf1a, Plekhg6
(-)/(+)_No.1	chr11	qA1 - qE2	3107005	1.22E+08	8984	0.555605	0	0	[illegible]
(-)/(+)_No.2	chr6	qB2.3 - qD3	49542982	96274002	3250	0	-0.81168	0	Npy, Mpp6, Dfna5, Osbpl3, Cycs, 5430402O13Rik, 4921507P07Rik, Npvf, Nfe2l3, Hnrnpa2b1, Cbx3, Snx10, Skap2, Hoxa1, Hoxa2, Hoxa3, Hoxa4, Hoxa5, Hoxa6, Hoxa7, Hoxa9, Hoxa10, Hoxa11, Hoxa11as, Hoxa13, Evx1, Hibadh, Tax1bp1, Jazf1, Creb5, 1200009O22Rik, Cpvl, Chn2, Prr15, Scrn1, Fkbp14, Plekha8, 2410066E13Rik, Znrf2, Nod1, Ggct, Gars, Crhr2, Inmt, Fam188b, Aqp1, Ghrhr, Adcyap1r1, Neurod6, Ccdc129, Gsbs, Pde1c, Lsm5, Avl9, Kbtbd2, Fkbp9, Nt5c3, V1rc21, V1rc19, V1rc20, V1rc32, V1rc30, V1rc1, V1rc3, Gm6674, V1rc5, V1rc7, V1rc6, V1rc29, V1rc16, V1rc26, V1rc27, Gm5569, Ppm1k, Herc5, Pigy, Lancl2, AW146242, V1rc28, V1rc23, V1rc24, V1rc18, V1rc8, V1rc17, V1rc33, V1rc25, V1rc2, V1rc22, Abcg2, Herc3, Nap1l5, Fam13a, Tigd2, Gprin3, Snca, Mmrn1, Grid2, Atoh1, Smarcad1, Hpgds, C130060K24Rik, Tnip3, A930038C07Rik, Prdm5, 4930544G11Rik, Mad2l1, V1rc15, V1rc14, V1rc12, V1rc11, V1rc10, V1rc13, Gng12, Gadd45a, Serbp1, Il12rb2, Il23r, Tacstd2, 4930515G16Rik, Rprl1, Rpia, Eif2ak3, 1700011F03Rik, Foxi3, Thnsl2, Fabp1, Smyd1, Krcc1, Cd8b1, Cd8a, Rmnd5a, Rnf103, Vps24, Kdm3a, Reep1, Mrpl35, Immt, Ptcd3, Polr1a, St3gal5, Atoh8, Sftpb, Usp39, 0610030E20Rik, Tmem150, Rnf181, Vamp5, Vamp8, Ggcx, Mat2a, Capg, Elmod3, Retsat, Tgoln1, Tgoln2, Tcf3, Kcmf1, Tmsb10, Suclg1, 4931417E11Rik, Ctnna2, Lrrtm1, Reg3b, Reg3d, Reg3a, Reg2, Reg1, Reg3g, Lrrtm4, AW146020, Mrpl19, Fam176a, Tacr1, Pole4, Hk2, Sema4f, D6Mm5e, Dok1, Loxl3, Htra2, Aup1, Dqx1, Tlx2, Pcgf1, Lbx2, Ccdc142, Mrpl53, Mogs, Wbp1, Ino80b, Rtkn, Wdr54, 1700003E16Rik, Dctn1, Mthfd2, Mobkl1b, Bola3, Dguok, Actg2, Stambp, Clec4f, Cd207, Vax2os2, Vax2os1, Vax2, Atp6v1b1, Ankrd53, Tex261, Nagk, Paip2b, Zfml, Dysf, Cyp26b1, Exoc6b, Npm3-ps1, Gm5878, Spr, Emx1, Sfxn5, Rab11fip5, Noto, Smyd5, 1700040I03Rik, Cct7, Fbxo41, Egr4, Alms1, Cml3, Cml5, Cml4, Cml2, 1700019G17Rik, Cml1, Tprkb, Dusp11, Figla, Add2, Tgfa, Fam136a, Snrpg, Pcyox1, Tia1, C87436, Pcbp1, Asprv1, Mxd1, Snrnp27, Gmcl1, Anxa4, 2610306M01Rik, Aak1, Nfu1, Gfpt1, Antxr1, Gkn1, Gkn2, 1190003M12Rik, Bmp10, Arhgap25, Prokr1, Aplf, Ccdc48, Gp9, Rab43, Isy1, Cnbp, Copg, 8430410A17Rik, H1fx, Gm5577, Rab7, Rpn1, Gata2, Dnajb8, Eefsec, Ruvbl1, Sec61a1, Klhdc6, Mgll, Abtb1, Podxl2, Mcm2, Tpra1, 4933427D06Rik, Gm1965, Gm839, Plxna1, Chchd6, Txnrd3, V1rb7, V1rb10, V1rb9, V1ra6, V1ra5, V1rb4, V1ra2, V1rb8, V1ra4, V1ra3, V1rb2, V1rb1, V1ra1, V1ra7, V1ra8, V1rb3, V1ra9, BC048671, Chst13, Uroc1, Zxdc, Ccdc37, Klf15, Aldh1l1, Slc41a3, Iqsec1, Nup210, Hdac11, Fbln2, Wnt7a, Chchd4, Tmem43, Xpc, Lsm3, Slc6a6, Grip2, C130022K22Rik, 4930590J08Rik, Fgd5, Nr2c2, Mrps25, Zfyve20, Trh, Prickle2, Adamts9, Magi1, Slc25a26, Lrig1, Kbtbd8, Suclg2, Fam19a1, 1700123L14Rik
(-)/(+)_No.3	chr14	qD3 - qE2.3	76841266	1.02E+08	1235	0.545352	0	0	Tsc22d1, Serp2, 1700108F19Rik, 9030625A04Rik, Ccdc122, Enox1, Gm6994, Gm1587, Dnajc15, Epsti1, AU021034, Tnfsf11, Dgkh, 1300010F03Rik, AU017455, 1190002H23Rik, Narg1l, Mtrf1, Kbtbd7, Gm5465, Wbp4, Elf1, Sugt1, Lect1, Pcdh8, Olfm4, Pcdh17, Diap3, Tdrd3, Pcdh20, Gm5088, Pcdh9, 4921530L21Rik, Klhl1, Dach1, 2410129H14Rik, 6720463M24Rik, Dis3, Pibf1, Klf5, Klf12, 1700110M21Rik
(+)/(+)_No.1	chr4	qC6	101455949	1.02E+08	9	0	-0.73734	2.52E-24	Lepr, B020004J07Rik
(+)/(+)_No.1	chr5	qA3	19051656	19551538	38	0	-2.94853	0	Magi2
(+)/(+)_No.1	chr8	qA1.1 - qE2	3130823	1.32E+08	8151	0.48887	0	0	Insr, A430078G23Rik, Arhgef18, Pex11c, 1700019B03Rik, Zfp358, Mcoln1, Pnpla6, C330021F23Rik, 2310057J16Rik, Xab2, Pcp2, Stxbp2, Retn, 1810033B17Rik, Trappc5, Fcer2a, Clec4g, Cd209a, Rprl3, Cd209e, Cd209d, Cd209b, Cd209c, Cd209f, Evi5l, BC068157, Lrrc8e, Map2k7, Snapc2, Ctxn1, Timm44, Elavl1, Ccl25, Lass4, BC003267, Shcbp1, Slc10a2, Efnb2, Arglu1, Fam155a, Lig4, Abhd13, Tnfsf13b, Myo16, Irs2, Col4a1, Col4a2, Rab20, Carkd, Cars2, Ing1, Ankrd10, 1700016D06Rik, Gm6524, Arhgef7, 1700018L24Rik, Gm5607, Sox1, 1700094C09Rik, Tubgcp3, Atp11a, Mcf2l, F7, F10, Proz, Pcid2, Cul4a, Lamp1, Grtp1, Adprhl1, Dcun1d2, Tmco3, Tfdp1, Atp4b, Grk1, Fam70b, Gas6, 1700029H14Rik, Rasa3, 4932443I19Rik, Cdc16, Upf3a, AF366264, Zfp828, 2410022L05Rik, Fbxo25, 2610019F03Rik, Erich1, Dlgap2, Cln8, Arhgef10, Kbtbd11, BB014433, Myom2, Csmd1, Mcph1, Angpt2, Agpat5, Xkr5, Defb40, Defb37, Defb38, Defb39, Defb12, Defb34, Spag11b, Spag11a, Defb14, Defb4, Defa-ps12, Defb6, Defb46, Defb5, Defb3, Defa-ps13, Defb8, Defb7, Gm6040, AY761185, Defa21, Defa-rs7, Defa23, Defa25, Defa22, Defa3, Defa5, Defcr-rs1, Defa20, Defa26, Defa4, Defa-ps1, AY761184, Defcr6, Defa24, Defb1, Defb50, Defb2, Defb10, Defb9, Defb11, Defb15, Defb35, Defb13, C86695, Ccdc70, Atp7b, Alg11, Nek5, Nek3, Ckap2, Vps36, Thsd1, Tpte, Slc25a15, Mrps31, AI316807, Slc20a2, Vdac3, Dkk4, Polb, A930013F10Rik, Ikbkb, Plat, Ap3m2, Myst3, Ank1, Nkx6-3, Agpat6, Gins4, Golga7, Sfrp1, Zmat4, 1810011O10Rik, Ido2, Ido1, Adam18, Adam3, Adam5, Adam32, Adam9, Tm2d2, Htra4, Plekha2, Tacc1, Fgfr1, Letm2, Whsc1l1, Ppapdc1b, Ddhd2, Bag4, Lsm1, Star, Ash2l, Kcnu1, Hgsnat, 4930444A02Rik, Fnta, Hook3, Rnf170, Thap1, Zfp703, Erlin2, Prosc, Gpr124, Brf2, Rab11fip1, Got1l1, Adrb3, Eif4ebp1, Gm9731, Tex24, Chrnb3, Chrna6, 4921537P18Rik, Unc5d, Dusp26, Rnf122, BC019943, Mak16, Fut10, Nrg1, Wrn, Purg, Tex15, Ppp2cb, Ubxn8, Gsr, Gtf2e2, Rbpms, Dctn6, Mboat4, Leprotl1, Tmem66, Dusp4, Tnks, Ppp1r3b, Eri1, Mfhas1, Cldn23, D8Ertd82e, Lonrf1, 6430573F11Rik, Dlc1, A730069N07Rik, AI429214, Sgcz, Tusc3, Msr1, Fgf20, Efha2, Zdhhc2, Cnot7, Vps37a, Mtmr7, Adam24, Adam25, Adam38, Adam39, Slc7a2, Pdgfrl, Mtus1, Fgl1, Pcm1, Asah1, Frg1, Zfp353, Triml1, Triml2, Zfp42, Adam26b, Adam26a, Gm5346, Adam34, Gm5347, Fat1, Mtnr1a, F11, Klkb1, Cyp4v3, Fam149a, Tlr3, Sorbs2, Pdlim3, Ccdc110, 1700029J07Rik, Ufsp2, Ankrd37, Lrp2bp, Snx25, 4933411K20Rik, Slc25a4, Helt, Acsl1, Mlf1ip, Ccdc111, Casp3, Irf2, Enpp6, Stox2, D030016E14Rik, Rwdd4a, Ing2, Cdkn2aip, LOC100039801, Cldn22, Wwc2, Dctd, Odz3, Aga, Neil3, Vegfc, Spcs3, Asb5, Spata4, Wdr17, Gpm6a, Adam29, Glra3, Hpgd, BC088983, Fbxo8, Hand2, Scrg1, Sap30, Hmgb2, Galnt7, 4930431L04Rik, BC030500, Aadat, Mfap3l, 2700029M09Rik, Clcn3, Nek1, Sh3rf1, Cbr4, Palld, Ddx60, Anxa10, Spock3, Tll1, Cpe, Sc4mol, Klhl2, Tmem192, Trim75, Trim60, Trim61, March1, 1810029B16Rik, Tktl2, Npy5r, Npy1r, Naf1, Nat1, Nat2, Nat3, Psd3, Sh2d4a, Csgalnact1, Ints10, Lpl, Slc18a1, Atp6v1b2, Lzts1, D10627, D130040H23Rik, Zfp868, Zfp869, D330038O06Rik, Atp13a1, Gmip, Lpar2, Pbx4, Cilp2, Ndufa13, Tssk6, Gatad2a, 9130404D08Rik, Sf4, Tm6sf2, Hapln4, Ncan, Rfxank, Nr2c2ap, 2310045N01Rik, Mef2b, Tmem161a, Slc25a42, Armc6, Sfrs14, Homer3, Ddx49, Cope, Gdf1, Lass1, Upf1, Comp, Crtc1, Klhl26, Tmem59l, Crlf1, 2810428I15Rik, Uba52, Gm11808, 2810422J05Rik, Fkbp8, Ell, Isyna1, Ssbp4, Lrrc25, Gdf15, Pgpep1, Lsm4, Jund, Pde4c, Rab3a, Mpv17l2, Ifi30, Pik3r2, Il12rb1, Arrdc2, Kcnn1, Ccdc124, Slc5a5, Rpl18a, Gm15427, Snora68, Mtap1s, Haus8, Myo9b, Use1, Ocel1, Nr2f6, Ushbp1, 5430437P03Rik, Ankle1, Abhd8, Mrpl34, Dda1, Gtpbp3, Plvap, Bst2, Fam125a, Tmem221, Nxnl1, Slc27a1, Pgls, Glt25d1, Unc13a, Jak3, Insl3, B3gnt3, Fcho1, Zfp709, Zfp617, Cyp4f18, Olfr372, Olfr373, Olfr374, Tpm4, Rab8a, Hsh2d, 2510049I19Rik, Ap1m1, Klf2, Eps15l1, Calr3, 1700030K09Rik, Cherp, Slc35e1, Med26, 9130011J15Rik, Tmem38a, Nwd1, Sin3b, F2rl3, Large, Isx, Hmgxb4, Tom1, Hmox1, Mcm5, Rasd2, Nr3c2, Arhgap10, 0610038B21Rik, Prmt10, Tmem184c, Ednra, Ttc29, Pou4f2, Rbmxrt, Slc10a7, Lsm6, Zfp827, 1700011L22Rik, Mmaa, Smad1, Otud4, Abce1, Anapc10, Hhip, Gypa, Smarca5, Gab1, Usp38, Inpp4b, Il15, Zfp330, Rnf150, Tbc1d9, Ucp1, Elmod2, 4933434I20Rik, Clgn, Scoc, Olfr370, Ndufb7, Tecr, Dnajb1, Gipc1, Ptger1, Pkn1, Ddx39, Cd97, Lphn1, Asf1b, Prkaca, Samd1, 1700067K01Rik, 4432412L15Rik, Il27ra, Rln3, Rfx1, Dcaf15, Podnl1, Cc2d1a, 4930432K21Rik, Nanos3, Zswim4, D8Ertd738e, Mri1, Ccdc130, Cacna1a, Ier2, Nacc1, Trmt1, Lyl1, Nfix, Dand5, Gadd45gip1, Rad23a, Calr, Farsa, Syce2, Gcdh, Klf1, Dnase2a, Mast1, Rtbdn, Rnaseh2a, Prdx2, Junb, Hook2, Best2, Asna1, 2310036O22Rik, Tnpo2, Fbxw9, Dhps, 1500041N16Rik, BC056474, Man2b1, Zfp791, Gm6531, Olfr371, Vps35, Orc6l, Mylk3, 4921524J17Rik, Gpt2, Dnaja2, Neto2, Itfg1, Phkb, Abcc12, Lonp2, Siah1a, Gm10638, N4bp1, Cbln1, 4933402J07Rik, Zfp423, Tmem188, Heatr3, Adcy7, Brd7, Nkd1, Snx20, Nod2, Cyld, Sall1, Tox3, Chd9, Rbl2, Aktip, Rpgrip1l, Fto, Irx3, Irx5, Irx6, Mmp2, Lpcat2, Capns2, Slc6a2, Gm4976, Gm5158, Es1, Ces3, Es22, AU018778, Ces1, Ces7, Gnao1, 4930488L21Rik, Amfr, Nudt21, Ogfod1, Bbs2, Mt4, Mt3, Mt2, Mt1, Nup93, Slc12a3, Herpud1, Nlrc5, Cpne2, 2310065K24Rik, Rspry1, Arl2bp, Pllp, Ccl22, Cx3cl1, Ccl17, Ciapin1, Coq9, Polr2c, Dok4, Ccdc102a, Gpr114, Gpr56, Gpr97, Ccdc135, Katnb1, Kifc3, BC016201, Tepp, Zfp319, AA960436, Mmp15, Gtl3, Csnk2a2, Ccdc113, 4931432M23Rik, Gins3, Ndrg4, Setd6, Cnot1, Slc38a7, Got2, Cdh8, Cdh11, Cdh5, Bean, Tk2, Cklf, Cmtm2a, Cmtm2b, Cmtm3, Cmtm4, Dync1li2, Ccdc79, Nae1, Car7, Pdp2, Cdh16, Rrad, Fam96b, Ces6, BC015286, Ces2, Ces5, 2310038E17Rik, 2210023G05Rik, Gm4738, Ces8, Cbfb, D230025D16Rik, B3gnt9, Tradd, Fbxl8, Hsf4, Nol3, 4931428F04Rik, Exoc3l, E2f4, Elmo3, Lrrc29, Tmem208, Fhod1, Slc9a5, Plekhg4, Kctd19, Lrrc36, Tppp3, Zdhhc1, Hsd11b2, Atp6v0d1, Agrp, Fam65a, Ctcf, Rltpr, Acd, Pard6a, E130303B06Rik, 4933405L10Rik, Gfod2, Ranbp10, Tsnaxip1, Cenpt, Thap11, Nutf2, Edc4, Nrn1l, Pskh1, Ctrl, Psmb10, Lcat, Slc12a4, Dpep3, Dpep2, Ddx28, Dus2l, Nfatc3, Esrp2, Pla2g15, Slc7a6, Slc7a6os, Prmt7, Smpd3, Zfp90, Cdh3, Cdh1, Tmco7, Has3, Chtf8, Cirh1a, Sntb2, Vps4a, Pdf, Cog8, Nip7, Tmed6, Terf2, Cyb5b, Nfat5, Nqo1, Nob1, Wwp2, Gm10070, Psmd7, Zfhx3, Gm1943, Pmfbp1, Dhx38, Txnl4b, Hp, Dhodh, Pkd1l3, 2400003C14Rik, Zfp821, Atxn1l, Ap1g1, Phlpp2, Marveld3, Tat, Chst4, Zfp612, Calb2, Ftsjd1, Hydin, Vac14, Mtss1l, Il34, Sf3b3, Cog4, Fuk, St3gal2, Ddx19a, Ddx19b, Aars, Exosc6, Clec18a, 4930402E16Rik, Glg1, Rfwd3, Mlkl, Fa2h, Wdr59, Znrf1, Ldhd, Zfp1, Ctrb1, Bcar1, Cfdp1, Tmem170, Chst5, 4932417I16Rik, Gabarapl2, Adat1, Kars, Terf2ip, Cntnap4, Mon1b, 4930481F22Rik, Adamts18, Nudt7, Vat1l, Clec3a, Wwox, Maf, Dynlrb2, Cdyl2, 2310061C15Rik, Cenpn, Atmin, 1700030J22Rik, Gcsh, Pkd1l2, Bcmo1, Gan, 4933407C03Rik, Plcg2, Sdr42e1, Hsd17b2, Mphosph6, Cdh13, Hsbp1, Mlycd, Osgin1, Necab2, Slc38a8, Mbtps1, Hsdl1, Lrrc50, Taf1c, Adad2, Kcng4, Wfdc1, Atp2c2, 4632415K11Rik, Cotl1, Klhl36, Usp10, Crispld2, Zdhhc7, 6430548M08Rik, Fam92b, Gse1, Gins2, 1190005I06Rik, Cox4nb, Cox4i1, Irf8, Foxf1a, Mthfsd, Foxc2, Foxl1, 1700018B08Rik, Fbxo31, Map1lc3b, Zcchc14, Jph3, Klhdc4, Slc7a5, BC048644, Car5a, Banp, Zfpm1, Trhr2, Zc3h18, Il17c, Cyba, Mvd, Snai3, Rnf166, 2310061F22Rik, Cdt1, Aprt, Galns, Trappc2l, Pabpnl1, Cbfa2t3, Acsf3, Gm16378, Cdh15, Ankrd11, Spg7, Rpl13, Snord68, Cpne7, Sult5a1, Dpep1, Chmp1a, 4732415M23Rik, Cdk10, Spata2L, 1300018I17Rik, Zfp276, Fanca, Spire2, Tcf25, Mc1r, Tubb3, Def8, Afg3l1, Dbndd1, Gas8, Rhou, Rab4a, 1700054N08Rik, Acta1, Nup133, Abcb10, Taf5l, Urb2, Galnt2, Pgbd5, Cog2, Agt, Capn9, 2310022B05Rik, Ttc13, Arv1, Fam89a, Trim67, 2810004N23Rik, Gnpat, Exoc8, Gm505, Egln1, Tsnax, Disc1, Sipa1l2, 4933403G14Rik, 2310079N02Rik, Pcnxl2, BC021891, Kcnk1, Slc35f3, 1810063B05Rik, Tarbp1, Tomm20, Rbm34, Pard3, Nrp1, Itgb1, Ccdc7, 1700008F21Rik
(+)/(+)_No.1	chrY	qA1	37653	2635638	121	0	-0.52334	7.71E-162	Zfy1, Ube1y1, Kdm5d, Eif2s3y, Tspy-ps, Uty, Ddx3y, Usp9y, Zfy2, Gm16501, Sry, Rbmy1a1
(+)/(+)_No.2	chr5	qE1	90304287	90321465	3	0	-0.94994	7.67E-14	Adamts3
(+)/(+)_No.2	chr11	qA1 - qE2	3030764	1.22E+08	8992	0.399104	0	0	[illegible]
(+)/(+)_No.2	chr15	qD3	73018495	73298481	28	0	-0.74953	5.78E-78	Ptk2
(+)/(+)_No.2	chrY	qA1	37653	2529532	117	0	-3.04195	0	Zfy1, Ube1y1, Kdm5d, Eif2s3y, Tspy-ps, Uty, Ddx3y, Usp9y, Zfy2, Gm16501, Sry, Rbmy1a1
(+)/(+)_No.2	chrY	qA1	253207	273823	4	0	-5.35883	3.61E-16	Kdm5d
(+)/(+)_No.2	chrY	qA1	346878	374359	5	0	-4.98972	2.28E-16	Eif2s3y
(+)/(+)_No.2	chrY	qA1	434833	731591	40	0	-4.17578	2.55E-54	Uty, Ddx3y, Usp9y
(+)/(+)_No.2	chrY	qA1	801912	1356694	13	0	-4.59496	9.90E-29	
(+)/(+)_No.3	chr12	qC3	76835388	76958575	14	0.782565	0	3.08E-33	Sgpp1, Syne2

Cell	Methods	Total reads	mapped reads		unmapped reads	
mouse-iPS	(-)/(+)	53773236	45988390	85.52%	7784846	14.48%
mouse-iPS	(-)/(+)	57806362	49041112	84.84%	8765250	15.16%
mouse-iPS	(-)/(+)	54591766	47091620	86.26%	7500146	13.74%
mouse-iPS	(+)/(+)	55474387	47075833	84.86%	8398554	15.14%
mouse-iPS	(+)/(+)	51188601	43300760	84.59%	7887841	15.41%
mouse-iPS	(+)/(+)	55114847	46748840	84.82%	8366007	15.18%
fibroblast		50539652	43232627	85.54%	7307025	14.46%

Table S2. Sequencing summary for MBD-seq

Total reads: total number of reads for raw MBD-seq data in each sample. Mapped reads: number and percentage of MBD-seq reads mapped to mouse genome reference sequence (mm9). Unmapped reads: number and percentage of MBD-seq reads unmapped to mouse genome reference sequence (mm9).

Top Bio Functions

Diseases and Disorders	p-value	# of Molecules
Cancer	4.86E-06 - 1.09E-02	166
Organismal Inury and Abnormalities	1.18E-05 - 1.02E-02	58
Inflammatory Disease	3.01E-05 - 1.02E-02	16
Respiratory Disease	3.01E-05 - 6.91E-03	43
Connective Tissue Disorders	7.68E-05 - 1.02E-02	40
**Molecular and Cellular Functions**		
Cell Morphology	1.52E-06 - 1.04E-02	69
Cellular Assembly and Organization	1.52E-05 - 1.04E-02	75
Cell Cycle	9.12E-05 - 1.02E-02	66
Cell-To-Cell Signaling and Interaction	1.72E-05 - 1.10E-02	49
Cellular Compromise	1.99E-05 - 1.10E-02	24
**Physiological System Development and Function**		
Organismal Functions	1.24E-07 - 9.28E-03	27
Tissue Morphology	1.24E-07 - 1.04E-02	68
Cardiovascular System Development and Function	2.98E-07 - 1.02E-02	75
Organismal Development	2.64E-06 - 1.10E-02	122
Tissue Development	1.72E-05 - 1.10E-02	120

Table S3. Gene ontology analysis of differentially expressed genes (Data represent mean ± SD of three biological replicates).

Gene name	Fowrard primer	Reverse primer
Nanog	AGGACAGGTTTCAGAAGCAGA	CCATTGCTAGTCTTCAACCACTG
Oct3/4	CGGAAGAGAAAGCGAACTAGC	ATTGGCGATGTGAGTGATCTG
Klf4 (Tg)	GGACCACCTTGCCTTACACA	TGGCCTGCCCGGTTATTATT
Sox2 (Tg)	GGTTACCTCTTCCTCCCACTCCAG	TTATCGTCGACCACTGTGCTGCTG
c-Myc (Tg)	CAGAGGAGGAACGAGCTGAAGCGC	TTATCGTCGACCACTGTGCTGCTG
Oct3/4 (Tg)	CTTTCCCTCTGTTCCCGTCA	GGTAATGGTAGCGACCGGC
Oct3/4 (endo)	ATTCCCAACGAGAAGAGTATGA	TCCCTTGCCTTGGCTCACAG
β-actin	GAAATCGTGCGTGACATCAAAG	TGTAGTTTC ATGGATGCCACAG
Nestin	CCCCTTGCCTAATACCCTTGA	GCCTCAGACATAGGTGGGATG
Pax6	TACCAGTGTCTACCAGCCAAT	TGCACGAGTATGAGGAGGTCT
Isl1	ATGATGGTGGTTTACAGGCTAAC	TCGATGCTACTTCACTGCCAG
Sox17	GATGCGGGATACGCCAGTG	CCACCACCTCGCCTTTCAC
FoxA2	TCCGACTGGAGCAGCTACTAC	GCGCCCACATAGGATGACA
FoxC1	CCCCGGACAAGAAGATCACTC	AGGTTGTGCCGTATGCTGTTC
Flk-1	TTTGGCAAATACAACCCTTCAGA	GCAGAAGATACTGTCACCACC
Zscan4	CCTATGTAGCCGTCGCTTTC	AAGTGGCAATCCACAAGCAT
muERV-L	CCCATCATGAGCTGGGTACT	CGTGCAGAGCCATCAGTAAA
Tcstv1	GCCCAGAGTACAAGGTGTTCTAAT	ATTCAATCTTCGGTAGGATCTCAG
Eif1a	AAGAAGTCTGAAGGCCTATG	CAGAGAACTTGGAAGGTAGC

Table S4. Primers used in this study.

Antibody	Source	Catalogue number
Nanog	Abcam	ab80892
Oct3/4	Santa Cruz	SC5279
Oct4	Santa Cruz	sc9081
SSEA1	Abcam	MC480
SSEA3	Abcam	MC631
SSEA-4	Abcam	MC813
TRA-1-60	Millipore	MAB4360
TRA-1-81	Millipore	MAB4381
Zscan4	Abnova	H00201516-B01P
EIf1a	Abcam	ab172623 (177939)
β -III-Tubulin	Sigma	T8660
GFAP	Dako	N1506

Table S5. Antibodies used in this study.
