# Supplementary material for: Induced Pluripotent Stem Cells Reprogrammed with Three Inhibitors Show Accelerated Differentiation Potentials with High Levels of 2-Cell Stage Marker Expression
Source: Stem Cell Reports. 2019 Jan 31;12(2):305–18. doi: 10.1016/j.stemcr.2018.12.018 (PMC6373546; doi:10.1016/j.stemcr.2018.12.018)
Supplement: Document S2. Article plus Supplemental Information [file mmc2.pdf]

Stem Cell Reports
Article

OPEN ACCESS

Induced Pluripotent Stem Cells Reprogrammed with Three Inhibitors Show Accelerated Differentiation Potentials with High Levels of 2-Cell Stage Marker Expression

Koji Nishihara,[1,6] Takahiro Shiga,[2,6] Eri Nakamura,[3] Tomohiko Akiyama,[4] Takashi Sasaki,[5] Sadafumi Suzuki,[1] Minoru S.H. Ko,[4] Norihiro Tada,[3] Hideyuki Okano,[1,*] and Wado Akamatsu[1,2,*]

[1]Department of Physiology, Keio University School of Medicine, 35 Shinanomachi, Shinjuku-ku, Tokyo 160-8582, Japan
[2]Center for Genomic and Regenerative Medicine, Juntendo University School of Medicine, 2-1-1 Hongo, Bunkyo-ku, Tokyo 113-8421, Japan
[3]Research Institute for Diseases of Old Age, Juntendo University School of Medicine, 2-1-1 Hongo, Bunkyo-ku, Tokyo 113-8421, Japan
[4]Department of Systems Medicine, Sakaguchi Laboratory, Keio University School of Medicine, 35 Shinanomachi, Shinjuku-ku, Tokyo 160-8582, Japan
[5]Center for Supercentenarian Medical Research, Keio University School of Medicine, 35 Shinanomachi, Shinjuku-ku, Tokyo 160-8582, Japan
[6]Co-first author
*Correspondence: hidokano@a2.keio.jp (H.O.), awado@juntendo.ac.jp (W.A.)
https://doi.org/10.1016/j.stemcr.2018.12.018

SUMMARY

Although pluripotent stem cells can generate various types of differentiated cells, it is unclear why lineage-committed stem/progenitor cells derived from pluripotent stem cells are decelerated and why the differentiation-resistant propensity of embryonic stem cell (ESC)/induced pluripotent stem cell (iPSC)-derived cells is predominant compared with the *in vivo* equivalents derived from embryonic/adult tissues. In this study, we demonstrated that iPSCs reprogrammed and maintained with three chemical inhibitors of the fibroblast growth factor 4-mitogen-activated protein kinase cascade and GSK3β (3i) could be differentiated into all three germ layers more efficiently than the iPSCs reprogrammed without the 3i chemicals, even though they were maintained with 3i chemicals once they were reprogrammed. Although the iPSCs reprogrammed with 3i had increased numbers of Zscan4-positive cells, the Zscan4-positive cells among iPSCs that were reprogrammed without 3i did not have an accelerated differentiation ability. These observations suggest that 3i exposure during the reprogramming period determines the accelerated differentiation/maturation potentials of iPSCs that are stably maintained at the distinct state.

INTRODUCTION

Pluripotent stem cells (PSCs) can theoretically differentiate into derivatives of all three germ layers. Both induced pluripotent stem cells (iPSCs) and embryonic stem cells (ESCs) give rise to lineage-committed somatic stem/progenitor cells and are eventually differentiated into terminally differentiated progenies. Cell replacement therapy, drug screening, and disease modeling are facilitated by the pluripotency and self-renewal ability of these PSCs, which can induce various disease-relevant cell types (Shi et al., 2017; Takahashi and Yamanaka, 2006). However, it is still unclear whether lineage-committed stem/progenitor cells derived from embryonic/adult tissues and PSCs have identical differentiation abilities. A clear difference between tissue- and PSC-derived cells is observed during differentiation into the differentiated progenies. This observation is especially true for human PSCs, where a period of approximately 2–5 months is required for *in vitro* differentiation into hepatocytes (Ma et al., 2013), oligodendrocytes (Numasawa-Kuroiwa et al., 2014), or retinal pigment epithelia (Jin et al., 2011). These observations strongly suggest that the differentiation/maturation of PSC-derived cells is significantly slower than that of equivalents in *in vitro* primary cultures. Regarding neural differentiation *in vitro*, whereas embryonic day 6.5 (E6.5) mouse epiblast-derived neurospheres acquire a responsiveness to fibroblast growth factor (FGF)/epidermal growth factor (EGF) that is characteristic of mature (committed) definitive neural stem cells (NSCs) after a few passages, ESC-derived neurospheres are unable to acquire FGF dependency, even after recurrent passages without exogenous active Notch transduction (Hitoshi et al., 2004). This slow and inefficient differentiation/maturation of PSC-derived cells leads not only to difficulties in the preparation of the desired cells but also to aneuploidy or tumor formation due to the long *in vitro* cultivation period (Conti and Cattaneo, 2010). However, for the cell-based therapy of several diseases with progressive and changeable features (e.g., spinal cord injury [Nagoshi and Okano, 2017], ischemic stroke [Tornero et al., 2013], or acute myocardial infarction [Nelson et al., 2009]), rapid preparations of donor cells are necessary due to limited therapeutic windows of time. Therefore, it may be difficult to prepare iPSC-derived cells for autologous and allogeneic transplantations, and cells may need to be selected despite the risk of immunorejection and infection for these diseases. To contribute to the future regenerative medicine, we aimed to solve this problem by establishing iPSCs with fast and efficient differentiation or maturation potentials compared with the iPSCs that are established by current protocols.

This is an open access article under the CC BY license (http://creativecommons.org/licenses/by/4.0/).

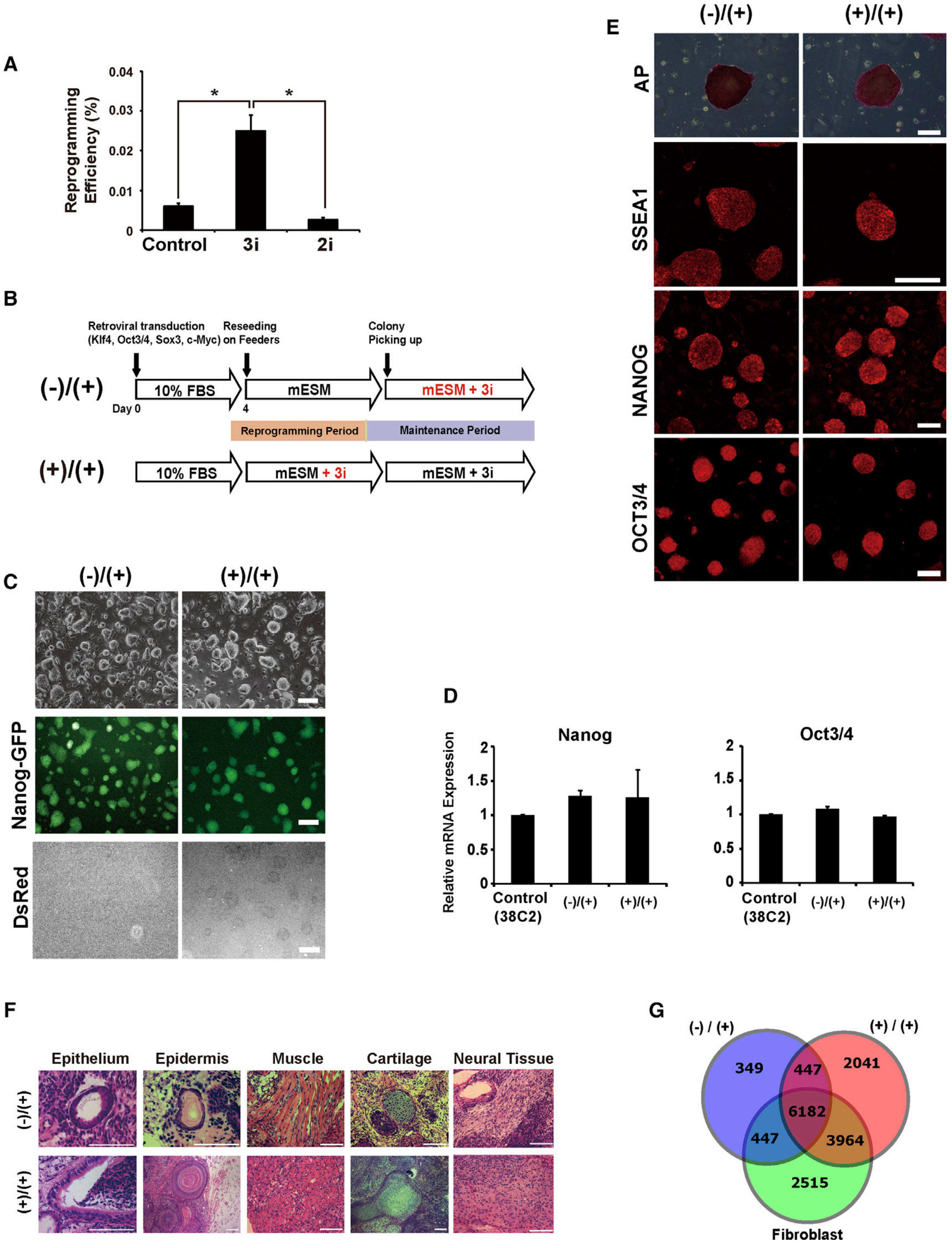

Figure 1. Generation of Two Groups of Murine iPSCs Using Small Molecules

(A) The reprogramming efficiency of 3i- or 2i-treated fibroblasts compared with untreated cells is shown. iPSC colonies were identified based on ESC-like morphology and expression of *Nanog-GFP* detected under a fluorescence microscope (n = 3; $^*p < 0.05$).

(B) The schematic representations of two reprogramming protocols used in this study. In our protocol, *Nanog-GFP* transfected fibroblasts were cultured in 10% FBS medium, re-seeded on feeders, and switched to murine ESC medium. While the 3i chemicals were used only after colonies were picked (maintenance period) in the (−)/(+) condition, the 3i chemicals were used during both the reprogramming phase

(legend continued on next page)

Recent studies have demonstrated that some chemical cocktails containing FGF4- mitogen-activated protein kinase (MAPK) cascade/GSK3β inhibitors (so-called 2i and 3i) contribute to the authentic and homogeneous naive pluripotency of iPSCs (Choi et al., 2017; Marks et al., 2012; Ying et al., 2008) and promote reprogramming efficiency (Silva et al., 2008; Valamehr et al., 2014). Although a few studies have claimed that conversion into a ground (or ground-like) state improves the differentiation potentials of iPSCs (Duggal et al., 2015; Honda et al., 2013), the effect of these chemicals on the differentiation potency of iPSCs remains controversial (Chan et al., 2013; Gafni et al., 2013; Takashima et al., 2014; Theunissen et al., 2014; Valamehr et al., 2014). Given that the mechanism for acquiring pluripotency is drastic epigenetic reprogramming and that the epigenetic memory of the original somatic cells in iPSCs influences their differentiation potential, we hypothesized that the addition of these chemicals during a reprogramming period influenced the *in vitro* differentiation/maturation potential of iPSCs. To test this hypothesis, we generated two groups of murine iPSCs using these chemicals during two different periods (only a maintenance period or both a reprogramming and maintenance period) and found that their differentiation potentials are significantly different.

RESULTS

Generation of Murine iPSCs with Pluripotency-Enhancing Chemicals

First, we speculated that the reprogramming period, not the maintenance period, in clonally generated iPSC lines could influence the differentiation/maturation potential. To test whether using chemicals that support cellular reprogramming and/or pluripotency during the reprogramming period could regulate the differentiation potentials of iPSCs, we used these chemicals during cellular reprogramming into iPSCs with different time courses. We used three chemicals that inhibit FGF receptor tyrosine kinase (SU5402), ERK1/2 (PD184352 or PD0325901), and GSK3β (CHIR99021) as representative chemical molecules that support pluripotency (Ying et al., 2008).

First, we tested whether 2i (PD0325901 and CHIR99021) or 3i (PD184352, CHIR99021, and SU5402) had any effects on reprogramming efficiency and on maintenance of pluripotency. We reprogrammed mouse embryonic fibroblasts (MEFs) derived from *Nanog*-GFP-IRES-Puro transgenic mice (Okita et al., 2007) using retroviruses harboring four transcription factors: *Oct3/4*, *Sox2*, *Klf4*, and *c-Myc* (KSOM). dsRed transgenes were also infected simultaneously as an indicator of transgene silencing. We began to add 2i/3i on day 4 after infection because previous reports demonstrated that KSOM-transduced MEFs underwent a mesenchymal-to-epithelial transition around day 5 after infection in the initiation phase, followed by the expression of SSEA1 and NANOG in the maturation phase (Li et al., 2010; Polo et al., 2010). We quantified the number of generated GFP^+ $dsRed^-$ ESC-like colonies during reprogramming with or without 2i/3i and revealed that 3i increased the number of GFP^+ $dsRed^-$ ESCs, in the form of colonies, when examined at 3 weeks post-infection, while 2i had no significant effect on colony formation efficiency (Figure 1A). These data suggested that the addition of 3i during the reprogramming period enhanced the reprogramming efficiency and increased the number of colonies compared with the conventional condition without 3i. We hypothesized that the higher number of colonies that appeared with the sequential addition of 3i during the reprogramming and maintenance period would not appear in the conventional condition without 3i. Thus, we used the 3i chemicals as the model for the reprogramming molecules in this study and investigated the relationship between the reprogramming conditions and differentiation potential of iPSCs.

(including the period from day 2 to day 12) and the maintenance phase in the (+)/(+) condition. The 3i chemicals were used at the following concentrations: 3 μM CHIR99021, 0.8 μM PD184352, and 0.8 μM SU5402.
(C) Representative images of iPSC colonies generated using the (−)/(+) or (+)/(+) protocol. Both colony types exhibited a typical ESC morphology and expressed *Nanog-GFP* homogeneously. The silencing efficiency of dsRed, which was transduced together with OSKM as a transgene control while generating the iPSCs, was verified. Scale bar, 100 μm.
(D) qPCR for expression of pluripotency markers (*Nanog* and *Oct3/4*) in iPSCs. The averages and SD of three different clones of the (−)/(+) or (+)/(+) iPSC lines are represented. 38C2 is a control iPSC clone that was derived from the same *Nanog*-GFP mouse line (n = 3; $^*p < 0.05$).
(E) Representative images of AP staining and immunofluorescence staining of pluripotent markers (SSEA1, Nanog, and OCT3/4) in both the (−)/(+) and (+)/(+) iPSC lines. Scale bars, 200 μm (AP staining) and 100 μm (immunofluorescence staining).
(F) H&E staining of teratomas derived from (−)/(+) or (+)/(+) iPSCs. Cells were transplanted into the testes of severe combined immunodeficiency mice. After 3 weeks, tumors were sectioned. Gut-like epithelial tissues (left), epidermal tissues, striated muscles, cartilage, and neural tissues (right) are shown. Scale bars, 200 μm.
(G) The results of comprehensive DNA methylation analysis with MBD-seq. Venn diagram of unique and shared genes with methylated regions among (−)/(+) iPSCs (blue), (+)/(+) iPSCs (red), and fibroblasts (green) (n = 3, FDR < 0.05). Error bars represent mean ± SEM from three independent experiments (n = 3).

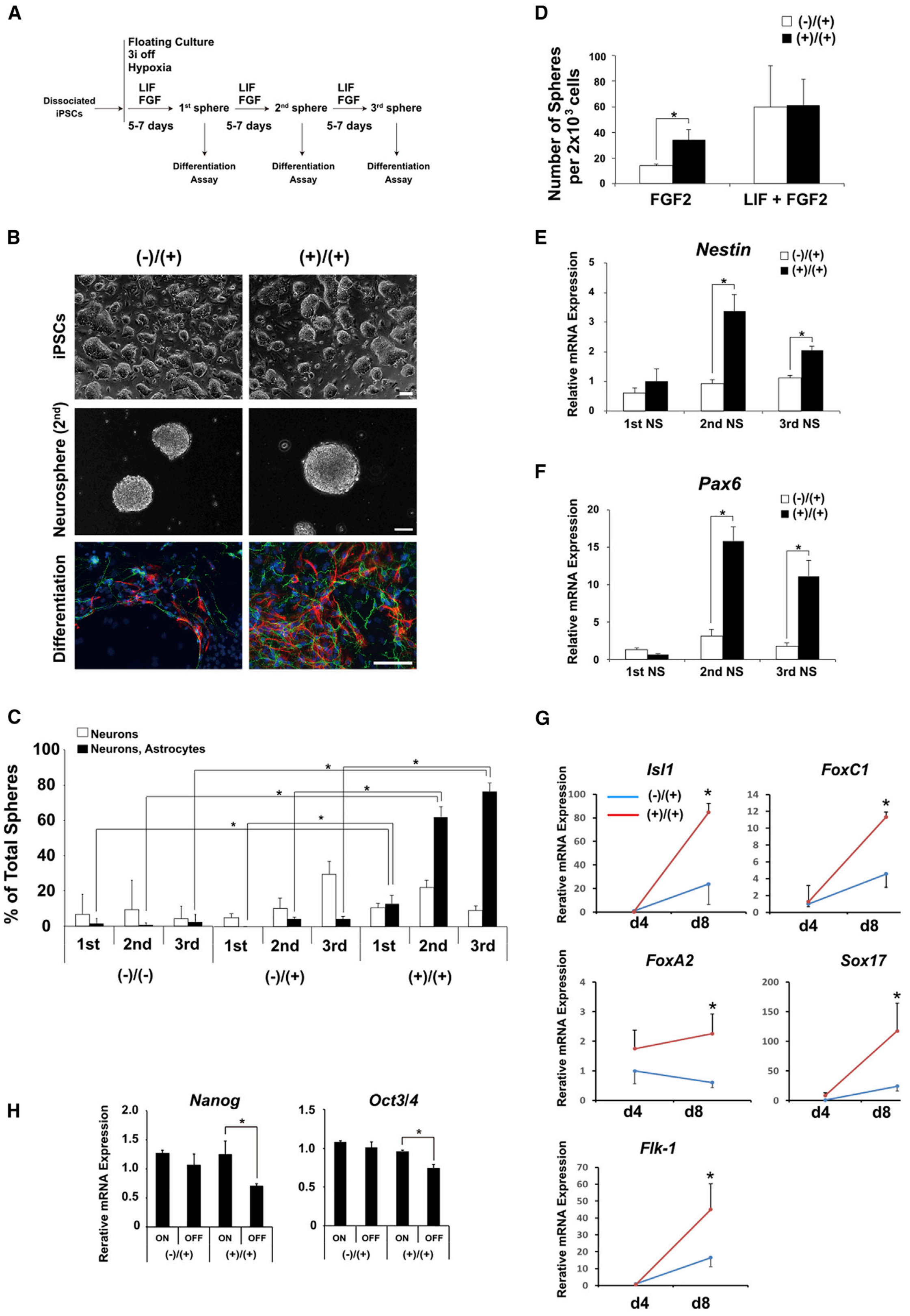

(legend on next page)

To exclude the possibility that the differences between the 3i (−) and 3i (+) iPSCs resulting from 3i usage during the reprogramming phase might be masked by 3i usage during the maintenance phase, both the 3i (−) and 3i (+) iPSCs were maintained with 3i after the reprogramming phase and are denoted as (−)/(+) and (+)/(+) iPSCs, respectively (Figure 1B). We isolated 3 clones of (−)/(+) iPSCs and 11 clones of (+)/(+) iPSCs, and all clones from both conditions exhibited similar morphologies. All clones expressed the *Nanog*-GFP fluorescence reporter but did not express dsRed due to complete silencing of the transgenes (Figure 1C). Three iPSC clones from each group, (−)/(+) and (+)/(+), were analyzed in this study. qRT-PCR analysis showed that the mRNA expression levels of pluripotent cell marker genes *Nanog* and *Oct3/4* were similar to those of control murine iPSCs (38C2), which were derived from *Nanog*-GFP transgenic animals (Okita et al., 2007) (Figure 1D). RT-PCR analysis revealed that the four transgenes were successfully silenced (Figure S1). Both the (−)/(+) and (+)/(+) colonies had a typical ESC morphology and were stained with alkaline phosphatase (AP). The expression of pluripotency markers, including NANOG and SSEA1 in both the (−)/(+) and (+)/(+) colonies was confirmed by immunocytochemical staining (Figure 1E). Teratoma formation analysis demonstrated that all generated iPSCs had similar developmental potentials to differentiate into all three germ layers (Figures 1F and S1C). We also confirmed characteristics of (−)/(+) and (+)/(+) cells as PSCs by blastocyst injection (Figure S1D). Although the efficiency of chimeras seemed relatively low, perhaps due to fetal bovine serum (FBS) usage in iPSCs, high passage numbers (more than 40) and non-biased iPSC clonal selection, we were able to obtain several chimeric animals from (−)/(+) and (+)/(+) cells. CGH array analysis demonstrated that genetic alteration frequencies were similar between (−)/(+) and (+)/(+) iPSCs (Figure S1B). To examine the global DNA methylation status of (−)/(+), (+)/(+) iPSCs and their original fibroblasts, we performed genome-wide analysis using methyl-CpG-binding domain protein 2 (MBD2)-mediated methylated DNA enrichment followed by deep sequencing (MBD-seq) (Shimamoto et al., 2014). The peaks of mapped tags were defined as methylated regions, and we compared whole-genome methylation levels. The amount of methylated DNA-enriched in (+)/(+) iPSCs was higher than that in (−)/(+) iPSCs and original fibroblasts (Table S2). These results indicated that both the (−)/(+) and (+)/(+) iPSCs were indistinguishable by pluripotency and genomic structures but have distinctly different global methylation statuses.

Accelerated Differentiation of iPSCs Established with 3i during the Reprogramming Period

Next, we analyzed the maturation and differentiation capacities of NSCs derived from both the (−)/(+) and (+)/(+) iPSC clones. By using the neurosphere formation assay, we evaluated the response to growth factors (FGF2 and leukemia inhibitory factor [LIF]) and the distribution of the differentiated progenies (neurons and astrocytes). As previously reported (Akamatsu et al., 2009; Hitoshi et al., 2002, 2004; Tropepe et al., 2001), pluripotent cells first differentiate into LIF-dependent primitive NSCs (pNSCs) and then gradually lose LIF dependency to develop into FGF-dependent definitive NSCs (Tropepe et al., 2001). The schematic schedule for neural differentiation is shown in Figure 2A. After separating from the feeder cells, iPSCs were dissociated into a low-density single-cell suspension. Dissociated iPSCs were converted to pNSCs in the presence of LIF. Then, pNSCs formed primary neurospheres after

Figure 2. Differentiation Potentials of iPSCs Generated with or without the 3i Chemicals

(A) Schematic representation of the strategy for differentiation of iPSCs to the neural lineage. iPSCs were dissociated and cultured using a suspension method to form neurospheres that were treated with LIF and basic FGF2. The resulting neurospheres were transferred onto a poly-L-ornithine/fibronectin-coated chambered slide glass to differentiate into neurons and astrocytes.

(B) Representative images of (−)/(+) and (+)/(+) iPSCs, iPSC-derived neurospheres, and immunocytochemical stains of neural cell marker proteins in the differentiated neuronal and glial cells derived from neurospheres are shown. Scale bars, 100 μm.

(C) Differentiation efficiency of the first, second, and third neurospheres derived from (−)/(−), (−)/(+), and (+)/(+) iPSCs. The frequency of colonies consisting of neurons (βIII-tubulin) and/or astrocytes (GFAP) was evaluated by immunocytochemistry and is presented as the percentage of positive colonies (n = 3; *$p < 0.05$).

(D) Second neurospheres derived from (−)/(+) or (+)/(+) iPSCs were dissociated (to a final cell density of 10 cells/μL) and grown to form neurospheres in the presence of LIF + FGF2 or FGF2 alone. The number of subcloned spheres per 2×10^3 cells is shown (n = 3; *$p < 0.05$).

(E and F) mRNA expression of markers for neural progenitors, *Nestin* (E) and *Pax6* (F) in the first, second, and third neurospheres was analyzed by qRT-PCR. The data are presented as the expression relative to that in (−)/(+) iPSCs (n = 3; *$p < 0.05$).

(G) Gene expression of early mesodermal (*Isl1* and *FoxC1*) and endodermal (*Sox17*, *FoxA2*, and *Flk-1*) markers in EBs derived from (−)/(+) and (+)/(+) iPSCs was analyzed by qRT-PCR. The data are presented as the expression relative to that in (−)/(+) iPSC-derived EBs on day 4 (n = 5; *$p < 0.05$).

(H) Relative mRNA expression of pluripotency markers (*Nanog* and *Oct3/4*) in (−)/(+) and (+)/(+) iPSCs before or after withdrawal of 3i chemicals. The averages and SD of three different clones of (−)/(+) or (+)/(+) iPSC clones are represented (n = 3, *$p < 0.05$). Error bars represent mean ± SEM from three or five independent experiments (n = 3 or 5).

cultivation for 5–7 days in serum-free medium containing LIF and FGF2. To form secondary and tertiary neurospheres, floating spheres were dissociated into single cells and cultured in the serum-free medium that contained LIF and FGF2. Secondary and tertiary neurospheres appeared 5–7 days after passaging.

Both the (−)/(+) and (+)/(+) iPSC clones formed floating spheres that were morphologically similar to each other (Figure 2B, upper and middle panels). To compare the differentiation capacity of the NSCs derived from the (−)/(+) and (+)/(+) iPSCs, the primary, secondary and tertiary neurospheres were dissociated and subjected to 7 days of adherent culture without growth factors to undergo neural differentiation (Figure 2B, lower panels). The frequencies by which neurospheres gave rise to neurons and/or astrocytes were quantified by immunocytochemistry using anti-βIII-tubulin and GFAP antibodies, respectively (Figures 2C and S2A). As another control, we analyzed NSCs derived from (−)/(−) iPSC clones that were considered as conventional iPSC clones that were generated and maintained without 3i chemicals. The primary neurospheres derived from (−)/(+) iPSC clones mainly gave rise to neurons, and few primary neurospheres that generated astrocytes were found. Although astrocytes were found in the differentiated cells from the secondary and tertiary neurospheres, frequency was very low (Figures 2C and S2A). We also examined the differentiation properties of tertiary neurospheres derived from iPSCs reprogrammed with 1i (SU5402) and 2i (PD184352 and CHIR99021). Neither 1i nor 2i iPSCs exhibited similar accelerated differentiation into neurons and astrocytes as 3i (+)/(+) iPSCs, suggesting that 3i reprogramming has the largest effect on acceleration of differentiation of iPSCs (Figures S2B and S2E).

These results were similar to those previously described for ESCs (Naka et al., 2008; Okada et al., 2008), suggesting that NSCs in the primary neurospheres derived from iPSCs have early neurogenic characteristics as do those from ESCs.

In contrast, neurospheres derived from (+)/(+) iPSC clones gave rise to a significantly increased number of astrocytes at all passages compared with those from the (−)/(+) and (−)/(−) iPSCs. Interestingly, the number of gliogenic neurospheres from the (+)/(+) iPSCs were rapidly increased during the neurosphere passages, suggesting that early neurogenic NSCs in the (+)/(+) neurospheres could develop into gliogenic mature NSCs more rapidly and efficiently than the NSCs in the (−)/(+) neurospheres. Next, we evaluated the response to growth factors in NSCs derived from the (−)/(+) and (+)/(+) iPSCs. The secondary spheres derived from the (−)/(+) and (+)/(+) iPSCs were dissociated to form tertiary spheres in the presence of LIF plus FGF2 or FGF2 alone. The neurospheres that appeared in the presence of LIF and FGF2 were considered to reflect the total number of NSCs in the dissociated cells, while neurospheres that appeared in the presence of FGF2 alone reflected the number of FGF2-dependent mature definitive NSCs (Akamatsu et al., 2009). While both the (−)/(+) and (+)/(+) NSCs formed a similar number of tertiary neurospheres in the presence of both LIF and FGF2, the (+)/(+) NSCs formed a significantly increased number of tertiary neurospheres compared with the (−)/(+) NSCs in the absence of LIF (Figure 2D). These data suggested that the (+)/(+) iPSCs developed into definitive NSCs more rapidly than the (−)/(+) iPSCs in terms of responsiveness to growth factors.

To evaluate the development of (−)/(+) and (+)/(+) NSCs per gene expression, we used qRT-PCR to measure the relative mRNA expression levels of neural progenitor markers (*Nestin* and *Pax6*) in the primary, secondary, and tertiary neurospheres. No significant difference was seen in the expression of *Nestin* and *Pax6* between primary neurospheres derived from the (−)/(+) and (+)/(+) iPSCs, while significant differences were observed in the secondary and tertiary neurospheres (Figures 2E and 2F). These results suggested that cells in the neurospheres derived from the (+)/(+) iPSCs differentiated more rapidly and efficiently than those from the (−)/(+) iPSCs.

Previous reports demonstrated that each ESC or iPSC clone had a differentiation propensity to differentiate into a certain cell lineage (Kim et al., 2010a; Osafune et al., 2008). To exclude the possibility that the differentiation propensity of the (+)/(+) iPSCs was simply biased toward the neural lineage, we evaluated the differentiation potentials of the (−)/(+) and (+)/(+) iPSCs toward the mesodermal and endodermal lineage. The (−)/(+) or (+)/(+) iPSCs were separately dissociated in suspension culture without 3i and LIF to form embryoid bodies (EBs) (Figure S3). Then, mesodermal and endodermal gene expression on day 4 and day 8 EBs was measured by qRT-PCR. In the day 8 EBs derived from the (+)/(+) iPSCs, the expression levels of early mesodermal (*Isl1* and *FoxC1*) and endodermal (*FoxA2*, *Sox17*, and *Flk-1*) markers were higher compared with the levels in the (−)/(+) iPSCs (Figure 2G). These results suggested that the differentiation potential of the (+)/(+) iPSCs was not biased toward the neural lineage and that the (+)/(+) iPSCs could also efficiently differentiate into other cell lineages (mesoderm and endoderm) compared with the (−)/(+) iPSCs. Given these observations, we hypothesized that the (+)/(+) iPSCs tended to differentiate from the pluripotent stage to terminally differentiated cells and could not maintain themselves as pluripotent without 3i. Therefore, we removed 3i from the medium to determine whether (+)/(+) iPSCs remained pluripotent without 3i. Interestingly, the (+)/(+) iPSCs lost expression of *Nanog* and *Oct3/4* after the 3i withdrawal, while expression of these genes was significantly maintained in

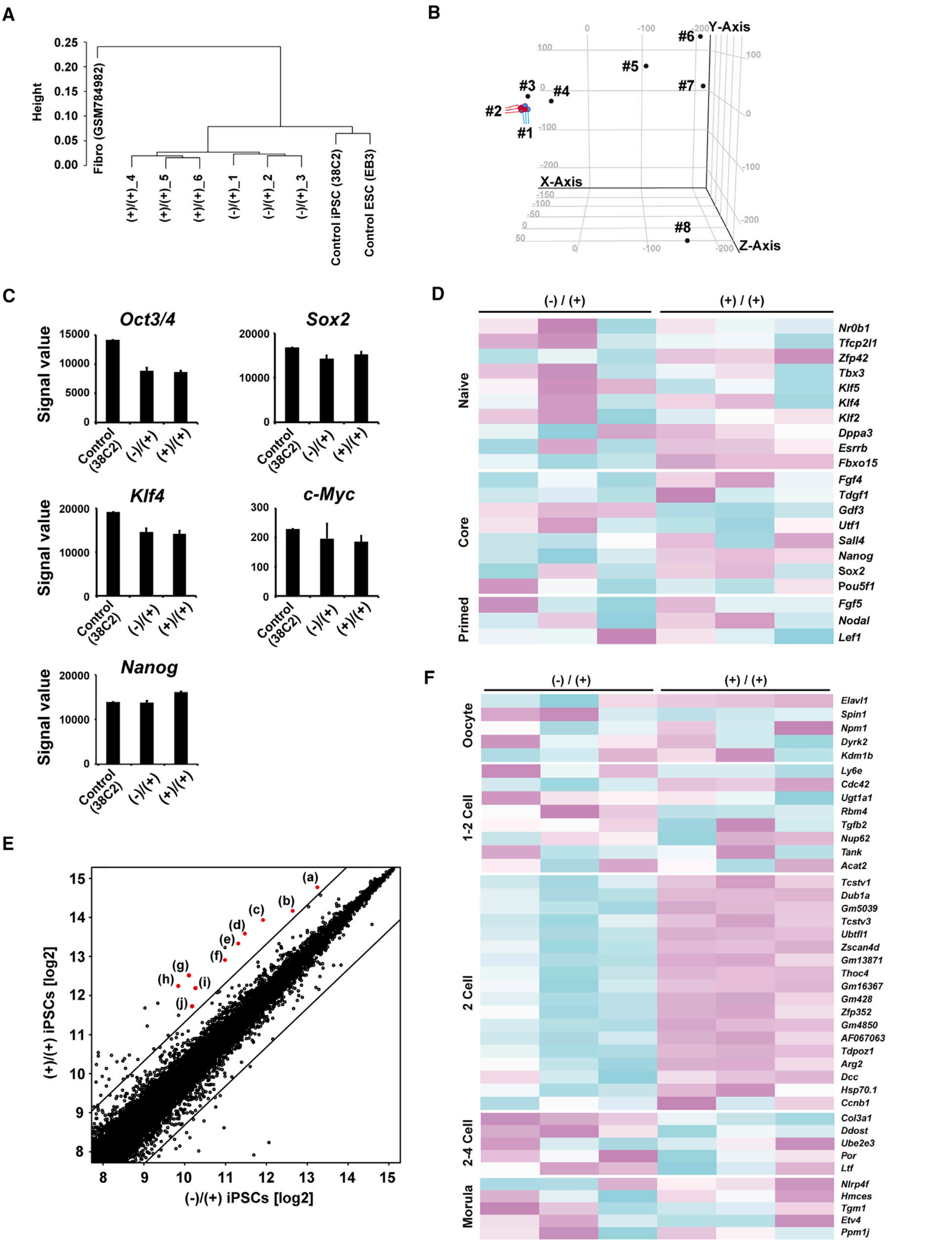

(legend on next page)

the (−)/(+) iPSCs without 3i (Figures 2H and S4). These results suggested that the pluripotency of the (+)/(+) iPSCs could not be maintained in the absence of 3i.

Although the (−)/(+) and (+)/(+) iPSCs exhibited similar characteristics during the pluripotent stage in the presence of 3i (and LIF), these differentiation potentials (e.g., removal of 3i and glial differentiation) were apparently different even though they were derived from the same fibroblasts.

Increased Expression of 2-Cell Stage-Specific Genes in iPSCs Established with 3i during the Reprogramming Period

To explore the difference in global gene expression between the (−)/(+) and (+)/(+) iPSCs that contributed to these distinct differentiation potentials, we performed a microarray analysis using undifferentiated (−)/(+) and (+)/(+) iPSCs along with fibroblasts, ESCs (EB3), and iPSCs (38C2) maintained without 3i as control samples. Hierarchical clustering analysis showed that three independent clones of the (−)/(+) and (+)/(+) iPSCs were clustered into two distinct groups, but they highly resembled each other compared with the fibroblasts or conventional ESCs/iPSCs (Figure 3A). To exclude the possibility that undifferentiated (+)/(+) iPSCs with fast and efficient neural differentiation potentials could be clustered closer to NSCs than (−)/(+) iPSCs in the global gene expression pattern, we performed principal-component analysis (PCA) by using the above samples alongside neurospheres derived from the E14.5 murine embryonic striatum, neurospheres derived from conventional ESCs, and neurospheres derived from conventional iPSCs. PCA showed a similar tendency to that observed in the hierarchical clustering analysis and revealed that the (+)/(+) iPSCs were located much closer to the (−)/(+) iPSCs or conventional ESCs/iPSCs than to the neurospheres (Figure 3B). Then, we concluded that the (+)/(+) iPSCs were in an undifferentiated state with a fast and efficient neural differentiation potential and not biased to a neurally differentiated state like the NSCs.

Next, we compared gene expression between the (−)/(+) (clone 1–3) and (+)/(+) (clone 4–6) iPSCs to explore the key factor driving the difference in their differentiation potentials. As expected, the expression levels of the representative core, naive, and primed pluripotent marker genes were similar between the two groups (Figures 3C and 3D). Gene ontology analysis highlighted biological functions for the differentially expressed genes (DEGs) (Table S3), and scatterplots revealed DEGs (>2.5-fold change, t test, $p < 0.05$, average signal value >500; Figure 3E). Interestingly, 12 highly expressed DEGs (>2.5-fold change, t test, $p < 0.05$, signal value [log2] >9) contained 10 genes that were specifically related to 2-cell embryos, including *Zscan4*, *Eif1a/Eif1a-like*, *Dub1*, and *Gm428* (Amano et al., 2013; Dan et al., 2013). Only two genes (*BC080696* and AA645497) were not 2-cell-related genes, which are not annotated genes. We analyzed representative genes expressed in every early developmental stage, and 2-cell genes were specifically expressed (Figure 3F). These data suggest that the (+)/(+) iPSCs are similar to 2-cell stage cells in global gene expression compared with the (−)/(+) iPSCs.

Differentiation Potential of (+)/(+) iPSCs during the Reprogramming Period with the 3i Chemicals

Based on the results of the microarray analysis, which showed that the (+)/(+) iPSCs had higher 2-cell stage gene expression than the (−)/(+) iPSCs, we performed detailed examinations of the expression of 2-cell stage genes in the (+)/(+) iPSCs by qRT-PCR and immunocytochemistry. The expression levels of the 2-cell stage genes (*Zscan4*, *muERV-L*, *Tcstv1*, and *Eif1a/Eif1a-like*) were significantly increased in the (+)/(+) iPSCs compared with the (−)/(+) iPSCs or control iPSCs (Figure 4A). An increased number of Zscan4-positive cells in the (+)/(+) iPSC group was confirmed by immunocytochemistry (Figures 4B, 4C, and S5). These findings suggested that addition of 3i during

Figure 3. The Differences in Global Gene Expression between (−)/(+) and (+)/(+) iPSCs
(A) Hierarchical cluster analysis based on global gene expression of indicated samples. Microarray data of this study and our previous study (GEO: GSE31725) were collectively normalized using the MAS 5.0 algorithm in every analysis.
(B) Principal-component analysis of gene expression data. Samples are as follows: nos. 1 and 3, clones of the (−)/(+) iPSCs; nos. 2 and 3, clones of the (+)/(+) iPSCs; no. 3, the clone of the ESCs; no. 4, the clone of conventional iPSC derived from a *Nanog-GFP* mouse; no. 5, the clone of the ESC-derived neurospheres; no. 6, the clone of the iPSC-derived neurospheres; no. 7, the clone of the E14 embryo ganglionic eminence-derived neurospheres; no. 8, the clone of the fibroblasts. Gene expression data for nos. 3–8 clones obtained in our previous study (GEO: GSE31725) were used for comparison.
(C) Expression of pluripotent markers. Signal intensities were determined by microarray analysis (n = 3).
(D) Heatmap showing the expression levels for genes that are associated with naive, core, and primed pluripotency.
(E) Scatterplots comparing global gene expression patterns between (−)/(+) and (+)/(+) iPSCs. Highly expressed 2-cell embryo- or *Zscan4*-related genes are shown as red dots. (a) *Zscan4*, (b) *Eif1a/Eif1a-like*, (c) *Gm13040*, (d) AF067061, (e) *Dub1*, (f) *Gm8300*, (g) *LOC639910 Gm13871*, (h) *Tcstv1*, (i) *Gm428*, (j) *Gm18371*, and (k) *Gm428*.
(F) Heatmap showing the expression levels of genes that are specifically expressed in early embryonic stages.
Error bars represent mean ± SD from three independent experiments (n = 3).

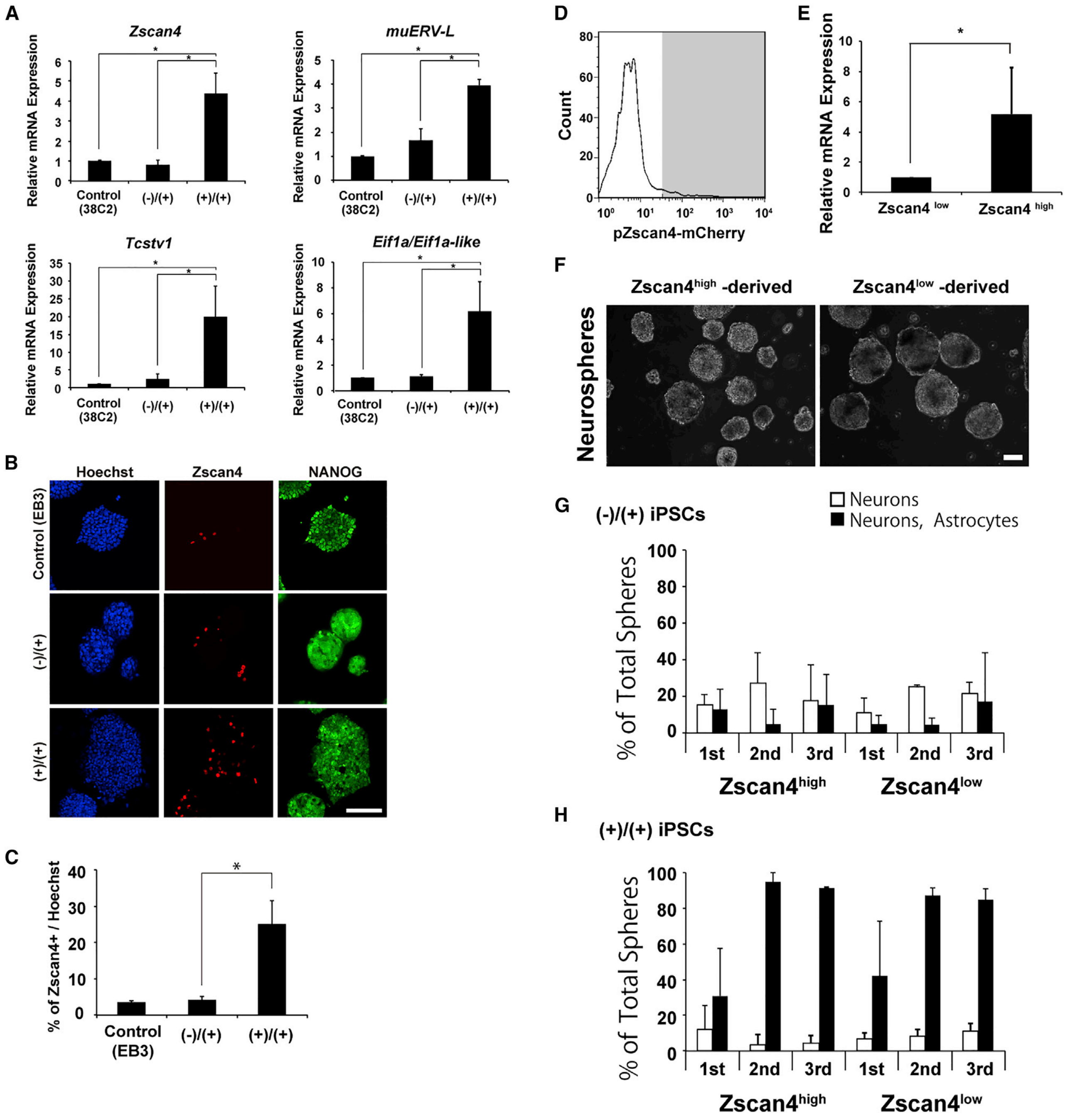

Figure 4. Expression of Genes Associated with the 2-Cell Stage in (−)/(+) and (+)/(+) iPSCs

(A) qRT-PCR analysis of 2-cell-related genes in (−)/(+) and (+)/(+) iPSCs (n = 3; $*p < 0.05$). 38C2 represents conventional iPSCs.

(B) Representative immunocytochemistry images of iPSCs. Nanog expression is indicated by the GFP fluorescence in (−)/(+) and (+)/(+) iPSCs, and the Nanog protein in ESCs is immunostained. Scale bar, 100 μm.

(C) Quantitative analysis of Zscan4-positive cells in (−)/(+) and (+)/(+) iPSCs (n = 3; $*p < 0.05$). ESCs were used as a control.

(D) Zscan4 high or low cells were sorted using a BD FACSAria III cell sorter.

(E) Expression of *Zscan4* in sorted cells was verified by qRT-PCR (n = 3; $*p < 0.05$).

(F) Representative images of Zscan4^{high} iPSC- and Zscan4^{low} iPSC-derived neurospheres. Scale bar, 100 μm.

(G and H) Differentiation efficiency of the first, second, and third neurospheres derived from Zscan4^{high} and Zscan4^{low} iPSCs. The results for the (−)/(+) iPSCs (G) and (+)/(+) iPSCs (H) are shown. The frequency of colonies consisting of neurons (βIII-tubulin) and/or astrocytes (GFAP) was evaluated by immunocytochemistry and is presented as the percentage of positive colonies (n = 3).

the reprogramming period, not the maintenance period, enhanced the gene expression pattern of 2-cell stage iPSCs at the mRNA and protein levels.

It has been reported that a small fraction of cells that express 2-cell stage genes exist among undifferentiated pluripotent cells (Falco et al., 2007; Macfarlan et al., 2012), even in conventional culture conditions. We next sought to determine whether these 2-cell stage gene-positive cells in the (−)/(+) PSC group were identical to the rapidly differentiating cells observed in the (+)/(+) iPSC group. When both populations were identical, the 3i chemicals during the reprogramming period simply increased the 2-cell stage gene-positive cells in the (+)/(+) iPSC population.

To characterize the cells with high 2-cell stage gene expression, we isolated these cells using the pZscan4-mCherry reporter, as a previous report showed that Zscan4-positive cells highly expressed other 2-cell genes and showed similar oscillations in the expression of these genes (Amano et al., 2013; Zalzman et al., 2010). A plasmid containing the pZscan4-mCherry reporter and a PGK-Neomycin resistance gene was transiently transfected into (−)/(+) and (+)/(+) iPSCs; these cells were selected with G418 and sorted into Zscan4-mCherry-high (Zscan4^{high}) or *Zscan4*-mCherry-low (Zscan4^{low}) populations by fluorescence-activated cell sorting (Figure 4D). qRT-PCR analysis revealed that the Zscan4^{high} cells expressed approximately 5-fold more *Zscan4* that the Zscan4^{low} cells (Figure 4E). Then, we generated neurospheres using Zscan4^{high} or Zscan4^{low} cells from the (−)/(+) iPSCs. The frequencies of gliogenic sphere formation from mature NSCs were relatively low and were similar between the Zscan4^{high} and Zscan4^{low} cells, while the Zscan4^{low} cells from the (+)/(+) iPSCs mainly give rise to mature gliogenic neurospheres, as did the Zscan4^{high} cells from the (+)/(+) iPSCs (Figures 4F–4H). These data suggested that Zscan4^{high} cells were present in (−)/(+) iPSC group but that the Zscan4^{high} cells were distinct from those of the (+)/(+) iPSCs. Presumably, this difference was already determined during the reprogramming period with 3i chemicals. An increased number of Zscan4^{high} cells in the (+)/(+) iPSC group suggested that the (+)/(+) iPSCs were stable at a distinct status, where 2-cell stage markers were expressed, compared with the (−)/(+) iPSCs.

DISCUSSION

In this study, we showed that exposure to 3i chemicals during a reprogramming period altered the differentiation potentials of generated iPSCs and that these potentials could not be changed by 3i exposure once reprogramming was completed. It remains controversial whether these chemicals, including FGF-MAPK cascade/GSK3β inhibitors, which induce ground-state pluripotency in PSCs, can improve the differentiation potentials of PSCs (Chan et al., 2013; Gafni et al., 2013; Takashima et al., 2014; Theunissen et al., 2014; Valamehr et al., 2014). Although many chemical compounds have been reported for facilitating reprogramming or sustaining pluripotency (Huangfu et al., 2008; Li et al., 2010; Mali et al., 2010; Silva et al., 2008; Zhu et al., 2010), it is not fully known whether iPSCs generated with these chemicals had similar differentiation potentials compared with conventional iPSCs in both mice and humans.

Before the 3i chemicals were discovered to maintain PSC pluripotency (Ying et al., 2008), ESCs with chimera-forming ability had been established only in mice (Evans and Kaufman, 1981) because it was very difficult to maintain the pluripotent states of cells from other species. However, using 3i, ESCs have been established from rats (Buehr et al., 2008), indicating that these ESCs could not be maintained without the support of 3i. Even in the murine cells that we have analyzed in this study, the number of iPSC colonies was significantly increased with 3i during the reprogramming period. Therefore, it is likely that cells that cannot be reprogrammed and maintained in pluripotency without 3i chemicals correspond to the increased population in 3i-treated reprogramming. Our data showing that (+)/(+) iPSCs quickly lost their pluripotency without 3i (Figure S4) might support this hypothesis.

We previously reported that fibroblasts can be directly converted into NSCs through partially reprogrammed intermediates by the brief expression of Yamanaka factors. Furthermore, we reported that these induced NSCs (diNSCs) are highly gliogenic, even at an early passage, whereas ESC-derived neurospheres generate mostly neurons and a few astrocytes. These observations suggest that diNSCs can develop into mature NSCs more rapidly along with NSCs derived from mouse brains (Matsui et al., 2012). It has also been reported that hepatocytes derived from partially reprogrammed intermediate cells mature more efficiently than those derived from iPSCs (Zhu et al., 2014). These observations also suggested that bypassing the iPSC state can induce rapidly differentiating cells because clonal isolation of iPSCs may result in loss of these cells due to long-term exposure to the severe selective culture condition.

Here, we have clearly shown that there are distinct differentiation/maturation potentials between (+)/(+) and (−)/(+) iPSCs that can be determined by the culture condition during a reprogramming period, not during a maintenance period. Another study that examined early differentiation using EB formation has shown that the differentiation potential of iPSCs can be changed by SB431542, ascorbic acid, thiazovivin, and PD0325901

during the reprogramming period (Park et al., 2015). Interestingly, our results have also shown that differentiation from (+)/(+) iPSCs is accelerated toward all three germ layers in comparison with (–)/(+) iPSCs. Several previous reports have shown that epigenetic memories influence biased and insufficient differentiation potentials in PSCs (Kim et al., 2010b; Koyanagi-Aoi et al., 2013; Polo et al., 2010). Moreover, FGF4-MAPK/GSK3β inhibitors affect the global methylation profile (Leitch et al., 2013) in PSCs, and it has been suggested that treatment with 3i during a reprogramming period might induce irreversible epigenetic changes in iPSCs.

We found that the expression of 2-cell stage genes including *Zscan4* was significantly different between (+)/(+) and (–)/(+) iPSCs. Although one previous report demonstrated that the addition of 2i to established iPSCs promotes the expression of 2-cell genes (Cerulo et al., 2014), the Zscan4 expression levels in both (–)/(+) iPSCs and control ES/iPSCs were similar in this study. Our data also showed that the amount of Zscan4 in each iPSC did not affect the iPSC differentiation potential; these characteristics had already been determined during the reprogramming period. This finding seems to be compatible with the previous report mentioning that forced expression of Zscan4 during reprogramming improved differentiation potentials of iPSCs as evaluated by the tetraploid complementation assay (Jiang et al., 2013). These 2-cell stage genes are not the markers that predict the rapid differentiation potential of each iPSC. Identification of these preventative markers will be useful for isolating rapidly differentiating iPSCs from conventional iPSC clones. However, it is significant that (+)/(+) iPSCs are stable at a distinct stage with higher expression levels of 2-cell stage genes compared with (–)/(+) iPSCs. However, DNA were hypermethylated in (+)/(+) iPSCs. In the development of the early embryo, the total amount of genomic methylation is gradually decreased toward the blastocyst stage (Wu and Zhang, 2014). These data suggested that the (+)/(+) iPSCs, which are hypermethylated, are at a younger stage compared with (–)/(+) iPSCs. Transient histone acetylation in heterochromatin as well as DNA demethylation have reported to occur during the activation of 2-cell stage genes in ESCs (Akiyama et al., 2015), although it remains unclear whether these epigenetic modifications influence accelerated differentiation potentials of (+)/(+) iPSCs.

In summary, our study demonstrated that the use of 3i chemicals—PD184352, CHIR99021 and SU5402—during a reprogramming period induces accelerated differentiation/maturation potentials in murine iPSCs that are stable at a distinct state with increased expression of 2-cell stage markers and highly methylated profile that is likely to be primitive compared with those without 3i chemicals. Whether naive human iPSCs have a superior differentiation ability to primed hiPSCs remains controversial in the field of regenerative medicine. Our observations may support the hypothesis that PSCs that are stable at a distinct stage have an accelerated and non-biased differentiation potential compared with those at a lower stage.

EXPERIMENTAL PROCEDURES

The teratoma assay was performed in accordance with the Guidelines for the Care and Use of Laboratory Animals of Keio University (assurance no. 09169). Chimera formation was performed in accordance with the Guidelines for the Care and Use of Laboratory Animals of Juntendo University (assurance no. 300083).

iPSC Generation

Murine iPSC generation was performed as described previously with slight modifications. In brief, 3.6×10^6 Plat-E packaging cells were seeded per 100-mm dish, and, the next day, these cells were transfected with pMXs retroviral vectors (encoding *Oct 3/4*, *Klf4*, *Sox2*, and *c-Myc*; Addgene) using the Fugene 6 transfection reagent (Roche). At this time, we also transfected dsRed to ascertain silencing of the transgenes after colony formation. Virus-containing medium supplemented with 4 μg/mL Polybrene was added to 8×10^5 MEFs, and MEFs were re-seeded on SNL feeder cells 4 days after infection. Approximately 3 weeks after the transduction, iPSC colonies were picked and expanded. (+)/(+) iPSCs were treated with 3i beginning 2 days after the transduction, while (–)/(+) iPSCs were treated with 3i after colony isolation (approximately 3 weeks after the transduction).

Quantification of Reprogramming Efficiency

For quantification of the reprogramming efficiency of murine cells, 1×10^5 transduced MEFs were re-seeded into six-well plates (Nunc). Twenty-one days after the transduction, the number of *Nanog*-EGFP$^+$ and dsRed$^-$ ESC-like colonies was counted.

In Vitro Neural Differentiation of iPSCs

iPSCs were dissociated with TrypLE Select (Life Technologies) and suspended at a density of 1×10^5 cells/mL in a serum-free media hormone mix (MHM) medium supplemented with B27 (Life Technologies), 1,000 U/mL LIF (Nakalai Tesque) and 20 ng/mL FGF2 (PeproTech) to form neurospheres. Neurospheres were passaged repeatedly every 5–7 days by enzymatic dissociation into single cells in the same culture medium. For sphere formation assays, neurospheres were dissociated and plated at 1×10^4 cells/mL in 96-well ultra-low plates, and the number of formed spheres per well was counted after 7 days. For neural differentiation assays, neurospheres were seeded on poly-L-ornithine and fibronectin-coated chambers and cultured for 7 days in MHM medium supplemented with B27 and 1% FBS.

Global Gene Expression Analysis

Total RNA purification was performed using TRIzol reagent and a RNeasy Mini Kit (QIAGEN). Global gene expression analysis was performed using Affymetrix Gene Chip Mouse Genome 430 2.0 as per the manufacturer's protocol. The microarray signals were

quantified using the MAS 5.0 method. Hierarchical clustering and heatmaps were designed using the R package and Bioconductor. Distances (=1 – Spearman's correlation coefficient) were calculated using the R package. PCA and scatterplots were designed using GeneSpring GX. Gene ontology analysis was performed through the use of QIAGEN's Ingenuity Pathway Analysis (QIAGEN Redwood City, www.qiagen.com/ingenuity).

ACCESSION NUMBERS

The accession number for the microarray data in this paper is NCBI GEO: GSE110515. The MBD-seq data reported are available in the DDBJ databases under the accession number DRA007928.

SUPPLEMENTAL INFORMATION

Supplemental Information includes Supplemental Experimental Procedures, five figures, and five tables and can be found with this article online at https://doi.org/10.1016/j.stemcr.2018.12.018.

AUTHOR CONTRIBUTIONS

K.N., T. Shiga, H.O., and W.A. designed the experiments, analyzed the data, and wrote the manuscript. K.N. and T. Shiga performed most of the experiments and analyzed the data. T.A. and M.S.H.K. designed and made the reporter construct. S.S. performed fluorescence-activated cell sorting analysis. E.N. and N.T performed chimera formation experiments. T. Sasaki analyzed MBD-seq data. All the authors read and approved the final version of the manuscript. H.O. is a founding scientist and a paid member of the Scientific Advisory Board of San Bio and K Pharma. All other authors declare that they have no competing interests.

ACKNOWLEDGMENTS

We thank Drs. Kyoko Miura and Ikuko Koya for technical advice. We also thank Drs. Keisuke Okita and Shinya Yamanaka for the *Nanog*-GFP mice. This work was partly supported by a grant from the Japan Society for the Promotion of Science (JSPS). K.N. is supported by a Research Fellowship for Young Scientists from JSPS. T. Shiga and W.A. are supported by a MEXT-Supported Program for the Strategic Research Foundation at Private Universities. H.O. is a founding scientist and a paid member of the Scientific Advisory Board of San Bio and K Pharma.

Received: July 26, 2017
Revised: December 29, 2018
Accepted: December 30, 2018
Published: January 31, 2019

REFERENCES

Akamatsu, W., DeVeale, B., Okano, H., Cooney, A.J., and van der Kooy, D. (2009). Suppression of Oct4 by germ cell nuclear factor restricts pluripotency and promotes neural stem cell development in the early neural lineage. J. Neurosci. *29*, 2113–2124.

Akiyama, T., Xin, L., Oda, M., Sharov, A.A., Amano, M., Piao, Y., Cadet, J.S., Dudekula, D.B., Qian, Y., Wang, W., et al. (2015). Transient bursts of Zscan4 expression are accompanied by the rapid derepression of heterochromatin in mouse embryonic stem cells. DNA Res. *22*, 307–318.

Amano, T., Hirata, T., Falco, G., Monti, M., Sharova, L.V., Amano, M., Sheer, S., Hoang, H.G., Piao, Y., Stagg, C.A., et al. (2013). Zscan4 restores the developmental potency of embryonic stem cells. Nat. Commun. *4*, 1966.

Buehr, M., Meek, S., Blair, K., Yang, J., Ure, J., Silva, J., McLay, R., Hall, J., Ying, Q.L., and Smith, A. (2008). Capture of authentic embryonic stem cells from rat blastocysts. Cell *135*, 1287–1298.

Cerulo, L., Tagliaferri, D., Marotta, P., Zoppoli, P., Russo, F., Mazio, C., DeFelice, M., Ceccarelli, M., and Falco, G. (2014). Identification of a novel gene signature of ES cells self-renewal fluctuation through system-wide analysis. PLoS One *9*, e83235.

Chan, Y.S., Göke, J., Ng, J.H., Lu, X., Gonzales, K.A.U., Tan, C.P., Tng, W.Q., Hong, Z.Z., Lim, Y.S., and Ng, H.H. (2013). Induction of a human pluripotent state with distinct regulatory circuitry that resembles preimplantation epiblast. Cell Stem Cell *13*, 663–675.

Choi, J., Huebner, A.J., Clement, K., Walsh, R.M., Savol, A., Lin, K., Gu, H., Di Stefano, B., Brumbaugh, J., Kim, S.Y., et al. (2017). Prolonged Mek1/2 suppression impairs the developmental potential of embryonic stem cells. Nature *10*, 219–223.

Conti, L., and Cattaneo, E. (2010). Neural stem cell systems: physiological players or in vitro entities? Nat. Rev. Neurosci. *11*, 176–187.

Dan, J., Li, M., Yang, J., Li, J., Okuka, M., Ye, X., and Liu, L. (2013). Roles for Tbx3 in regulation of two-cell state and telomere elongation in mouse ES cells. Sci. Rep. *3*, 3492.

Duggal, G., Warrier, S., Ghimire, S., Broekaert, D., Van der Jeught, M., Lierman, S., Deroo, T., Peelman, L., Van Soom, A., Cornelissen, R., et al. (2015). Alternative routes to induce naïve pluripotency in human embryonic stem cells. Stem Cells *33*, 2686–2698.

Evans, M.J., and Kaufman, M.H. (1981). Establishment in culture of pluripotential cells from mouse embryos. Nature *292*, 154–156.

Falco, G., Lee, S.L., Stanghellini, I., Bassey, U.C., Hamatani, T., and Ko, M.S.H. (2007). Zscan4: a novel gene expressed exclusively in late 2-cell embryos and embryonic stem cells. Dev. Biol. *307*, 539–550.

Gafni, O., Weinberger, L., Mansour, A.A., Manor, Y.S., Chomsky, E., Ben-Yosef, D., Kalma, Y., Viukov, S., Maza, I., Zviran, A., et al. (2013). Derivation of novel human ground state naive pluripotent stem cells. Nature *504*, 282–286.

Hitoshi, S., Alexson, T., Tropepe, V., Donoviel, D., Elia, A.J., Nye, J.S., Conlon, R.A., Mak, T.W., Bernstein, A., and van der Kooy, D. (2002). Notch pathway molecules are essential for the maintenance, but not the generation, of mammalian neural stem cells. Genes Dev. *16*, 846–858.

Hitoshi, S., Seaberg, R.M., Koscik, C., Alexson, T., Kusunoki, S., Kanazawa, I., Tsuji, S., and van der Kooy, D. (2004). Primitive neural stem cells from the mammalian epiblast differentiate to definitive neural stem cells under the control of Notch signaling. Genes Dev. *18*, 1806–1811.

Honda, A., Hatori, M., Hirose, M., Honda, C., Izu, H., Inoue, K., Hirasawa, R., Matoba, S., Togayachi, S., Miyoshi, H., et al. (2013).

Naive-like conversion overcomes the limited differentiation capacity of induced pluripotent stem cells. J. Biol. Chem. *288*, 26157–26166.

Huangfu, D., Maehr, R., Guo, W., Eijkelenboom, A., Snitow, M., Chen, A.E., and Melton, D.A. (2008). Induction of pluripotent stem cells by defined factors is greatly improved by small-molecule compounds. Nat. Biotechnol. *26*, 795–797.

Jiang, J., Lv, W., Ye, X., Wang, L., Zhang, M., Yang, H., Okuka, M., Zhou, C., Zhang, X., and Liu, L. (2013). Zscan4 promotes genomic stability during reprogramming and dramatically improves the quality of iPS cells as demonstrated by tetraploid complementation. Cell Res. *23*, 92–106.

Jin, Z.B., Okamoto, S., Osakada, F., Homma, K., Assawachananont, J., Hirami, Y., Iwata, T., and Takahashi, M. (2011). Modeling retinal degeneration using patient-specific induced pluripotent stem cells. PLoS One *6*, e17084.

Kim, D.S., Lee, J.S., Leem, J.W., Huh, Y.J., Kim, J.Y., Kim, H.S., Park, I.H., Daley, G.Q., Hwang, D.Y., and Kim, D.W. (2010a). Robust enhancement of neural differentiation from human ES and iPS cells regardless of their innate difference in differentiation propensity. Stem Cell Rev. *6*, 270–281.

Kim, K., Doi, A., Wen, B., Ng, K., Zhao, R., Cahan, P., Kim, J., Aryee, M.J., Ji, H., Ehrlich, L.I.R., et al. (2010b). Epigenetic memory in induced pluripotent stem cells. Nature *467*, 285–290.

Koyanagi-Aoi, M., Ohnuki, M., Takahashi, K., Okita, K., Noma, H., Sawamura, Y., Teramoto, I., Narita, M., Sato, Y., Ichisaka, T., et al. (2013). Differentiation-defective phenotypes revealed by large-scale analyses of human pluripotent stem cells. Proc. Natl. Acad. Sci. U S A *110*, 20569–20574.

Leitch, H.G., McEwen, K.R., Turp, A., Encheva, V., Carroll, T., Grabole, N., Mansfield, W., Nashun, B., Knezovich, J.G., Smith, A., et al. (2013). Naive pluripotency is associated with global DNA hypomethylation. Nat. Struct. Mol. Biol. *20*, 311–316.

Li, R., Liang, J., Ni, S., Zhou, T., Qing, X., Li, H., He, W., Chen, J., Li, F., Zhuang, Q., et al. (2010). A mesenchymal-to-epithelial transition initiates and is required for the nuclear reprogramming of mouse fibroblasts. Cell Stem Cell *7*, 51–63.

Ma, X., Duan, Y., Tschudy-Seney, B., Roll, G., Behbahan, I.S., Ahuja, T.P., Tolstikov, V., Wang, C., McGee, J., Khoobyari, S., et al. (2013). Highly efficient differentiation of functional hepatocytes from human induced pluripotent stem cells. Stem Cells Transl. Med. *2*, 409–419.

Macfarlan, T.S., Gifford, W.D., Driscoll, S., Lettieri, K., Rowe, H.M., Bonanomi, D., Firth, A., Singer, O., Trono, D., and Pfaff, S.L. (2012). Embryonic stem cell potency fluctuates with endogenous retrovirus activity. Nature *487*, 57–63.

Mali, P., Chou, B.K., Yen, J., Ye, Z., Zou, J., Dowey, S., Brodsky, R.A., Ohm, J.E., Yu, W., Baylin, S.B., et al. (2010). Butyrate greatly enhances derivation of human induced pluripotent stem cells by promoting epigenetic remodeling and the expression of pluripotency-associated genes. Stem Cells *28*, 713–720.

Marks, H., Kalkan, T., Menafra, R., Denissov, S., Jones, K., Hofemeister, H., Nichols, J., Kranz, A., Stewart, A.F., Smith, A., et al. (2012). The transcriptional and epigenomic foundations of ground state pluripotency. Cell *149*, 590–604.

Matsui, T., Takano, M., Yoshida, K., Ono, S., Fujisaki, C., Matsuzaki, Y., Toyama, Y., Nakamura, M., Okano, H., and Akamatsu, W. (2012). Neural stem cells directly differentiated from partially reprogrammed fibroblasts rapidly acquire gliogenic competency. Stem Cells *30*, 1109–1119.

Nagoshi, N., and Okano, H. (2017). Applications of induced pluripotent stem cell technologies in spinal cord injury. J. Neurochem. *141*, 1–13.

Naka, H., Nakamura, S., Shimazaki, T., and Okano, H. (2008). Requirement for COUP-TFI and II in the temporal specification of neural stem cells in CNS development. Nat. Neurosci. *11*, 1014–1023.

Nelson, T.J., Martinez-Fernandez, A., Yamada, S., Perez-Terzic, C., Ikeda, Y., and Terzic, A. (2009). Repair of acute myocardial infarction by human stemness factors induced pluripotent stem cells. Circulation *120*, 408–416.

Numasawa-Kuroiwa, Y., Okada, Y., Shibata, S., Kishi, N., Akamatsu, W., Shoji, M., Nakanishi, A., Oyama, M., Osaka, H., Inoue, K., et al. (2014). Involvement of ER stress in dysmyelination of Pelizaeus-Merzbacher disease with PLP1 missense mutations shown by iPSC-derived oligodendrocytes. Stem Cell Rep. *2*, 648–661.

Okada, Y., Matsumoto, A., Shimazaki, T., Enoki, R., Koizumi, A., Ishii, S., Itoyama, Y., Sobue, G., and Okano, H. (2008). Spatiotemporal recapitulation of central nervous system development by murine embryonic stem cell-derived neural stem/progenitor cells. Stem Cells *26*, 3086–3098.

Okita, K., Ichisaka, T., and Yamanaka, S. (2007). Generation of germline-competent induced pluripotent stem cells. Nature *448*, 313–317.

Osafune, K., Caron, L., Borowiak, M., Martinez, R.J., Fitz-Gerald, C.S., Sato, Y., Cowan, C.A., Chien, K.R., and Melton, D.A. (2008). Marked differences in differentiation propensity among human embryonic stem cell lines. Nat. Biotechnol. *26*, 313–315.

Park, H.S., Hwang, I., Choi, K.A., Jeong, H., Lee, J.Y., and Hong, S. (2015). Generation of induced pluripotent stem cells without genetic defects by small molecules. Biomaterials *39*, 47–58.

Polo, J.M., Liu, S., Figueroa, M.E., Kulalert, W., Eminli, S., Tan, K.Y., Apostolou, E., Stadtfeld, M., Li, Y., Shioda, T., et al. (2010). Cell type of origin influences the molecular and functional properties of mouse induced pluripotent stem cells. Nat. Biotechnol. *28*, 848–855.

Shi, Y., Inoue, H., Wu, J.C., and Yamanaka, S. (2017). Induced pluripotent stem cell technology: a decade of progress. Nat. Rev. Drug Discov. *16*, 115–130.

Shimamoto, R., Amano, N., Ichisaka, T., Watanabe, A., Yamanaka, S., and Okita, K. (2014). Generation and characterization of induced pluripotent stem cells from aid-deficient mice. PLoS One *9*, 1–13.

Silva, J., Barrandon, O., Nichols, J., Kawaguchi, J., Theunissen, T.W., and Smith, A. (2008). Promotion of reprogramming to ground state pluripotency by signal inhibition. PLoS Biol. *6*, e253.

Takahashi, K., and Yamanaka, S. (2006). Induction of pluripotent stem cells from mouse embryonic and adult fibroblast cultures by defined factors. Cell *126*, 663–676.

Takashima, Y., Guo, G., Loos, R., Nichols, J., Ficz, G., Krueger, F., Oxley, D., Santos, F., Clarke, J., Mansfield, W., et al. (2014). Resetting transcription factor control circuitry toward ground-state pluripotency in human. Cell *158*, 1254–1269.

Theunissen, T., Powell, B., Wang, H., Mitalipova, M., Faddah, D., Reddy, J., Fan, Z., Maetzel, D., Ganz, K., and Shi, L. (2014). Systematic identification of culture conditions for induction and maintenance of naive human pluripotency. Cell Stem Cell *15*, 471–487.

Tornero, D., Wattananit, S., Madsen, M.G., Koch, P., Wood, J., Tatarishvili, J., Mine, Y., Ge, R., Monni, E., Devaraju, K., et al. (2013). Human induced pluripotent stem cell-derived cortical neurons integrate in stroke-injured cortex and improve functional recovery. Brain *136*, 3561–3577.

Tropepe, V., Hitoshi, S., Sirard, C., Mak, T.W., Rossant, J., and van der Kooy, D. (2001). Direct neural fate specification from embryonic stem cells: a primitive mammalian neural stem cell stage acquired through a default mechanism. Neuron *30*, 65–78.

Valamehr, B., Robinson, M., Abujarour, R., Rezner, B., Vranceanu, F., Le, T., Medcalf, A., Lee, T.T., Fitch, M., Robbins, D., et al. (2014). Platform for induction and maintenance of transgene-free hiPSCs resembling ground state pluripotent stem cells. Stem Cell Rep. *2*, 366–381.

Wu, H., and Zhang, Y. (2014). Reversing DNA methylation: mechanisms, genomics, and biological functions. Cell *156*, 45–68.

Ying, Q.L., Wray, J., Nichols, J., Batlle-Morera, L., Doble, B., Woodgett, J., Cohen, P., and Smith, A. (2008). The ground state of embryonic stem cell self-renewal. Nature *453*, 519–523.

Zalzman, M., Falco, G., Sharova, L.V., Nishiyama, A., Thomas, M., Lee, S.L., Stagg, C.A., Hoang, H.G., Yang, H.T., Indig, F.E., et al. (2010). Zscan4 regulates telomere elongation and genomic stability in ES cells. Nature *464*, 858–863.

Zhu, S., Li, W., Zhou, H., Wei, W., Ambasudhan, R., Lin, T., Kim, J., Zhang, K., and Ding, S. (2010). Reprogramming of human primary somatic cells by OCT4 and chemical compounds. Cell Stem Cell *7*, 651–655.

Zhu, S., Rezvani, M., Harbell, J., Mattis, A.N., Wolfe, A.R., Benet, L.Z., Willenbring, H., and Ding, S. (2014). Mouse liver repopulation with hepatocytes generated from human fibroblasts. Nature *508*, 93–97.

Stem Cell Reports, Volume *12*

Supplemental Information

Induced Pluripotent Stem Cells Reprogrammed with Three Inhibitors Show Accelerated Differentiation Potentials with High Levels of 2-Cell Stage Marker Expression

Koji Nishihara, Takahiro Shiga, Eri Nakamura, Tomohiko Akiyama, Takashi Sasaki, Sadafumi Suzuki, Minoru S.H. Ko, Norihiro Tada, Hideyuki Okano, and Wado Akamatsu

Supplemental Experimental Procedures

Cell culture

Mouse embryonic fibroblasts (MEFs) were derived from E13.5 embryos from homozygous *Nanog*-EGFP transgenic mice (a kind gift from Dr. Okita (and Dr. Yamanaka) of Kyoto University). MEFs were cultured in Dulbecco's modified Eagle's medium (DMEM) supplemented with 10% fetal bovine serum (FBS), L-glutamine, penicillin, and streptomycin. We used MEFs to generate iPSCs between passages 3 and 5. Established iPSCs were cultured in mouse ES cell medium (DMEM containing 15% FBS, 1,000 U/ml of leukemia inhibitory factor (LIF) (Nakalai Tesque), a nonessential amino acid solution, L-glutamine, β-mercaptoethanol, penicillin and streptomycin) on mitomycin-C-treated SNL feeder cells and treated with or without small molecule inhibitors known as 3i (0.8 μM MEK inhibitor PD184352, 2 μM FGF-R inhibitor SU5402 and 3 μM GSK-3β inhibitor CHIR99021). All experiments were conducted at passages 6-18. A previously established murine iPSC clone (38C2) generated from a *Nanog*-GFP mouse was also used as a control iPSCs.

Immunocytochemistry and Alkaline Phosphatase (AP) staining

For immunocytochemical analysis, cells were fixed with 4% paraformaldehyde (PFA) in phosphate buffered saline (PBS) for 25 min. The cells were analyzed by immunofluorescence staining using antibodies to the following proteins: Nanog (1:100, ReproCELL), SSEA-1 (500:1, Abcam), β III-tubulin (1:1000, Sigma), GFAP (1:4000, DAKO). Cells were washed with PBS three times, and incubated with an Alexa Fluor 488- or 555-conjugated secondary antibody (1:500, invitrogen). Nuclei were stained with 10 μg ml-1 Hoechst 33258 (Sigma). Images were acquired with Apotome (Zeiss) or LSM-710 confocal (Zeiss) microscopes. AP staining was performed using the Leukocyte Alkaline Phosphatase Kit (Sigma) following the manufacturer's instruction.

Teratoma Assay

To assess teratoma formation, iPSCs were injected into the testis of 7-week-old SCID mice (Charles River Laboratories). Four weeks after transplantation, tumors were dissected and fixed with 4% PFA in PBS. Paraffin-embedded tissue was sectioned and stained with hematoxylin and eosin (H&E) staining. Images were obtained using an Axio Imager M1 microscope (Zeiss). Teratoma Assay were performed in accordance with the Guidelines for the Care and Use of Laboratory Animals of Keio University (Assurance no. 09169).

RNA Isolation, Reverse Transcription, and Polymerase Chain Reaction (PCR)

Total RNA was purified using TRIzol Reagent (Invitrogen) and RNeasy Kit (Qiagen) according to the manufacturer's protocol with slight modifications. First-strand cDNA was synthesized with the SuperScript III First-strand Synthesis System (Invitrogen).
Quantitative real-time PCR reactions were performed using SYBR Premix Ex Taq II (Takara), and transcript levels were determined using a ABI PRISM Sequence detection System 7900HT or ViiA7 (Applied BioSystems). The amount of mRNA was normalized to β-actin mRNA. The primer sequences used in this study are listed in Table S3.

Fluorescence-Activated Cell Sorting (FACS)

We performed fluorescence-activated cell sorting (FACS) to sort iPSCs using a BD FACSAriaIII cell sorter. iPSCs and feeder cells were dissociated and seeded onto gelatin-coated dishes for 20 - 25 min to exclude feeder cells, and the supernatant was harvested and suspended in a diluted mixture of mouse ES medium and PBS. EB3s (ESCs with no fluorescence) and EB3s transfected with a CAG-RFP reporter were used as control cells.

MBD-sequencing (MBD-seq)

iPSCs were purified with FACS via size gating and fluorescence (*Nanog*-EGFP) to remove feeder fibroblasts. Genomic DNA was sonicated to produce fragments, and methylated DNA was captured with an EpiXplore Methylated DNA Enrichment Kit (Takara) according to the manufacturer's protocol. Sequencing libraries were prepared by using a TruSeq ChIP Sample Prep Kit (Illumina). DNA fragments in the MBD-seq library were sequenced by Illumina GAIIx (Illumina) in single-end 75-bp mode. The obtained reads were mapped to mouse reference sequence (mm9) by the BWA program (version 0.6.1), and the mapped data were analyzed by the MACS program in Avadis NGS 1.3.1 (Strand Life Sciences) to detect MBD binding regions. The gene body including 3 kb upstream of the translational start site was treated as the gene region.

CGH array

CGH array was performed according to the Agilent CGH array protocol (v7.5). Genomic DNA was extracted from iPSCs and MEFs using a Qiagen DNeasy Blood and Tissue kit. The array was measured using a SurePrint G3 mouse CGH 4x80K array (TAKARA BIO, Japan).

Chimera formation

Oocytes were collected from B6.albino female mice (Charles River Laboratories Japan, Kanagawa, Japan)

superovulated by intraperitoneal injection of PMSG (Peamex, Sankyo Lifetech Inc., Tokyo, Japan) followed by hCG (Sigma-Aldrich, St. Louis, Missouri, USA) at an interval of 48h, and then fertilized in vitro with spermatozoa from B6.albino male mice (Charles River Laboratories). Mouse blastocysts were obtained from oocytes fertilized in vitro by culturing them for 3.5 days after fertilization. Mouse iPSCs were trypsinized and directly injected 10~15 cells into the cavity of blastocysts. Injections were performed by micromanipulators (Leica, Wetzlar, Germany) with a PMM-150 FU piezo-impact drive unit (Prime Tech Inc., Ibaragi, Japan) using a blunt-ended, mercury-containing injection pipette. Injected blastocysts were transferred to the uterus of a 2.5-dpc pseudopregnant ICR recipient females (Charles River Laboratories Japan) that were mated to vasectomized ICR male mice (Charles River Laboratories Japan). Chimera formation were performed in accordance with the Guidelines for the Care and Use of Laboratory Animals of Juntendo University (Assurance no. 300083).

Statistical Analysis

Data shown are represented as means ± SEM. Student's t test was used for comparisons of two groups. p Values <0.05 were considered significant.

SUPPLEMENTAL REFERENCES

Fujimori K, Matsumoto T, Kisa F, Hattori N, Okano H, Akamatsu W. (2017). Escape from Pluripotency via Inhibition of TGF-β/BMP and Activation of Wnt Signaling Accelerates Differentiation and Aging in hPSC Progeny Cells. Stem Cell Reports *14*, 1675-1691.

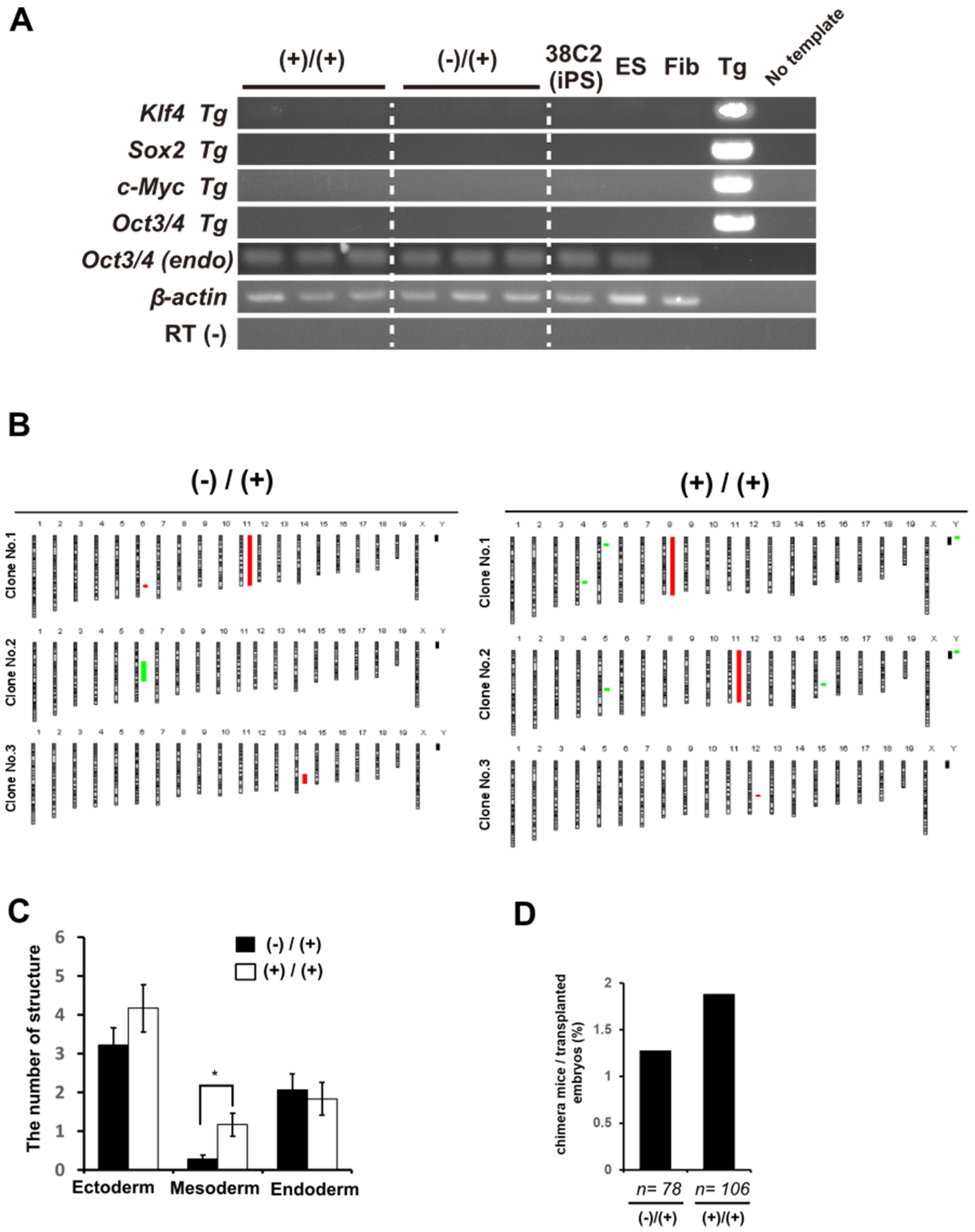

Figure S1. Expression patterns of transgenes in (-)/(+) and (+)/(+) iPSCs (A) Total RNA was isolated from each cell type, and RT-PCR analysis was performed using primers amplifying the transgene transcripts (*Klf4, Sox2, c-Myc, and Oct3/4 Tg*), the coding region of endogenous *Oct3/4* (endo) and *β-actin* (as loading control). Control templates were as follows: 38C2, previously established iPSCs derived from *Nanog*-GFP mouse; ES, EB3 mouse ESCs; Fib, fibroblasts; Tg; pMXs, retroviral vectors of each transgene.(B) Summary of genetic alterations in iPS cell lines based on CGH array. Chromosomal

alteration frequencies are visualized by red lines (amplifications), and green lines represent deletions. (C) Teratomas generated from (-)/(+) and (+)/(+) iPSCs injected into NOD/SCID mice were stained with hematoxylin and eosin. The numbers of differentiated structures from three germ layers (ectoderm: neural epithelium and neural rosettes; mesoderm: cartilage and bone; endoderm: gut-like epithelium with Goblet cells or ciliated structures) were counted from randomly selected 10x visual fields (3 clones of (-)/(+) and (+)/(+) iPSCs, n=6; *, $p < 0.05$).

(D) Graph shows percentage of chimeric mouse at 4 weeks of age in (-)/(+) and (+)/(+) iPSCs . n indicates the number of embryos transplanted.2n blastocyst injections. Numbers of animals obtained per total number of transferred embryos are shown.

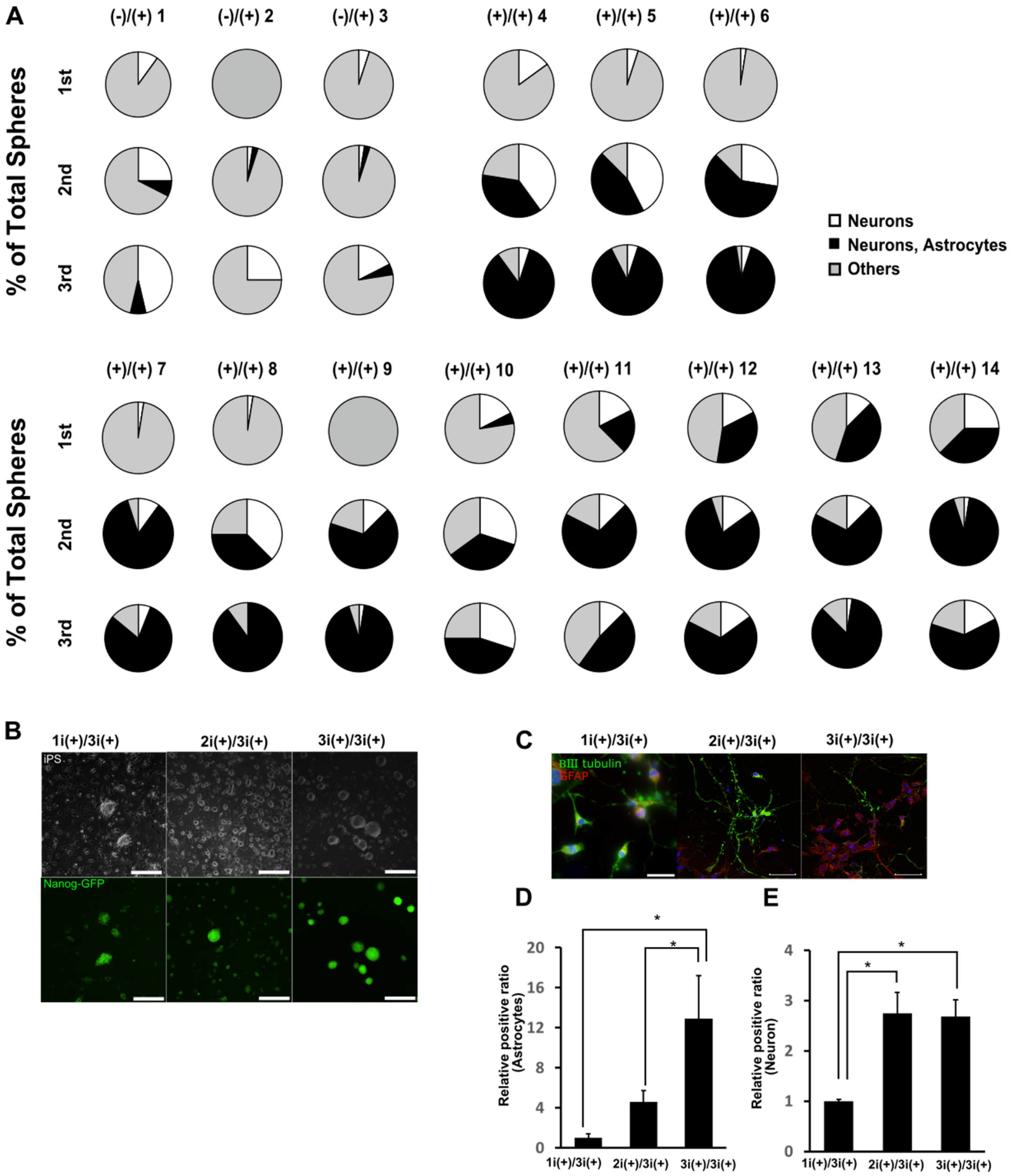

Figure S2. Differentiation of patterns in 3i, 2i and 1i- reprograming iPSCs

(A) Differentiation efficiency of the 1st, 2nd and 3rd neurospheres derived from (-)/(+) (clone 1-3) and (+)/(+) (clone 4-14) iPSCs. The frequency of colonies consisting of neurons (βⅢ-tubulin) and/or astrocytes (GFAP) was evaluated by immunocytochemistry and is presented as the percentage of positive colonies. (B) Morphology and *Nanog*-GFP expression of undifferentiated iPSC colonies reprogrammed with Cyto-Tune-iPS (MBL) in 1i (SU5402), 2i (PD184352 and CHIR99021) and 3i. Scale bar: 200 µm. (C) Differentiated neurons and astrocytes were detected by **βIII-tubulin** (green) and **GFAP** (red)

antibodies. Scale bar: 50μm. (D, E) The differentiation efficiency into astrocytes (D) and neurons (E) from 1i, 2i or 3i reprogrammed iPSCs (1i: n=6, 2i: n=9, 3i: n=8; *, $p < 0.05$). Immunostained cells were imaged and analyzed using an IN Cell Analyzer 2200 high contents cellular analysis system (GE Healthcare) as described previously (Fujimori et al., 2017).

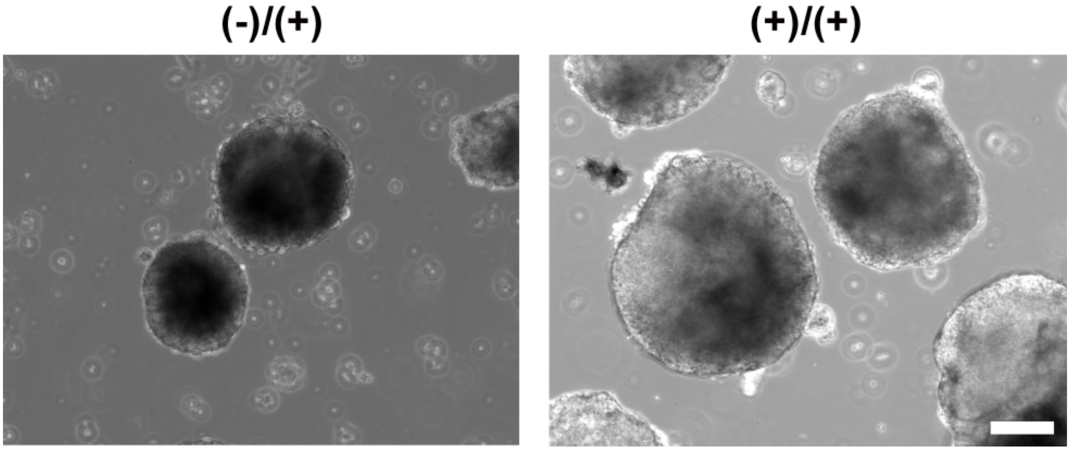

Figure S3. Representative morphology of EBs derived from (-)/(+) and (+)/(+) iPSC cell lines. Scale bar: 100 µm.

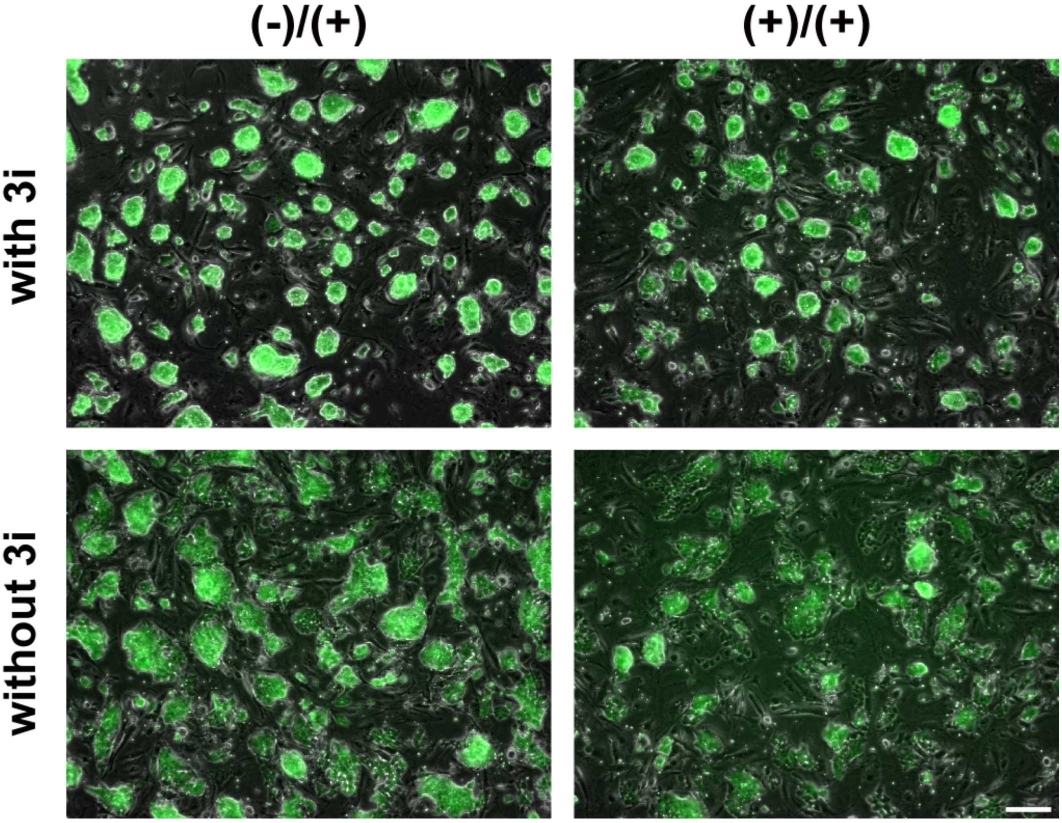

Figure S4. Representative colony morphology of (-)/(+) and (+)/(+) iPSC cell lines before or after withdrawal of 3i chemicals. Scale bar: 200 µm.

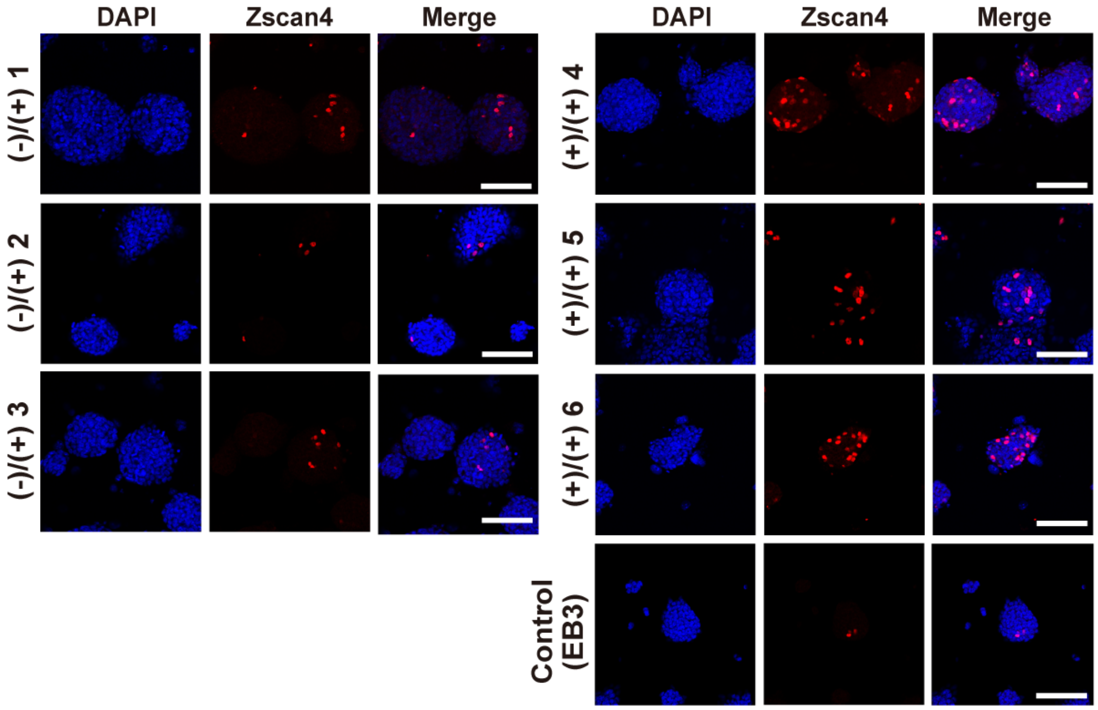

Figure S5. Representative images of immunocytochemistry of Zscan4 in (-)/(+) and (+)/(+) iPSCs (n=3, * ,$p < 0.05$). ESCs were used as controls.

Table S1. Region summary for CGH array

sample No.	Chr	Cytoband	Start	Stop	#Probes	Amplification	Deletion	pval	Gene Names
(-)/(+)_No.1	chr6	qF1 - qF3	120562739	1.25E+08	364	0.620126	0	0	Cecr2, Slc25a18, Atp6v1e1, Bcl2l13, Bid, Mical3, Pex26, Tuba8, Usp18, Slc6a13, Slc6a12, Iqsec3, A2m, Mug1, Cpamd8, Gm10319, Mug-ps1, Klrg1, M6pr, Phc1, Rimklb, Mfap5, Aicda, Apobec1, Gdf3, Dppa3, Nanog, Nanogpd, Slc2a3, Foxj2, C3ar1, Necap1, Clec4a1, Clec4a3, Clec4a4, Clec4b1, Clec4a2, Clec4b2, Clec4n, Clec4d, Clec4e, Vmn2r19, Vmn2r20, Vmn2r21, Vmn2r22, Vmn2r23, Vmn2r24, Vmn2r25, Vmn2r26, Vmn2r27, Cd163, Pex5, Clstn3, C1rl, C1r, C1s, C1rb, Gm5077, Lpcat3, Emg1, Phb2, Ptpn6, Grcc10, Rnu7, Atn1, Eno2, Lrrc23, Spsb2, Tpi1, Usp5, Cdca3, Gnb3, Leprel2, Gpr162, Cd4, Lag3, Ptms, Mlf2, Cops7a, C530028O21Rik, Zfp384, Ing4, Acrbp, Lpar5, Chd4, Nop2, Iffo1, Gapdh, Ncapd2, Mrpl51, Vamp1, Tapbpl, Cd27, 4930417O13Rik, Tuba3a, Ltbr, Scnn1a, Tnfrsf1a, Plekhg6
(-)/(+)_No.1	chr11	qA1 - qE2	3107005	1.22E+08	8984	0.555605	0	0	[illegible]
(-)/(+)_No.2	chr6	qB2.3 - qD3	49542982	96274002	3250	0	-0.81168	0	Npy, Mpp6, Dfna5, Osbpl3, Cycs, 5430402O13Rik, 4921507P07Rik, Npvf, Nfe2l3, Hnrnpa2b1, Cbx3, Snx10, Skap2, Hoxa1, Hoxa2, Hoxa3, Hoxa4, Hoxa5, Hoxa6, Hoxa7, Hoxa9, Hoxa10, Hoxa11, Hoxa11as, Hoxa13, Evx1, Hibadh, Tax1bp1, Jazf1, Creb5, 1200009O22Rik, Cpvl, Chn2, Prr15, Scrn1, Fkbp14, Plekha8, 2410066E13Rik, Znrf2, Nod1, Ggct, Gars, Crhr2, Inmt, Fam188b, Aqp1, Ghrhr, Adcyap1r1, Neurod6, Ccdc129, Gsbs, Pde1c, Lsm5, Avl9, Kbtbd2, Fkbp9, Nt5c3, V1rc21, V1rc19, V1rc20, V1rc32, V1rc30, V1rc1, V1rc3, Gm6674, V1rc5, V1rc7, V1rc6, V1rc29, V1rc16, V1rc26, V1rc27, Gm5569, Ppm1k, Herc5, Pigy, Lancl2, AW146242, V1rc28, V1rc23, V1rc24, V1rc18, V1rc8, V1rc17, V1rc33, V1rc25, V1rc2, V1rc22, Abcg2, Herc3, Nap1l5, Fam13a, Tigd2, Gprin3, Snca, Mmrn1, Grid2, Atoh1, Smarcad1, Hpgds, C130060K24Rik, Tnip3, A930038C07Rik, Prdm5, 4930544G11Rik, Mad2l1, V1rc15, V1rc14, V1rc12, V1rc11, V1rc10, V1rc13, Gng12, Gadd45a, Serbp1, Il12rb2, Il23r, Tacstd2, 4930515G16Rik, Rprl1, Rpia, Eif2ak3, 1700011F03Rik, Foxi3, Thnsl2, Fabp1, Smyd1, Krcc1, Cd8b1, Cd8a, Rmnd5a, Rnf103, Vps24, Kdm3a, Reep1, Mrpl35, Immt, Ptcd3, Polr1a, St3gal5, Atoh8, Sftpb, Usp39, 0610030E20Rik, Tmem150, Rnf181, Vamp5, Vamp8, Ggcx, Mat2a, Capg, Elmod3, Retsat, Tgoln1, Tgoln2, Tcf3, Kcmf1, Tmsb10, Suclg1, 4931417E11Rik, Ctnna2, Lrrtm1, Reg3b, Reg3d, Reg3a, Reg2, Reg1, Reg3g, Lrrtm4, AW146020, Mrpl19, Fam176a, Tacr1, Pole4, Hk2, Sema4f, D6Mm5e, Dok1, Loxl3, Htra2, Aup1, Dqx1, Tlx2, Pcgf1, Lbx2, Ccdc142, Mrpl53, Mogs, Wbp1, Ino80b, Rtkn, Wdr54, 1700003E16Rik, Dctn1, Mthfd2, Mobkl1b, Bola3, Dguok, Actg2, Stambp, Clec4f, Cd207, Vax2os2, Vax2os1, Vax2, Atp6v1b1, Ankrd53, Tex261, Nagk, Paip2b, Zfml, Dysf, Cyp26b1, Exoc6b, Npm3-ps1, Gm5878, Spr, Emx1, Sfxn5, Rab11fip5, Noto, Smyd5, 1700040I03Rik, Cct7, Fbxo41, Egr4, Alms1, Cml3, Cml5, Cml4, Cml2, 1700019G17Rik, Cml1, Tprkb, Dusp11, Figla, Add2, Tgfa, Fam136a, Snrpg, Pcyox1, Tia1, C87436, Pcbp1, Asprv1, Mxd1, Snrnp27, Gmcl1, Anxa4, 2610306M01Rik, Aak1, Nfu1, Gfpt1, Antxr1, Gkn1, Gkn2, 1190003M12Rik, Bmp10, Arhgap25, Prokr1, Aplf, Ccdc48, Gp9, Rab43, Isy1, Cnbp, Copg, 8430410A17Rik, H1fx, Gm5577, Rab7, Rpn1, Gata2, Dnajb8, Eefsec, Ruvbl1, Sec61a1, Klhdc6, Mgll, Abtb1, Podxl2, Mcm2, Tpra1, 4933427D06Rik, Gm1965, Gm839, Plxna1, Chchd6, Txnrd3, V1rb7, V1rb10, V1rb9, V1ra6, V1ra5, V1rb4, V1ra2, V1rb8, V1ra4, V1ra3, V1rb2, V1rb1, V1ra1, V1ra7, V1ra8, V1rb3, V1ra9, BC048671, Chst13, Uroc1, Zxdc, Ccdc37, Klf15, Aldh1l1, Slc41a3, Iqsec1, Nup210, Hdac11, Fbln2, Wnt7a, Chchd4, Tmem43, Xpc, Lsm3, Slc6a6, Grip2, C130022K22Rik, 4930590J08Rik, Fgd5, Nr2c2, Mrps25, Zfyve20, Trh, Prickle2, Adamts9, Magi1, Slc25a26, Lrig1, Kbtbd8, Suclg2, Fam19a1, 1700123L14Rik
(-)/(+)_No.3	chr14	qD3 - qE2.3	76841266	1.02E+08	1235	0.545352	0	0	Tsc22d1, Serp2, 1700108F19Rik, 9030625A04Rik, Ccdc122, Enox1, Gm6994, Gm1587, Dnajc15, Epsti1, AU021034, Tnfsf11, Dgkh, 1300010F03Rik, AU017455, 1190002H23Rik, Narg1l, Mtrf1, Kbtbd7, Gm5465, Wbp4, Elf1, Sugt1, Lect1, Pcdh8, Olfm4, Pcdh17, Diap3, Tdrd3, Pcdh20, Gm5088, Pcdh9, 4921530L21Rik, Klhl1, Dach1, 2410129H14Rik, 6720463M24Rik, Dis3, Pibf1, Klf5, Klf12, 1700110M21Rik
(+)/(+)_No.1	chr4	qC6	101455949	1.02E+08	9	0	-0.73734	2.52E-24	Lepr, B020004J07Rik
(+)/(+)_No.1	chr5	qA3	19051656	19551538	38	0	-2.94853	0	Magi2
(+)/(+)_No.1	chr8	qA1.1 - qE2	3130823	1.32E+08	8151	0.48887	0	0	Insr, A430078G23Rik, Arhgef18, Pex11c, 1700019B03Rik, Zfp358, Mcoln1, Pnpla6, C330021F23Rik, 2310057J16Rik, Xab2, Pcp2, Stxbp2, Retn, 1810033B17Rik, Trappc5, Fcer2a, Clec4g, Cd209a, Rprl3, Cd209e, Cd209d, Cd209b, Cd209c, Cd209f, Evi5l, BC068157, Lrrc8e, Map2k7, Snapc2, Ctxn1, Timm44, Elavl1, Ccl25, Lass4, BC003267, Shcbp1, Slc10a2, Efnb2, Arglu1, Fam155a, Lig4, Abhd13, Tnfsf13b, Myo16, Irs2, Col4a1, Col4a2, Rab20, Carkd, Cars2, Ing1, Ankrd10, 1700016D06Rik, Gm6524, Arhgef7, 1700018L24Rik, Gm5607, Sox1, 1700094C09Rik, Tubgcp3, Atp11a, Mcf2l, F7, F10, Proz, Pcid2, Cul4a, Lamp1, Grtp1, Adprhl1, Dcun1d2, Tmco3, Tfdp1, Atp4b, Grk1, Fam70b, Gas6, 1700029H14Rik, Rasa3, 4932443I19Rik, Cdc16, Upf3a, AF366264, Zfp828, 2410022L05Rik, Fbxo25, 2610019F03Rik, Erich1, Dlgap2, Cln8, Arhgef10, Kbtbd11, BB014433, Myom2, Csmd1, Mcph1, Angpt2, Agpat5, Xkr5, Defb40, Defb37, Defb38, Defb39, Defb12, Defb34, Spag11b, Spag11a, Defb14, Defb4, Defa-ps12, Defb6, Defb46, Defb5, Defb3, Defa-ps13, Defb8, Defb7, Gm6040, AY761185, Defa21, Defa-rs7, Defa23, Defa25, Defa22, Defa3, Defa5, Defcr-rs1, Defa20, Defa26, Defa4, Defa-ps1, AY761184, Defcr6, Defa24, Defb1, Defb50, Defb2, Defb10, Defb9, Defb11, Defb15, Defb35, Defb13, C86695, Ccdc70, Atp7b, Alg11, Nek5, Nek3, Ckap2, Vps36, Thsd1, Tpte, Slc25a15, Mrps31, AI316807, Slc20a2, Vdac3, Dkk4, Polb, A930013F10Rik, Ikbkb, Plat, Ap3m2, Myst3, Ank1, Nkx6-3, Agpat6, Gins4, Golga7, Sfrp1, Zmat4, 1810011O10Rik, Ido2, Ido1, Adam18, Adam3, Adam5, Adam32, Adam9, Tm2d2, Htra4, Plekha2, Tacc1, Fgfr1, Letm2, Whsc1l1, Ppapdc1b, Ddhd2, Bag4, Lsm1, Star, Ash2l, Kcnu1, Hgsnat, 4930444A02Rik, Fnta, Hook3, Rnf170, Thap1, Zfp703, Erlin2, Prosc, Gpr124, Brf2, Rab11fip1, Got1l1, Adrb3, Eif4ebp1, Gm9731, Tex24, Chrnb3, Chrna6, 4921537P18Rik, Unc5d, Dusp26, Rnf122, BC019943, Mak16, Fut10, Nrg1, Wrn, Purg, Tex15, Ppp2cb, Ubxn8, Gsr, Gtf2e2, Rbpms, Dctn6, Mboat4, Leprotl1, Tmem66, Dusp4, Tnks, Ppp1r3b, Eri1, Mfhas1, Cldn23, D8Ertd82e, Lonrf1, 6430573F11Rik, Dlc1, A730069N07Rik, AI429214, Sgcz, Tusc3, Msr1, Fgf20, Efha2, Zdhhc2, Cnot7, Vps37a, Mtmr7, Adam24, Adam25, Adam38, Adam39, Slc7a2, Pdgfrl, Mtus1, Fgl1, Pcm1, Asah1, Frg1, Zfp353, Triml1, Triml2, Zfp42, Adam26b, Adam26a, Gm5346, Adam34, Gm5347, Fat1, Mtnr1a, F11, Klkb1, Cyp4v3, Fam149a, Tlr3, Sorbs2, Pdlim3, Ccdc110, 1700029J07Rik, Ufsp2, Ankrd37, Lrp2bp, Snx25, 4933411K20Rik, Slc25a4, Helt, Acsl1, Mlf1ip, Ccdc111, Casp3, Irf2, Enpp6, Stox2, D030016E14Rik, Rwdd4a, Ing2, Cdkn2aip, LOC100039801, Cldn22, Wwc2, Dctd, Odz3, Aga, Neil3, Vegfc, Spcs3, Asb5, Spata4, Wdr17, Gpm6a, Adam29, Glra3, Hpgd, BC088983, Fbxo8, Hand2, Scrg1, Sap30, Hmgb2, Galnt7, 4930431L04Rik, BC030500, Aadat, Mfap3l, 2700029M09Rik, Clcn3, Nek1, Sh3rf1, Cbr4, Palld, Ddx60, Anxa10, Spock3, Tll1, Cpe, Sc4mol, Klhl2, Tmem192, Trim75, Trim60, Trim61, March1, 1810029B16Rik, Tktl2, Npy5r, Npy1r, Naf1, Nat1, Nat2, Nat3, Psd3, Sh2d4a, Csgalnact1, Ints10, Lpl, Slc18a1, Atp6v1b2, Lzts1, D10627, D130040H23Rik, Zfp868, Zfp869, D330038O06Rik, Atp13a1, Gmip, Lpar2, Pbx4, Cilp2, Ndufa13, Tssk6, Gatad2a, 9130404D08Rik, Sf4, Tm6sf2, Hapln4, Ncan, Rfxank, Nr2c2ap, 2310045N01Rik, Mef2b, Tmem161a, Slc25a42, Armc6, Sfrs14, Homer3, Ddx49, Cope, Gdf1, Lass1, Upf1, Comp, Crtc1, Klhl26, Tmem59l, Crlf1, 2810428I15Rik, Uba52, Gm11808, 2810422J05Rik, Fkbp8, Ell, Isyna1, Ssbp4, Lrrc25, Gdf15, Pgpep1, Lsm4, Jund, Pde4c, Rab3a, Mpv17l2, Ifi30, Pik3r2, Il12rb1, Arrdc2, Kcnn1, Ccdc124, Slc5a5, Rpl18a, Gm15427, Snora68, Mtap1s, Haus8, Myo9b, Use1, Ocel1, Nr2f6, Ushbp1, 5430437P03Rik, Ankle1, Abhd8, Mrpl34, Dda1, Gtpbp3, Plvap, Bst2, Fam125a, Tmem221, Nxnl1, Slc27a1, Pgls, Glt25d1, Unc13a, Jak3, Insl3, B3gnt3, Fcho1, Zfp709, Zfp617, Cyp4f18, Olfr372, Olfr373, Olfr374, Tpm4, Rab8a, Hsh2d, 2510049I19Rik, Ap1m1, Klf2, Eps15l1, Calr3, 1700030K09Rik, Cherp, Slc35e1, Med26, 9130011J15Rik, Tmem38a, Nwd1, Sin3b, F2rl3, Large, Isx, Hmgxb4, Tom1, Hmox1, Mcm5, Rasd2, Nr3c2, Arhgap10, 0610038B21Rik, Prmt10, Tmem184c, Ednra, Ttc29, Pou4f2, Rbmxrt, Slc10a7, Lsm6, Zfp827, 1700011L22Rik, Mmaa, Smad1, Otud4, Abce1, Anapc10, Hhip, Gypa, Smarca5, Gab1, Usp38, Inpp4b, Il15, Zfp330, Rnf150, Tbc1d9, Ucp1, Elmod2, 4933434I20Rik, Clgn, Scoc, Olfr370, Ndufb7, Tecr, Dnajb1, Gipc1, Ptger1, Pkn1, Ddx39, Cd97, Lphn1, Asf1b, Prkaca, Samd1, 1700067K01Rik, 4432412L15Rik, Il27ra, Rln3, Rfx1, Dcaf15, Podnl1, Cc2d1a, 4930432K21Rik, Nanos3, Zswim4, D8Ertd738e, Mri1, Ccdc130, Cacna1a, Ier2, Nacc1, Trmt1, Lyl1, Nfix, Dand5, Gadd45gip1, Rad23a, Calr, Farsa, Syce2, Gcdh, Klf1, Dnase2a, Mast1, Rtbdn, Rnaseh2a, Prdx2, Junb, Hook2, Best2, Asna1, 2310036O22Rik, Tnpo2, Fbxw9, Dhps, 1500041N16Rik, BC056474, Man2b1, Zfp791, Gm6531, Olfr371, Vps35, Orc6l, Mylk3, 4921524J17Rik, Gpt2, Dnaja2, Neto2, Itfg1, Phkb, Abcc12, Lonp2, Siah1a, Gm10638, N4bp1, Cbln1, 4933402J07Rik, Zfp423, Tmem188, Heatr3, Adcy7, Brd7, Nkd1, Snx20, Nod2, Cyld, Sall1, Tox3, Chd9, Rbl2, Aktip, Rpgrip1l, Fto, Irx3, Irx5, Irx6, Mmp2, Lpcat2, Capns2, Slc6a2, Gm4976, Gm5158, Es1, Ces3, Es22, AU018778, Ces1, Ces7, Gnao1, 4930488L21Rik, Amfr, Nudt21, Ogfod1, Bbs2, Mt4, Mt3, Mt2, Mt1, Nup93, Slc12a3, Herpud1, Nlrc5, Cpne2, 2310065K24Rik, Rspry1, Arl2bp, Pllp, Ccl22, Cx3cl1, Ccl17, Ciapin1, Coq9, Polr2c, Dok4, Ccdc102a, Gpr114, Gpr56, Gpr97, Ccdc135, Katnb1, Kifc3, BC016201, Tepp, Zfp319, AA960436, Mmp15, Gtl3, Csnk2a2, Ccdc113, 4931432M23Rik, Gins3, Ndrg4, Setd6, Cnot1, Slc38a7, Got2, Cdh8, Cdh11, Cdh5, Bean, Tk2, Cklf, Cmtm2a, Cmtm2b, Cmtm3, Cmtm4, Dync1li2, Ccdc79, Nae1, Car7, Pdp2, Cdh16, Rrad, Fam96b, Ces6, BC015286, Ces2, Ces5, 2310038E17Rik, 2210023G05Rik, Gm4738, Ces8, Cbfb, D230025D16Rik, B3gnt9, Tradd, Fbxl8, Hsf4, Nol3, 4931428F04Rik, Exoc3l, E2f4, Elmo3, Lrrc29, Tmem208, Fhod1, Slc9a5, Plekhg4, Kctd19, Lrrc36, Tppp3, Zdhhc1, Hsd11b2, Atp6v0d1, Agrp, Fam65a, Ctcf, Rltpr, Acd, Pard6a, E130303B06Rik, 4933405L10Rik, Gfod2, Ranbp10, Tsnaxip1, Cenpt, Thap11, Nutf2, Edc4, Nrn1l, Pskh1, Ctrl, Psmb10, Lcat, Slc12a4, Dpep3, Dpep2, Ddx28, Dus2l, Nfatc3, Esrp2, Pla2g15, Slc7a6, Slc7a6os, Prmt7, Smpd3, Zfp90, Cdh3, Cdh1, Tmco7, Has3, Chtf8, Cirh1a, Sntb2, Vps4a, Pdf, Cog8, Nip7, Tmed6, Terf2, Cyb5b, Nfat5, Nqo1, Nob1, Wwp2, Gm10070, Psmd7, Zfhx3, Gm1943, Pmfbp1, Dhx38, Txnl4b, Hp, Dhodh, Pkd1l3, 2400003C14Rik, Zfp821, Atxn1l, Ap1g1, Phlpp2, Marveld3, Tat, Chst4, Zfp612, Calb2, Ftsjd1, Hydin, Vac14, Mtss1l, Il34, Sf3b3, Cog4, Fuk, St3gal2, Ddx19a, Ddx19b, Aars, Exosc6, Clec18a, 4930402E16Rik, Glg1, Rfwd3, Mlkl, Fa2h, Wdr59, Znrf1, Ldhd, Zfp1, Ctrb1, Bcar1, Cfdp1, Tmem170, Chst5, 4932417I16Rik, Gabarapl2, Adat1, Kars, Terf2ip, Cntnap4, Mon1b, 4930481F22Rik, Adamts18, Nudt7, Vat1l, Clec3a, Wwox, Maf, Dynlrb2, Cdyl2, 2310061C15Rik, Cenpn, Atmin, 1700030J22Rik, Gcsh, Pkd1l2, Bcmo1, Gan, 4933407C03Rik, Plcg2, Sdr42e1, Hsd17b2, Mphosph6, Cdh13, Hsbp1, Mlycd, Osgin1, Necab2, Slc38a8, Mbtps1, Hsdl1, Lrrc50, Taf1c, Adad2, Kcng4, Wfdc1, Atp2c2, 4632415K11Rik, Cotl1, Klhl36, Usp10, Crispld2, Zdhhc7, 6430548M08Rik, Fam92b, Gse1, Gins2, 1190005I06Rik, Cox4nb, Cox4i1, Irf8, Foxf1a, Mthfsd, Foxc2, Foxl1, 1700018B08Rik, Fbxo31, Map1lc3b, Zcchc14, Jph3, Klhdc4, Slc7a5, BC048644, Car5a, Banp, Zfpm1, Trhr2, Zc3h18, Il17c, Cyba, Mvd, Snai3, Rnf166, 2310061F22Rik, Cdt1, Aprt, Galns, Trappc2l, Pabpnl1, Cbfa2t3, Acsf3, Gm16378, Cdh15, Ankrd11, Spg7, Rpl13, Snord68, Cpne7, Sult5a1, Dpep1, Chmp1a, 4732415M23Rik, Cdk10, Spata2L, 1300018I17Rik, Zfp276, Fanca, Spire2, Tcf25, Mc1r, Tubb3, Def8, Afg3l1, Dbndd1, Gas8, Rhou, Rab4a, 1700054N08Rik, Acta1, Nup133, Abcb10, Taf5l, Urb2, Galnt2, Pgbd5, Cog2, Agt, Capn9, 2310022B05Rik, Ttc13, Arv1, Fam89a, Trim67, 2810004N23Rik, Gnpat, Exoc8, Gm505, Egln1, Tsnax, Disc1, Sipa1l2, 4933403G14Rik, 2310079N02Rik, Pcnxl2, BC021891, Kcnk1, Slc35f3, 1810063B05Rik, Tarbp1, Tomm20, Rbm34, Pard3, Nrp1, Itgb1, Ccdc7, 1700008F21Rik
(+)/(+)_No.1	chrY	qA1	37653	2635638	121	0	-0.52334	7.71E-162	Zfy1, Ube1y1, Kdm5d, Eif2s3y, Tspy-ps, Uty, Ddx3y, Usp9y, Zfy2, Gm16501, Sry, Rbmy1a1
(+)/(+)_No.2	chr5	qE1	90304287	90321465	3	0	-0.94994	7.67E-14	Adamts3
(+)/(+)_No.2	chr11	qA1 - qE2	3030764	1.22E+08	8992	0.399104	0	0	[illegible]
(+)/(+)_No.2	chr15	qD3	73018495	73298481	28	0	-0.74953	5.78E-78	Ptk2
(+)/(+)_No.2	chrY	qA1	37653	2529532	117	0	-3.04195	0	Zfy1, Ube1y1, Kdm5d, Eif2s3y, Tspy-ps, Uty, Ddx3y, Usp9y, Zfy2, Gm16501, Sry, Rbmy1a1
(+)/(+)_No.2	chrY	qA1	253207	273823	4	0	-5.35883	3.61E-16	Kdm5d
(+)/(+)_No.2	chrY	qA1	346878	374359	5	0	-4.98972	2.28E-16	Eif2s3y
(+)/(+)_No.2	chrY	qA1	434833	731591	40	0	-4.17578	2.55E-54	Uty, Ddx3y, Usp9y
(+)/(+)_No.2	chrY	qA1	801912	1356694	13	0	-4.59496	9.90E-29	
(+)/(+)_No.3	chr12	qC3	76835388	76958575	14	0.782565	0	3.08E-33	Sgpp1, Syne2

Cell	Methods	Total reads	mapped reads		unmapped reads	
mouse-iPS	(-)/(+)	53773236	45988390	85.52%	7784846	14.48%
mouse-iPS	(-)/(+)	57806362	49041112	84.84%	8765250	15.16%
mouse-iPS	(-)/(+)	54591766	47091620	86.26%	7500146	13.74%
mouse-iPS	(+)/(+)	55474387	47075833	84.86%	8398554	15.14%
mouse-iPS	(+)/(+)	51188601	43300760	84.59%	7887841	15.41%
mouse-iPS	(+)/(+)	55114847	46748840	84.82%	8366007	15.18%
fibroblast		50539652	43232627	85.54%	7307025	14.46%

Table S2. Sequencing summary for MBD-seq

Total reads: total number of reads for raw MBD-seq data in each sample. Mapped reads: number and percentage of MBD-seq reads mapped to mouse genome reference sequence (mm9). Unmapped reads: number and percentage of MBD-seq reads unmapped to mouse genome reference sequence (mm9).

Top Bio Functions		
**Diseases and Disorders**	**p-value**	**# of Molecules**
Cancer	4.86E-06 - 1.09E-02	166
Organismal Inury and Abnormalities	1.18E-05 - 1.02E-02	58
Inflammatory Disease	3.01E-05 - 1.02E-02	16
Respiratory Disease	3.01E-05 - 6.91E-03	43
Connective Tissue Disorders	7.68E-05 - 1.02E-02	40
**Molecular and Cellular Functions**		
Cell Morphology	1.52E-06 - 1.04E-02	69
Cellular Assembly and Organization	1.52E-05 - 1.04E-02	75
Cell Cycle	9.12E-05 - 1.02E-02	66
Cell-To-Cell Signaling and Interaction	1.72E-05 - 1.10E-02	49
Cellular Compromise	1.99E-05 - 1.10E-02	24
**Physiological System Development and Function**		
Organismal Functions	1.24E-07 - 9.28E-03	27
Tissue Morphology	1.24E-07 - 1.04E-02	68
Cardiovascular System Development and Function	2.98E-07 - 1.02E-02	75
Organismal Development	2.64E-06 - 1.10E-02	122
Tissue Development	1.72E-05 - 1.10E-02	120

Table S3. Gene ontology analysis of differentially expressed genes (Data represent mean ± SD of three biological replicates).

**Gene name**	**Fowrard primer**	**Reverse primer**
Nanog	**AGGACAGGTTTCAGAAGCAGA**	**CCATTGCTAGTCTTCAACCACTG**
Oct3/4	**CGGAAGAGAAAGCGAACTAGC**	**ATTGGCGATGTGAGTGATCTG**
Klf4 (Tg)	**GGACCACCTTGCCTTACACA**	**TGGCCTGCCCGGTTATTATT**
Sox2 (Tg)	**GGTTACCTCTTCCTCCCACTCCAG**	**TTATCGTCGACCACTGTGCTGCTG**
c-Myc (Tg)	**CAGAGGAGGAACGAGCTGAAGCGC**	**TTATCGTCGACCACTGTGCTGCTG**
Oct3/4 (Tg)	**CTTTCCCTCTGTTCCCGTCA**	**GGTAATGGTAGCGACCGGC**
Oct3/4 (endo)	**ATTCCCAACGAGAAGAGTATGA**	**TCCCTTGCCTTGGCTCACAG**
β-actin	**GAAATCGTGCGTGACATCAAAG**	**TGTAGTTTC ATGGATGCCACAG**
Nestin	**CCCCTTGCCTAATACCCTTGA**	**GCCTCAGACATAGGTGGGATG**
Pax6	**TACCAGTGTCTACCAGCCAAT**	**TGCACGAGTATGAGGAGGTCT**
Isl1	**ATGATGGTGGTTTACAGGCTAAC**	**TCGATGCTACTTCACTGCCAG**
Sox17	**GATGCGGGATACGCCAGTG**	**CCACCACCTCGCCTTTCAC**
FoxA2	**TCCGACTGGAGCAGCTACTAC**	**GCGCCCACATAGGATGACA**
FoxC1	**CCCCGGACAAGAAGATCACTC**	**AGGTTGTGCCGTATGCTGTTC**
Flk-1	**TTTGGCAAATACAACCCTTCAGA**	**GCAGAAGATACTGTCACCACC**
Zscan4	**CCTATGTAGCCGTCGCTTTC**	**AAGTGGCAATCCACAAGCAT**
muERV-L	**CCCATCATGAGCTGGGTACT**	**CGTGCAGAGCCATCAGTAAA**
Tcstv1	**GCCCAGAGTACAAGGTGTTCTAAT**	**ATTCAATCTTCGGTAGGATCTCAG**
Eif1a	**AAGAAGTCTGAAGGCCTATG**	**CAGAGAACTTGGAAGGTAGC**

Table S4. Primers used in this study.

Antibody	Source	Catalogue number
Nanog	Abcam	ab80892
Oct3/4	Santa Cruz	SC5279
Oct4	Santa Cruz	sc9081
SSEA1	Abcam	MC480
SSEA3	Abcam	MC631
SSEA-4	Abcam	MC813
TRA-1-60	Millipore	MAB4360
TRA-1-81	Millipore	MAB4381
Zscan4	Abnova	H00201516-B01P
EIf1a	Abcam	ab172623 (177939)
β -III-Tubulin	Sigma	T8660
GFAP	Dako	N1506

Table S5. Antibodies used in this study.
